# Generalization of Bleaney’s Theory

**DOI:** 10.1021/acs.jpca.6c01791

**Published:** 2026-06-18

**Authors:** Lucas Lang, Bryan Lauw, Letizia Fiorucci

**Affiliations:** † 26524 Technische Universität Berlin, Institut für Chemie, Theoretische Chemie/Quantenchemie, Sekr. C7, Straße des 17. Juni 135, 10623 Berlin, Germany; ‡ Max-Planck-Institut für Kohlenforschung, Kaiser-Wilhelm-Platz 1, 45470 Mülheim an der Ruhr, Germany

## Abstract

We generalize and unify Bleaney’s theory and related inverse-temperature expansions of magnetic properties of paramagnetic species. Our approach is valid for different properties, including NMR chemical shifts beyond the point-dipole approximation, and for both transition-metal and lanthanide complexes. We derive an analytical equation for the 1/*T* ^3^ term. Furthermore, we implement higher-order terms numerically to investigate the convergence behavior of 1/*T* expansions. For transition-metal complexes, the second-order and third-order expansions provide quantitatively accurate results for most commonly encountered zero-field splittings. For an isostructural series of lanthanide complexes, the newly derived third-order term substantially improves the accuracy of calculated susceptibility anisotropies compared with Bleaney’s second-order theory.

## Graphical abstract

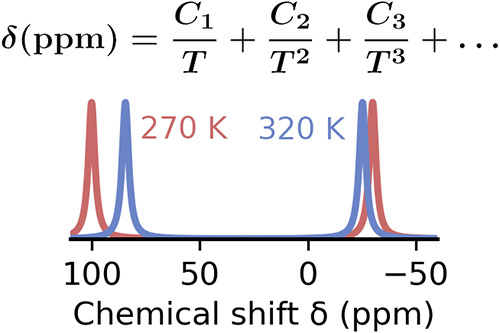

## 1 Introduction

In 1972, Bleaney proposed equations for lanthanide (Ln) pseudocontact NMR shifts in the point-dipole approximation, which depend on susceptibility anisotropies. These equations are valid up to order 1/*T* ^2^, which constitutes a high-temperature approximation.^1^ The theory predicts that the axial and rhombic susceptibility anisotropies are proportional to the Stevens^2^ crystal-field/ligand-field parameters *B*_2_^0^ and *B*_2_^2^, with a proportionality constant that depends only on the Ln ion and not on the crystal environment or ligands. This theory has been used extensively to interpret NMR spectra of Ln compounds and the associated susceptibility anisotropies over many decades.^3–12^ Recently, an approach related to Bleaney’s theory was used to derive a “rule of thumb” for estimating the magnitude of the field dependence of NMR shifts in Ln complexes.^13^

However, doubts regarding the validity of Bleaney’s theory have accumulated during the same time. Early work investigating the exact temperature dependence of the susceptibility,^14–16^ as well as analytical work investigating the next-order 1/*T* ^3^ correction,^17–19^ concluded that Bleaney’s equations lead to errors of at most 10–20% and describe the susceptibility in a qualitatively correct way.

In the early 2000s, Binnemans and co-workers published further theoretical investigations of Bleaney’s theory using the numerical solution for the susceptibility valid up to infinite order in 1/*T*, investigating model complexes with various coordination polyhedra.^20,21^ In conflict with earlier work, they found that Bleaney’s theory has severe limitations: in many of their examples, the errors far exceeded 10–20% and results were not even qualitatively correct. They explained the discrepancy with the size of the crystal-field/ligand-field splitting of the systems investigated in the earlier work, which was much smaller than *k*_B_ *T* at room temperature and not representative for all Ln­(III) complexes. A good overview of the status of Bleaney’s theory in the early 2000s is available in reference 22.

Recently, a number of groups picked up the topic again and performed theoretical and experimental investigations of the validity of Bleaney’s theory. In 2015, Parker and co-workers investigated isostructural Ln­(III) series formed by multidentate organic ligands in solution and found severe limitations of Bleaney’s theory.^23^ In 2016, Castro et al. investigated another isostructural series of Ln­(III) ions with a multidentate macrocyclic ligand and found that Bleaney’s theory is violated for at least two of the complexes of the series.^24^ In 2017, Suturina et al. further investigated one of the series from the 2015 work and found that the main axis of the susceptibility tensor changes by 90° along the series, which is incompatible with the transferability of ligand-field parameters often assumed in connection with Bleaney’s theory.^25^ In addition, they experimentally observed beyond-1/*T* ^2^ temperature dependence of the chemical shifts in one complex.

Studies on Bleaney’s theory are complicated by the fact that a large number of approximations and assumptions are often associated with it. The most fundamental assumption is that the splitting of the field-free energy levels is small enough compared to *k*_B_ *T* that a truncation of the expansion at order 1/*T* ^2^ gives accurate results. Furthermore, it is usually assumed that *L*, *S*, *J* are all good quantum numbers, i.e., that there is negligible mixing of different *LS* terms by spin–orbit coupling (SOC) and negligible mixing of different *J*-manifolds by the crystal field/ligand field. These assumptions, together with the assumption that the *f*-orbitals possess full spherical symmetry, lead to the conclusion that the **g**-tensors of Ln complexes are isotropic and given by the Landé *g*-factors of the free ions. These assumptions also lead to the tabulated values of Bleaney’s constants *C*_*J*_ and the ability to express the susceptibility in terms of Stevens parameters parametrizing the one-electron crystal field/ligand field. Moreover, when pseudocontact NMR shifts are discussed, the point-dipole approximation is usually employed, which leads to an expression for the isotropic shifts in terms of the axial and rhombic susceptibility anisotropies of the complex. It is also often assumed that the crystal-field/ligand-field parameters are transferable across an isostructural series. Additional assumptions sometimes associated with Bleaney’s theory are the neglect of higher than second-order ligand-field parameters,^23,26,27^ and the assumption that the main axis of the susceptibility tensor aligns with the main molecular *C*_*n*_ axis.^27^ As we will see below, these last two assumptions are not necessary for the validity of Bleaney’s theory.

Expansions in orders of 1/*T* are also common for magnetic properties of transition-metal (TM) complexes.^28,29^ Here, we investigate the most fundamental idea underlying Bleaney’s theory, i.e., the expansion of magnetic properties of both Ln and TM complexes in powers of 1/*T*. We analytically derive all terms up to 1/*T* ^3^ and investigate higher-order terms numerically. This sheds light on the validity of expansions truncated at second and third order. Our derivation focuses on the angular momentum dyadic (or spin dyadic) instead of the susceptibility tensor, which makes it also directly applicable to paramagnetic chemical shielding tensors beyond the point-dipole approximation.

## 2 Theory

### 2.1 The Effective Hamiltonian and Magnetic Properties

We assume that the paramagnetic molecule of interest can be described by a (2*J* + 1)-dimensional model Hamiltonian, where *J* ≥ 1/2 is an angular momentum quantum number. For TM complexes, we write *S* instead of *J*, which is the total spin quantum number (e.g., *S* = 1 for a *d* ^8^ nickel­(II) complexes with two unpaired electrons) and call the model Hamiltonian a “spin Hamiltonian”. For Ln complexes, *J* is the total (spin + orbital) angular momentum quantum number. The model Hamiltonian is expressed in terms of angular momentum operators

$$H=H^{\left(0\right)}+\mu_{B}B^{T}\mathrm{gJ}+\underset{K}{\sum }I_{K}^{T}A_{K}J$$ 1

Here, **J** = (*J*_*x*_, *J*_*y*_, *J*_*z*_)^T^ is the angular momentum vector, where each of the components *J*_*i*_ are represented by (2*J* + 1) × (2*J* + 1)-dimensional matrices. Again, for TM complexes, we write **S** instead of **J**. *H* ^(0)^ is the Hamiltonian in the absence of an external magnetic field and the second term parametrizes the Zeeman interaction, i.e., the interaction of the system with a homogeneous external magnetic field with magnetic induction vector **B**. It is parametrized by the **g**-tensor. For TM complexes, the **g**-tensor is typically anisotropic, but for Ln complexes, it can often be assumed to be proportional to the unit matrix, **g** = *g*_*J*_ **I**. Here, *g*_*J*_ is the so-called Landé *g*-factor, which is tabulated for each Ln ion.^1^ The third term describes the hyperfine interaction with the nuclei *K* having nuclear spin operators **I**_*K*_ and is parametrized by the hyperfine coupling tensors **A**_*K*_.

In the present work, we use the spherical tensor operators (STOs) *O*_*q*_^(*k*)^ introduced by Buckmaster^30^ and Smith and Thornley^31^ (BST) as a basis for expanding the field-free Hamiltonian

$$H^{\left(0\right)}=\underset{k=2,4,\cdot \cdot \cdot ,2J}{\sum }\overset{k}{\underset{q=-k}{\sum }}{\left(-1\right)}^{q}B_{-q}^{\left(k\right)}O_{q}^{\left(k\right)}=\underset{k=2,4,\cdot \cdot \cdot ,2J}{\sum }\overset{k}{\underset{q,q\prime =-k}{\sum }}\left(\begin{array}{c} k\\ q\;q^{\prime } \end{array}\right)B_{q\prime }^{\left(k\right)}O_{q}^{\left(k\right)}$$ 2

Note that, since the Hamiltonian must be Hermitian and (*O*_*q*_^(*k*)^)^†^ = (−1)^*q*^ *O*_–*q*_^(*k*)^,^32^ the parameters must satisfy the constraint (*B*_*q*_^(*k*)^)* = (−1)^*q*^ *B*_–*q*_^(*k*)^. The “antisymmetric symbol” is defined as

$$\left(\begin{array}{c} k\\ q\;q^{\prime } \end{array}\right)={\left(-1\right)}^{k+q}\delta_{q,-q\prime }$$ 3

and since *k* is even (see below), one can simplify the sign factor to (−1)^*k*+*q*^ = (−1)^*q*^. We write the Hamiltonian in terms of the antisymmetric symbol because the latter has a simple graphical representation that will become convenient for us. Note that for the exact parametrization of an arbitrary (2*J* + 1)-dimensional model Hamiltonian, all even *k* up to 2*J* are required. Odd *k* are not required because *H* ^(0)^ is even under time reversal.^33^ The *k* = 0 term shifts the complete spectrum by a constant, which has no effect on observable properties. Therefore, we exclude the *k* = 0 term, which means that the field-free Hamiltonian is traceless. Here and in the following, we will also assume that *J* ≥ 1/2, i.e., that we have an open-shell case with at least two states in the model space. Note that the *J* = 1/2 case is trivial: in this case, the field-free Hamiltonian vanishes, in agreement with Kramers degeneracy.

The BST operators with even *k* between 0 and 2*J* form a complete basis in which any field-free (2*J* + 1)-dimensional spin Hamiltonian can be expanded. Matrix elements of the BST operators can be expressed using the Wigner–Eckart theorem

4 $$\left\langle \mathrm{JM}\right|O_{q}^{\left(k\right)}\left|JM^{\prime }\right\rangle =\left(\begin{array}{ll} J & k\\ M^{\prime } & q \end{array}|\begin{array}{l} J\\ M \end{array}\right)\left\langle J∥O^{\left(k\right)}∥J\right\rangle$$

as a product of a Clebsch–Gordan coefficient (CGC) and the reduced matrix element^31^

5 $$\left\langle J∥O^{\left(k\right)}∥J\right\rangle =\frac{1}{2^{k}}\sqrt{\frac{1}{2J+1}\frac{\left(2J+k+1\right)!}{\left(2J-k\right)!}}$$

The parametrization in eq 2 can be used for both Ln and TM complexes. Note however that there is a quite different physical interpretation in both cases. In the TM case, where the BST operators are constructed from spin operators, *H* ^(0)^ describes zero-field splitting due to spin-dependent effects like SOC. In the Ln case, the BST operators are constructed from the *total* (spin + orbital) angular momentum operators, and they describe the splitting of the lowest *J*-manifold due to the nonspherically symmetric ligand field. We discuss these two types of systems in more detail below. Also note that our definition of the field-free Hamiltonian is slightly different than the usual parametrization in terms of STOs found in the literature: in our case, the parameters *B*_*q*_^(*k*)^ transform like components of a spherical tensor. In the section about Ln complexes below, we discuss their relationship to the widespread Wybourne and Stevens parametrizations.

The susceptibility tensor can be expressed as

6 $$\chi =-\mu_{0}{\left(\frac{\partial^{2}F}{\partial B\partial B}\right)}^{\left(0\right)}=-\mu_{0}\left\langle \left\langle \frac{\partial H}{\partial B}{\frac{\partial H}{\partial B}}^{T}\right\rangle \right\rangle =-\mu_{B}^{2}\mu_{0}g\left\langle \left\langle {\mathrm{JJ}}^{T}\right\rangle \right\rangle g^{T}$$

where *F* = −*k*_B_ *T *ln *Z* (*Z* = tr­(e^–β*H*^)) is the electronic Helmholtz free energy and the *angular momentum dyadic* ⟨⟨**JJ** ^T^⟩⟩ is a symmetric 3 × 3 tensor that depends on the field-free Hamiltonian *H* ^(0)^ and the temperature *T* (or, equivalently, the inverse temperature β = 1/*k*_B_ *T*).^34^ For Ln complexes with isotropic *g*_*J*_, the equation for the susceptibility tensor simplifies to

7 $$\chi =-\mu_{B}^{2}g_{J}^{2}\mu_{0}\left\langle \left\langle {\mathrm{JJ}}^{T}\right\rangle \right\rangle$$

i.e., the susceptibility tensor and the angular momentum dyadic are proportional. The hyperfine shielding tensor of nucleus *K* can be expressed in terms of the angular momentum dyadic as

8 $$\sigma_{K}={\left(\frac{\partial^{2}F}{\partial B\partial M_{K}}\right)}^{\left(0\right)}=\left\langle \left\langle \frac{\partial H}{\partial B}{\frac{\partial H}{\partial M_{K}}}^{T}\right\rangle \right\rangle =\frac{\mu_{B}}{\gamma_{K}}g\left\langle \left\langle {\mathrm{JJ}}^{T}\right\rangle \right\rangle A_{K}^{T}$$

where **M**_K_=γ_K_ **I**_K_ are the nuclear magnetic moments. More generally, for a model Hamiltonian of the form eq 1, any temperature-dependent magnetic property that is a second derivative of *F* can be expressed in terms of ⟨⟨**JJ** ^T^⟩⟩ and parameter tensors (as long as the perturbations are linear in the angular momentum operators). A “thermal linear response function” can be expressed as^33,34^

9 $$\left\langle \left\langle H_{k}H_{l}\right\rangle \right\rangle =-\frac{1}{Z}\int_{0}^{\beta }\mathrm{tr}\left(H_{k}e^{-\left(\beta -\omega \right)H}H_{l}e^{-\omega H}\right)d\omega$$

where *H*_*k*_ and *H*_*l*_ are arbitrary operators. If they are first derivatives of the Hamiltonian, *H*_*k*_ = ∂*H*/∂λ^*k*^ and *H*_*l*_ = ∂*H*/∂λ^*l*^, if all derivatives of the Hamiltonian beyond first order vanish, and if the Hamiltonian is field-free, then these linear response functions (or “dyadics”) are equal to second derivatives of the Helmholtz free energy evaluated at zero field

10 $${\left(\frac{\partial^{2}F}{\partial \lambda^{k}\partial \lambda^{l}}\right)}^{\left(0\right)}=\left\langle \left\langle H_{k}H_{l}\right\rangle \right\rangle$$

Instead of the basis-independent eq 9, it is more common to employ the eigenbasis of the field-free Hamiltonian

11 $$H^{\left(0\right)}\left|M\mu \right\rangle =E_{M}\left|M\mu \right\rangle$$

where μ labels different states with the same energy in the case of degeneracies. In this eigenbasis

12 $$\left\langle \left\langle H_{k}H_{l}\right\rangle \right\rangle =-\frac{1}{Z}\sum_{M}e^{-\beta E_{M}}\left[\sum_{\mu \mu \prime }\beta \left\langle M\mu \right|H_{k}\left|M\mu^{\prime }\right\rangle \left\langle M\mu^{\prime }\right|H_{l}\left|M\mu \right\rangle +\sum_{\mu }\sum_{N\neq M,\nu }\frac{\left\langle M\mu \right|H_{k}\left|N\nu \right\rangle \left\langle N\nu \right|H_{l}\left|M\mu \right\rangle +\left\langle M\mu \right|H_{l}\left|N\nu \right\rangle \left\langle N\nu \right|H_{k}\left|M\mu \right\rangle }{E_{N}-E_{M}}\right]$$

However, it will be more convenient for us to use eq 9 as a starting point for deriving high-temperature approximations of the dyadic.

### 2.2 Derivatives of the Angular Momentum Dyadic

The exact thermal linear response function ⟨⟨*H*_*k*_ *H*_*l*_⟩⟩ as defined in eqs 9 and 12 can be expanded in powers of β = 1/*k*_B_ *T*. If such a series is truncated at finite order, this corresponds to a high-temperature approximation. The higher the number of retained terms, the more the validity is extended to lower temperatures. The Taylor series of ⟨⟨*H*_*k*_ *H*_*l*_⟩⟩ around β = 0 is given by

13 $$\left\langle \left\langle H_{k}H_{l}\right\rangle \right\rangle =\langle {\left\langle H_{k}H_{l}\right\rangle \rangle }_{\beta =0}+{\left(\frac{\partial \left\langle \left\langle H_{k}H_{l}\right\rangle \right\rangle }{\partial \beta }\right)}_{\beta =0}\beta +\frac{1}{2!}{\left(\frac{\partial^{2}\left\langle \left\langle H_{k}H_{l}\right\rangle \right\rangle }{\partial \beta^{2}}\right)}_{\beta =0}\beta^{2}+\frac{1}{3!}{\left(\frac{\partial^{3}\left\langle \left\langle H_{k}H_{l}\right\rangle \right\rangle }{\partial \beta^{3}}\right)}_{\beta =0}\beta^{3}+\cdot \cdot \cdot$$

i.e., the coefficients in the expansion are derivatives with respect to β. Truncating the expansion at the linear term gives Curie’s law for the magnetic susceptibility and truncating at the second-order term leads to Bleaney’s theory. The main theoretical result of this work is the derivation of analytical equations for the third-order derivative, i.e., one order beyond the second-order Bleaney’s theory.

Starting with the operator equation for the dyadic (eq 9), we derive in Appendix A model-agnostic expressions for the first three derivatives of ⟨⟨*H*_*k*_ *H*_*l*_⟩⟩. (Model-agnostic means that these equations are, for instance, also valid in an *ab initio* context and do not require a specific form for the Hamiltonian). From now on, we assume that our field-free Hamiltonian is parametrized according to eq 2. Furthermore, we assume that the derivatives are evaluated at zero field. Since in this case the dimension of the Hilbert space is |H|=2J+1

14 $$\langle {\left\langle H_{k}H_{l}\right\rangle \rangle }_{\beta =0}=0$$

15 $${\left(\frac{\partial \left\langle \left\langle H_{k}H_{l}\right\rangle \right\rangle }{\partial \beta }\right)}_{\beta =0}=-\frac{1}{2J+1}\mathrm{tr}\left(H_{k}H_{l}\right)$$

16 $${\left(\frac{\partial^{2}\left\langle \left\langle H_{k}H_{l}\right\rangle \right\rangle }{\partial \beta^{2}}\right)}_{\beta =0}=\frac{1}{2J+1}\mathrm{tr}\left[\left(H_{k}H_{l}+H_{l}H_{k}\right)H^{\left(0\right)}\right]$$

17 $${\left(\frac{\partial^{3}\left\langle \left\langle H_{k}H_{l}\right\rangle \right\rangle }{\partial \beta^{3}}\right)}_{\beta =0}=3\frac{\mathrm{tr}\left({\left(H^{\left(0\right)}\right)}^{2}\right)}{{\left(2J+1\right)}^{2}}\mathrm{tr}\left(H_{k}H_{l}\right)-\frac{1}{2J+1}\mathrm{tr}\left[\left(H_{k}H_{l}+H_{l}H_{k}\right){\left(H^{\left(0\right)}\right)}^{2}\right]-\frac{1}{2J+1}\mathrm{tr}\left(H_{k}H^{\left(0\right)}H_{l}H^{\left(0\right)}\right)$$

As one can see (using the invariance of the trace under circular shifts for the last term), the dyadic is symmetric under interchange of *k* and *l*. This is also expected from the infinite-order expression eq 9. Hence, the 3 × 3 matrix ⟨⟨**JJ** ^T^⟩⟩ containing the ⟨⟨*J*_*k*_ *J*_*l*_⟩⟩ as elements is a symmetric tensor.

For a vector (rank-1 tensor) **v**, one can define “spherical” components via

18 $$\begin{array}{l} v_{0}^{\left(1\right)}=v_{z}\\ v_{\pm 1}^{\left(1\right)}=\mp \frac{1}{\sqrt{2}}\left(v_{x}\pm iv_{y}\right) \end{array}$$

This can also be written as

19 $$v_{m}^{\left(1\right)}=\sum_{i=x,y,z}A_{\mathrm{mi}}v_{i}$$

with the unitary transformation matrix from Cartesian to spherical components given by

20 $$A=\left(\begin{array}{lll} -1/\sqrt{2} & -i/\sqrt{2} & 0\\ 0 & 0 & 1\\ 1/\sqrt{2} & -i/\sqrt{2} & 0 \end{array}\right)$$

The rows of **A** encode from top to bottom the *m* = +1, *m* = 0 and *m* = −1 spherical components of the vector. Any symmetric 3 × 3 Cartesian tensor **T** can also be described through spherical tensor components of rank 0 and 2. These are defined by first transforming each of the Cartesian indices of the tensor to a spherical index using the matrix **A** just introduced, and afterward contracting these two indices to a total angular momentum of *K* = 0 or *K* = 2 (*K* = 1 vanishes for symmetric tensors) using CGCs

21 $$T_{Q}^{\left(K\right)}=\sum_{\mathrm{qq}\prime \mathrm{ij}}\left(\begin{array}{ll} 1 & 1\\ q & q^{\prime } \end{array}|\begin{array}{l} K\\ Q \end{array}\right)A_{\mathrm{qi}}A_{q\prime j}T_{\mathrm{ij}}$$

More explicitly, the spherical components are

22 $$\begin{array}{l} T_{0}^{\left(0\right)}=-\frac{1}{\sqrt{3}}\left(T_{\mathrm{xx}}+T_{\mathrm{yy}}+T_{\mathrm{zz}}\right)\\ T_{2}^{\left(2\right)}=\frac{1}{2}\left(T_{\mathrm{xx}}-T_{\mathrm{yy}}+2iT_{\mathrm{xy}}\right)\\ T_{1}^{\left(2\right)}=-T_{\mathrm{xz}}-iT_{\mathrm{yz}}\\ T_{0}^{\left(2\right)}=\frac{1}{\sqrt{6}}\left(2T_{\mathrm{zz}}-T_{\mathrm{xx}}-T_{\mathrm{yy}}\right)\\ T_{-1}^{\left(2\right)}=T_{\mathrm{xz}}-iT_{\mathrm{yz}}\\ T_{-2}^{\left(2\right)}=\frac{1}{2}\left(T_{\mathrm{xx}}-T_{\mathrm{yy}}-2iT_{\mathrm{xy}}\right) \end{array}$$

This is a linear transformation. The inverse transformation is given by

23 $$\begin{array}{l} T_{\mathrm{xx}}=-\frac{1}{\sqrt{3}}T_{0}^{\left(0\right)}+\frac{1}{2}\left(T_{2}^{\left(2\right)}+T_{-2}^{\left(2\right)}\right)-\frac{1}{\sqrt{6}}T_{0}^{\left(2\right)}\\ T_{\mathrm{yy}}=-\frac{1}{\sqrt{3}}T_{0}^{\left(0\right)}-\frac{1}{2}\left(T_{2}^{\left(2\right)}+T_{-2}^{\left(2\right)}\right)-\frac{1}{\sqrt{6}}T_{0}^{\left(2\right)}\\ T_{\mathrm{zz}}=-\frac{1}{\sqrt{3}}T_{0}^{\left(0\right)}+\frac{2}{\sqrt{6}}T_{0}^{\left(2\right)}\\ T_{\mathrm{xy}}=T_{\mathrm{yx}}=-\frac{i}{2}\left(T_{2}^{\left(2\right)}-T_{-2}^{\left(2\right)}\right)\\ T_{\mathrm{xz}}=T_{\mathrm{zx}}=-\frac{1}{2}\left(T_{1}^{\left(2\right)}-T_{-1}^{\left(2\right)}\right)\\ T_{\mathrm{yz}}=T_{\mathrm{zy}}=\frac{i}{2}\left(T_{1}^{\left(2\right)}+T_{-1}^{\left(2\right)}\right) \end{array}$$

Using the definition eq 21 and the linearity of ⟨⟨*H*_*k*_ *H*_*l*_⟩⟩ in both operators *H*_*k*_ and *H*_*l*_, the spherical tensor components of the angular momentum dyadic can be written as

24 $$\langle {\left\langle {\mathrm{JJ}}^{T}\rangle \right\rangle }_{Q}^{\left(K\right)}=\sum_{\mathrm{qq}\prime }\left(\begin{array}{ll} 1 & 1\\ q & q^{\prime } \end{array}|\begin{array}{l} K\\ Q \end{array}\right)\left\langle \left\langle J_{q}^{\left(1\right)}J_{q\prime }^{\left(1\right)}\right\rangle \right\rangle$$

In Appendix B, we derive equations for derivatives of the spherical components of the angular momentum dyadic with respect to β = 1/*k*_B_ *T*, which can then be back-transformed to Cartesian components using eqs 23. In this derivation, we made use of graphical techniques for evaluating contractions of CGCs and antisymmetric symbols.^35,36^ The resulting equations are

25 $${\left(\frac{\partial \langle {\left\langle {\mathrm{JJ}}^{T}\rangle \right\rangle }_{Q}^{\left(K\right)}}{\partial \beta }\right)}_{\beta =0}=\frac{1}{\sqrt{3}}J\left(J+1\right)\delta_{K0}\delta_{Q0}$$

26 $${\left(\frac{\partial^{2}\langle {\left\langle {\mathrm{JJ}}^{T}\rangle \right\rangle }_{Q}^{\left(2\right)}}{\partial \beta^{2}}\right)}_{\beta =0}=\frac{1}{5\sqrt{6}}J\left(J+1\right)\left(2J-1\right)\left(2J+3\right)B_{Q}^{\left(2\right)}$$

$${\left(\frac{\partial^{3}\langle {\left\langle {\mathrm{JJ}}^{T}\rangle \right\rangle }_{0}^{\left(0\right)}}{\partial \beta^{3}}\right)}_{\beta =0}=-\frac{1}{2\sqrt{3}}\underset{k=2,\cdot \cdot \cdot ,2J}{\sum }\frac{k\left(k+1\right)}{\sqrt{2k+1}}\langle J∥O^{\left(k\right)}∥J\rangle^{2}{\left(B^{\left(k\right)}⊗B^{\left(k\right)}\right)}_{0}^{\left(0\right)}$$ 27

$$\begin{array}{l} {\left(\frac{\partial^{3}\langle {\left\langle {\mathrm{JJ}}^{T}\rangle \right\rangle }_{Q}^{\left(2\right)}}{\partial \beta^{3}}\right)}_{\beta =0}=\sqrt{\frac{2}{15}}\underset{k=2,\cdot \cdot \cdot ,2J}{\sum }\langle J∥O^{\left(k\right)}∥J\rangle^{2}k\left(k+1\right)\sqrt{\frac{\left(2k-2\right)!}{\left(2k+3\right)!}}\left[6J\left(J+1\right)-\frac{5}{2}k\left(k+1\right)+3\right]{\left(B^{\left(k\right)}⊗B^{\left(k\right)}\right)}_{Q}^{\left(2\right)}-\frac{6}{\sqrt{5}}\underset{k=4,\cdot \cdot \cdot ,2J}{\sum }\langle J∥O^{\left(k\right)}∥J\rangle^{2}\sqrt{\frac{k\left(k-1\right)}{\left(2k+1\right)\left(2k-1\right)\left(2k-3\right)}}{\left(B^{\left(k\right)}⊗B^{\left(k-2\right)}\right)}_{Q}^{\left(2\right)} \end{array}$$ 28

In the last two equations, (*B* ^(*k*)^ ⊗ *B* ^(*k̃*)^)_*Q*_^(*K*)^ is defined as

$${\left(B^{\left(k\right)}{⊗B}^{\left(\mathrm{k̃}\right)}\right)}_{Q}^{\left(K\right)}=\underset{q\mathrm{q̃}}{\sum }\left(\begin{array}{ll} k & \mathrm{k̃}\\ q & \mathrm{q̃} \end{array}|\begin{array}{l} K\\ Q \end{array}\right)B_{q}^{\left(k\right)}B_{\mathrm{q̃}}^{\left(\mathrm{k̃}\right)}$$ 29

One can see that the first derivative (linear part) of the *K* = 2 component of the spin dyadic is zero. Hence, the linear part is completely isotropic. The *K* = 0 (isotropic) part of the second derivative vanishes because it is proportional to *B*_0_^(0)^, which is zero for a traceless Hamiltonian. Equation 26 gives the only contribution to the *anisotropy* of the dyadic up to order β^2^ (since the order β contribution is completely isotropic). This is our version of Bleaney’s equations. While Bleaney formulated his theory explicitly for the susceptibility tensor, we have here a more general setting, focusing on the angular momentum dyadic (which for Ln complexes differs from the susceptibility by a constant factor as shown in eq 6). Furthermore, Bleaney’s equations are normally expressed in terms of ligand-field parameters in the Stevens operator formalism, while we use a parametrization in terms of BST operators. As we will show below, our formulation is also applicable to TM complexes and beyond the point-dipole approximation for the hyperfine interaction. Finally, while Bleaney’s equations directly give the two anisotropy parameters of the susceptibility tensor, and therefore are only valid in the principal axis frame, our version of Bleaney’s theory is valid in any coordinate frame and gives the complete anisotropic part of the angular momentum dyadic (respectively susceptibility tensor). Importantly, higher-order ligand-field parameters are not neglected in Bleaney’s theory: they simply do not contribute at order 1/*T* ^2^.

A special case of eq 28 was already derived by McGarvey^19^ (eqs 29 and 30 of his article). However, McGarvey assumed highly symmetric systems for which one knows the principal axis frame of the susceptibility tensor a priori. In low-symmetry situations, our equation, which is valid in any coordinate frame, is clearly superior. It has the additional advantage that summations over *k*, *q*, and *q̃* are, in contrast to McGarvey’s equations, not explicit. As a result, our equation fits into two lines in contrast to McGarvey’s equations, which required 28 lines in total.

### 2.3 Lanthanide Complexes

For Ln complexes, the field-free Hamiltonian is most commonly parametrized in the (extended) Stevens operator formalism^2,37^ or using the Wybourne formalism,^38^ of which there exists a real (tesseral tensor operator, TTO) and complex (STO) flavor. The different notations for parameters used in this section are summarized in Table 1.

**Table 1:** Different Notations for Ligand-Field Parameters Used in This Section

symbol	meaning
*B*_*q*_^(*k*)^	Generic parameter introduced via eq 2 (can be used for both ZFS and ligand field)
*B*_*kq*_	Complex Wybourne parameter
*B*_*kq*_^real^	Real Wybourne parameter
*B*_*k*_^*q*^	Stevens parameter

For Ln complexes, using first-order degenerate perturbation theory within the lowest *J*-manifold to account for the effect of the ligand field, one can justify that only terms up to *k* = 6 should be included in eq 2. Exceptions are the few cases in which *J* is smaller than 3. For example, cerium­(III) and samarium­(III) both have *J* = 5/2, which means that only *k* = 2 and *k* = 4 are needed to parametrize the ligand field. However, one should note that samarium­(III) also has a low-energy excited *J*-manifold that must be considered for a proper description of its magnetic properties,^1^ i.e., eqs 1 and 2 should not be used in this case.

The most widespread parametrization of the field-free effective Hamiltonian of Ln complexes within their lowest *J*-manifold makes use of the (extended) Stevens operators *O*_*k*_^*q*^

30 $$H^{\left(0\right)}=\sum_{k}\sum_{q=-k}^{k}\theta_{k}B_{k}^{q}O_{k}^{q}$$

This equation is derived under two approximations: that SOC does not couple different *LS* terms and that the ligand field does not couple different *J*-manifolds. In other words, it is assumed that *L*, *S*, *J* are all good quantum numbers and that one can employ first-order degenerate perturbation theory (within the *LS* term and within the *J*-manifold) to account for the effects of SOC and the ligand field. If these assumptions are satisfied, the *B*_*k*_^*q*^ are identical to the one-electron ligand-field parameters. However, the model is more generally valid and deviations from the assumptions can be captured by effective *B*_*k*_^*q*^ parameters that deviate from the one-electron ones.^39,40^ The Stevens operators *O*_*k*_^*q*^ are the equivalent operators of certain polynomials *P*_*k*_^*q*^ in the components of the unit vector (*x̂*, *ŷ*, *ẑ*) = (*x*, *y*, *z*)/*r* that are equal to unnormalized tesseral harmonics. “Equivalent operator” means that *x̂*, *ŷ*, *ẑ* in the polynomial are replaced by the operators *J*_*x*_, *J*_*y*_, *J*_*z*_, additionally symmetrizing the result to take into account that the angular momentum operators do not commute. For example, the Stevens operators *O*_2_^0^ = 3*J*_*z*_^2^ – **J** ^2^ = 3*J*_*z*_^2^ – *J*(*J* + 1) and *O*_2_^2^ = *J*_*x*_^2^ – *J*_*y*_^2^ are the equivalent operators of the polynomials *P*_2_^0^ = 2*ẑ* ^2^ – *x̂* ^2^ – *ŷ* ^2^ = 3*ẑ* ^2^ – 1 and *P*_2_^2^ = *x̂* ^2^ – *ŷ* ^2^, respectively. Within a single *J*-manifold (usually the lowest one), there is an equivalence (in the sense of having identical matrix elements) of the polynomial (interpreted as a one-electron operator) and a multiple of the equivalent operator

31 $$\sum_{i=1}^{N_{f}}P_{k}^{q}\left(i\right)\equiv \theta_{k}O_{k}^{q}$$

The numerical factor θ_*k*_ (which apart from the Ln ion only depends on *k* and not on *q*) is called an equivalence coefficient.^41^ The θ_*k*_ have been tabulated for all Ln ions.^41^

The BST operators *O*_*q*_^(*k*)^ that we employ in eq 2 are the equivalent operators of scaled spherical harmonics

32 $$\sum_{i=1}^{N_{f}}C_{q}^{\left(k\right)}\left(i\right)=\sum_{i=1}^{N_{f}}\sqrt{\frac{4\pi }{2k+1}}Y_{\mathrm{kq}}\left(i\right)\equiv \theta_{k}O_{q}^{\left(k\right)}$$

Here, *Y*_*kq*_ are the usual (complex) spherical harmonics, which can also be written as polynomials in *x̂*, *ŷ*, *ẑ*. For us, the explicit form of the *O*_*q*_^(*k*)^ operators as polynomials in *J*_*x*_, *J*_*y*_, *J*_*z*_ is not important. Instead, we work exclusively with the Wigner–Eckart theorem, eqs 4 and 5, which can also serve as the *definition* of these operators.

The real spherical harmonics (or tesseral harmonics) $Y_{\mathrm{kq}}$ are defined as

33 $$Y_{k0}=Y_{k0}$$

and, for *q* > 0

34 $$Y_{\mathrm{kq}}=\frac{1}{\sqrt{2}}\left[Y_{k,-q}+{\left(-1\right)}^{q}Y_{\mathrm{kq}}\right]$$

35 $$Y_{k,-q}=\frac{i}{\sqrt{2}}\left[Y_{k,-q}-{\left(-1\right)}^{q}Y_{\mathrm{kq}}\right]$$

In spherical coordinates, the $Y_{\mathrm{kq}}$ are proportional to cos­(|*q*|ϕ) and the $Y_{k,-q}$ are proportional to sin­(|*q*|ϕ), where ϕ is the azimuthal angle. This is why they are also called the “cosine” and “sine” tesseral harmonics. Similarly, one can define the TTO versions of the BST operators

36 $$O_{k0}^{\mathrm{TTO}}=O_{0}^{\left(k\right)}$$

and, for *q* > 0 (leaving away the 1/√2 normalization constant)

37 $$O_{\mathrm{kq}}^{\mathrm{TTO}}=O_{-q}^{\left(k\right)}+{\left(-1\right)}^{q}O_{q}^{\left(k\right)}$$

38 $$O_{k,-q}^{\mathrm{TTO}}=i\left[O_{-q}^{\left(k\right)}-{\left(-1\right)}^{q}O_{q}^{\left(k\right)}\right]$$

The latter equation can also be written as

39 $$O_{\mathrm{kq}}^{\mathrm{TTO}}=i\left[O_{q}^{\left(k\right)}-{\left(-1\right)}^{q}O_{-q}^{\left(k\right)}\right],\mathrm{if}\;q<0$$

Note that, since the 1/√2 normalization constant is left out, *O*_*kq*_^TTO^ for *q* ≠ 0 is the equivalent operator of $\sqrt{8\pi /\left(2k+1\right)}Y_{\mathrm{kq}}$ .

Again making the same approximations as above for the Stevens parametrization (i.e., assuming that *L*, *S*, *J* are good quantum numbers), the field-free Hamiltonian can be expressed in terms of either of those sets of operators

40 $$H^{\left(0\right)}=\sum_{k}\sum_{q=-k}^{k}\theta_{k}B_{\mathrm{kq}}^{\mathrm{real}}O_{\mathrm{kq}}^{\mathrm{TTO}}$$

41 $$H^{\left(0\right)}=\sum_{k}\sum_{q=-k}^{k}\theta_{k}B_{\mathrm{kq}}O_{q}^{\left(k\right)}$$

The first of these two equations corresponds to the [ST−] convention of reference 42. These are the real and complex Wybourne parametrizations for the field-free Hamiltonian. Note that in eq 41, always the same *q* occurs in the operator and in the parameter *B*_*kq*_ multiplying it. This is in contrast to our parametrization eq 2, where the “magnetic quantum numbers” on the operator and the parameter are opposite. Therefore, the parameters *B*_*kq*_ are not the *q*-components of spherical tensors. Instead, to work with proper spherical tensors, one has to define (compare eqs 41 and 2) *B*_*q*_^(*k*)^ = θ_*k*_(*B*_*kq*_)*.

In general, the parameters in the field-free Hamiltonian are real-valued in the case of the *O*_*kq*_^TTO^ and complex-valued in the case of the *O*_*q*_^(*k*)^ operators. One can relate the two sets of parameters by equating the two equations. Starting from the Hamiltonian in terms of TTOs, one obtains

42 H(0)=∑kθk∑q=−kkBkqrealOkqTTO=∑kθk[Bk0realO0(k)+∑q=1kBkqreal[O−q(k)+(−1)qOq(k)]+∑q=−k−1Bkqreali[Oq(k)−(−1)qO−q(k)]]=∑kθk[Bk0realO0(k)+∑q=1k(−1)q[Bkqreal−iBk,−qreal]Oq(k)+∑q=−k−1[Bk,−qreal+iBkqreal]Oq(k)]=∑kθk∑q=−kkBkqOq(k)

This shows that

43 $$B_{k0}=B_{k0}^{\mathrm{real}}$$

and, for *q* > 0

44 $$B_{\mathrm{kq}}={\left(-1\right)}^{q}\left[B_{\mathrm{kq}}^{\mathrm{real}}-iB_{k,-q}^{\mathrm{real}}\right]$$

45 $$B_{k,-q}=B_{\mathrm{kq}}^{\mathrm{real}}+iB_{k,-q}^{\mathrm{real}}$$

These equations show that the real-valued *B*_*kq*_^real^ parameters are not just the real and imaginary part of the complex *B*_*kq*_ parameters as is sometimes incorrectly stated.^43^ Instead, for *q* > 0, $B_{\mathrm{kq}}^{\mathrm{real}}=RB_{k,-q}$ and $B_{k,-q}^{\mathrm{real}}=IB_{k,-q}$ . The (extended) Stevens operators differ from the *O*_*kq*_^TTO^ by numerical factors. There is a one-to-one relation between the parameters in the two tesseral tensor operator formalisms, *B*_*k*_^*q*^ = λ_*kq*_ *B*_*kq*_^real^, where the proportionality constants λ_*kq*_ are given in Table 2.

**Table 2:** Table of λ_*kq*_ Values for Converting between Stevens and Real Wybourne Parameters^43,44^

		|*q*|						
		0	1	2	3	4	5	6
	2	$\frac{1}{2}$	√6	$\frac{1}{2}\sqrt{6}$				
*k*	4	$\frac{1}{8}$	$\frac{1}{2}\sqrt{5}$	$\frac{1}{4}\sqrt{10}$	$\frac{1}{2}\sqrt{35}$	$\frac{1}{8}\sqrt{70}$		
	6	$\frac{1}{16}$	$\frac{1}{8}\sqrt{42}$	$\frac{1}{16}\sqrt{105}$	$\frac{1}{8}\sqrt{105}$	$\frac{3}{16}\sqrt{14}$	$\frac{3}{8}\sqrt{77}$	$\frac{1}{16}\sqrt{231}$

The conventional version of Bleaney’s theory is formulated in terms of Stevens parameters. In order to derive it from our more general version, we first combine eqs 7 and 26 to pass from the dyadic to the susceptibility

46 $$\chi_{q}^{\left(2\right)}=-\frac{\mu_{0}\mu_{B}^{2}g_{J}^{2}J\left(J+1\right)\left(2J-1\right)\left(2J+3\right)\beta^{2}}{10\sqrt{6}}B_{q}^{\left(2\right)}$$

The axial and rhombic susceptibility anisotropy parameters are defined in the eigenframe of the susceptibility tensor as follows

47 $$\chi_{\mathrm{ax}}=\chi_{\mathrm{zz}}-\frac{1}{2}\left(\chi_{\mathrm{xx}}+\chi_{\mathrm{yy}}\right)=\sqrt{\frac{3}{2}}\chi_{0}^{\left(2\right)}=-\frac{\mu_{0}\mu_{B}^{2}g_{J}^{2}J\left(J+1\right)\left(2J-1\right)\left(2J+3\right)\beta^{2}}{20}B_{0}^{\left(2\right)}$$

48 $$\chi_{\mathrm{rh}}=\frac{1}{2}\left(\chi_{\mathrm{xx}}-\chi_{\mathrm{yy}}\right)=\frac{1}{2}\left(\chi_{2}^{\left(2\right)}+\chi_{-2}^{\left(2\right)}\right)=-\frac{\mu_{0}\mu_{B}^{2}g_{J}^{2}J\left(J+1\right)\left(2J-1\right)\left(2J+3\right)\beta^{2}}{20\sqrt{6}}\left(B_{2}^{\left(2\right)}+B_{-2}^{\left(2\right)}\right)$$

We can now step-by-step pass from our parametrization to the Stevens parametrization:

49 $$B_{0}^{\left(2\right)}=\theta_{2}{\left(B_{20}\right)}^{*}=\theta_{2}B_{20}^{\mathrm{real}}=\theta_{2}B_{2}^{0}/\lambda_{20}=2\theta_{2}B_{2}^{0}$$

50 $$B_{2}^{\left(2\right)}+B_{-2}^{\left(2\right)}=\theta_{2}{\left(B_{22}+B_{2,-2}\right)}^{*}=2\theta_{2}B_{22}^{\mathrm{real}}=2\theta_{2}B_{2}^{2}/\lambda_{22}=4\theta_{2}B_{2}^{2}/\sqrt{6}$$

Inserting these identities into eqs 47 and 48 and defining the “Bleaney coefficients” as

51 $$C_{J}=\theta_{2}J\left(J+1\right)\left(2J-1\right)\left(2J+3\right)g_{J}^{2}$$

leads to the common version of Bleaney’s theory^41^

52 $$\begin{array}{l} \chi_{\mathrm{ax}}=-\frac{\mu_{0}\mu_{B}^{2}C_{J}}{10{\left(k_{B}T\right)}^{2}}B_{2}^{0}\\ \chi_{\mathrm{rh}}=-\frac{\mu_{0}\mu_{B}^{2}C_{J}}{30{\left(k_{B}T\right)}^{2}}B_{2}^{2} \end{array}$$

We did not derive the equivalent of our eq 28 (1/*T* ^3^ term) in terms of Stevens parameters. Instead, if a parametrization of the ligand field of a Ln complex is available in terms of Stevens parameters, we find it more convenient to convert these into our *B*_*q*_^(*k*)^ parameters and evaluate eq 28 using our convention.

### 2.4 Transition-Metal Complexes

For TM complexes, the field-free spin Hamiltonian is most commonly expanded in terms of the Cartesian spin operators and their products. The most widespread parametrization is in terms of the so-called zero-field splitting (ZFS) tensor **D**

53 $$H^{\left(0\right)}=S^{T}\mathrm{DS}$$

However, note that this form is not sufficient for all cases. For example, it cannot correctly describe the ZFS in high-spin iron­(III) complexes in a tetrahedral ligand field, where double group considerations show that the ^6^ *A*_1_ term is split into two levels that are 2-fold and 4-fold degenerate. The simple **S** ^T^ **DS** Hamiltonian vanishes in molecules that have more than one higher-order symmetry axis.

**D** is a symmetric tensor. Without loss of generality, one can also assume that it is traceless, which we will assume from here on. The reason is that the trace of the **D**-tensor only corresponds to an overall constant shift of all energy levels, which does not change observable properties. Since **D** is a symmetric tensor, there exists an orthonormal coordinate frame in which it becomes diagonal, with diagonal elements *D*_*x*_, *D*_*y*_, and *D*_*z*_. The most common convention for labeling these three components is |*D*_*x*_| ≤ |*D*_*y*_| ≤ |*D*_*z*_|. Since the tensor is traceless, the diagonal elements can be expressed in terms of only two parameters, which are the *axial* ZFS parameter

54 $$D=D_{z}-\frac{1}{2}\left(D_{x}+D_{y}\right)$$

and the *rhombic* ZFS parameter

55 $$E=\frac{1}{2}\left(D_{x}-D_{y}\right)$$

*E* is also often expressed in terms of the ratio *E*/*D*, called *rhombicity*. If **D** is traceless, which we assume here, the axial parameter can be written as $D=\frac{3}{2}D_{z}$ and the rhombicity can be expressed as

56 $$\frac{E}{D}=\frac{1}{3}\frac{D_{x}-D_{y}}{D_{z}}=\frac{1}{3}\frac{\pm \left(\left|D_{y}\right|-\left|D_{x}\right|\right)}{\pm \left|D_{z}\right|}=\frac{1}{3}\frac{\left|D_{y}\right|-\left|D_{x}\right|}{\left|D_{z}\right|}$$

From this equation one can conclude that the rhombicity *E*/*D* can only take values between 0 (the “axial” case, where *D*_*x*_ = *D*_*y*_) and 1/3 (the fully “rhombic” case, where *D*_*x*_ = 0 and *D*_*y*_ = −*D*_*z*_). In its eigenframe, the **D** tensor can be expressed in terms of these parameters as

57 $$D=D\left(\begin{array}{lll} -\frac{1}{3}+\frac{E}{D} & 0 & 0\\ 0 & -\frac{1}{3}-\frac{E}{D} & 0\\ 0 & 0 & \frac{2}{3} \end{array}\right)$$

In order to adapt our general equations for the derivatives of the angular momentum dyadic (eqs 25–28) to the TM case, we can first recognize that eq 2 (including only a *k* = 2 term) becomes equal to the ZFS Hamiltonian eq 53 if we set

58 $$B_{q}^{\left(k\right)}=\sqrt{\frac{2}{3}}D_{q}^{\left(k\right)}$$

Here, *D*_*q*_^(*k*)^ are the spherical components of the **D**-tensor according to eqs 21 and 22. Our assumption *B*_0_^(0)^ = 0 is fulfilled because we assumed the **D**-tensor to be traceless. All *B*_*q*_^(*k*)^ with *k* > 2 vanish. The first derivative of the dyadic, eq 25, is given by

59 $${\left(\frac{\partial \langle {\left\langle {\mathrm{SS}}^{T}\rangle \right\rangle }_{0}^{\left(0\right)}}{\partial \beta }\right)}_{\beta =0}=\frac{1}{\sqrt{3}}S\left(S+1\right)$$

and can be transformed back into Cartesian form using eqs 23, which gives

60 $${\left(\frac{\partial \left\langle \left\langle {\mathrm{SS}}^{T}\right\rangle \right\rangle }{\partial \beta }\right)}_{\beta =0}=-\frac{S\left(S+1\right)}{3}I$$

Here, **I** is the 3 × 3 unit matrix. The second derivative of the dyadic, eq 26, is given by

61 $${\left(\frac{\partial^{2}\langle {\left\langle {\mathrm{SS}}^{T}\rangle \right\rangle }_{Q}^{\left(2\right)}}{\partial \beta^{2}}\right)}_{\beta =0}=\frac{1}{15}S\left(S+1\right)\left(2S-1\right)\left(2S+3\right)D_{Q}^{\left(2\right)}$$

which has the Cartesian form

62 $${\left(\frac{\partial^{2}\left\langle \left\langle {\mathrm{SS}}^{T}\right\rangle \right\rangle }{\partial \beta^{2}}\right)}_{\beta =0}=\frac{1}{15}S\left(S+1\right)\left(2S-1\right)\left(2S+3\right)D$$

The isotropic part of the third derivative, eq 27, is given by

$${\left(\frac{\partial^{3}\langle {\left\langle {\mathrm{SS}}^{T}\rangle \right\rangle }_{0}^{\left(0\right)}}{\partial \beta^{3}}\right)}_{\beta =0}=-\frac{1}{2\sqrt{15}}S\left(S+1\right)\left(2S-1\right)\left(2S+3\right){\left(D^{\left(2\right)}⊗D^{\left(2\right)}\right)}_{0}^{\left(0\right)}$$ 63

One can show (see our Python script in the Supporting Information) that (*D* ^(2)^ ⊗ *D* ^(2)^)_0_^(0)^ corresponds to the Cartesian tensor $-\frac{1}{\sqrt{15}}\mathrm{tr}\left(D^{2}\right)I$ . This means that the isotropic part of the third derivative in Cartesian form is given by

64 $${\left(\frac{\partial^{3}\left\langle \left\langle {\mathrm{SS}}^{T}\right\rangle \right\rangle }{\partial \beta^{3}}\right)}_{\beta =0}^{\mathrm{iso}}=\frac{1}{30}S\left(S+1\right)\left(2S-1\right)\left(2S+3\right)\mathrm{tr}\left(D^{2}\right)I$$

The anisotropic part of the third derivative, eq 28, is given by

65 $${\left(\frac{\partial^{3}\langle {\left\langle {\mathrm{SS}}^{T}\rangle \right\rangle }_{Q}^{\left(2\right)}}{\partial \beta^{3}}\right)}_{\beta =0}=\frac{1}{5\sqrt{21}}S\left(S+1\right)\left(2S-1\right)\left(2S+3\right)\left(S+2\right)\left(S-1\right){\left(D^{\left(2\right)}⊗D^{\left(2\right)}\right)}_{Q}^{\left(2\right)}$$

One can show that (*D* ^(2)^ ⊗ *D* ^(2)^)_*Q*_^(2)^ corresponds to the Cartesian tensor $-2\sqrt{\frac{3}{7}}{\left(D^{2}\right)}^{\mathrm{aniso}}$ , where for a general Cartesian 3 × 3 tensor **T** we define the anisotropic part as $T^{\mathrm{aniso}}=T-\frac{1}{3}\mathrm{tr}\left(T\right)I$ . This means that the anisotropic part of the third derivative in Cartesian form is given by

66 $${\left(\frac{\partial^{3}\left\langle \left\langle {\mathrm{SS}}^{T}\right\rangle \right\rangle }{\partial \beta^{3}}\right)}_{\beta =0}^{\mathrm{aniso}}=-\frac{2}{35}S\left(S+1\right)\left(2S-1\right)\left(2S+3\right)\left(S+2\right)\left(S-1\right){\left(D^{2}\right)}^{\mathrm{aniso}}$$

Using a Taylor expansion up to order β^3^, we can combine the different derivatives to approximate the spin dyadic as

67 $$\left\langle \left\langle {\mathrm{SS}}^{T}\right\rangle \right\rangle \approx -\frac{S\left(S+1\right)}{3}I\beta +\frac{1}{30}S\left(S+1\right)\left(2S-1\right)\left(2S+3\right)D\beta^{2}+\frac{1}{15}S\left(S+1\right)\left(2S-1\right)\left(2S+3\right)\left[\frac{1}{12}\mathrm{tr}\left(D^{2}\right)I-\frac{1}{7}\left(S+2\right)\left(S-1\right){\left(D^{2}\right)}^{\mathrm{aniso}}\right]\beta^{3}$$

Upon inserting this expansion into eq 7 for the susceptibility tensor, one straightforwardly obtains Stout and Gutowsky’s^17^ eq 4. Also common equations for the inverse-temperature expansion of the susceptibility tensor in the case of an anisotropic **g**-tensor^29,45^ can be obtained straightforwardly by inserting eq 67 into eq 6.

An advantage of our formalism based on the (spin) angular momentum dyadic is that it also allows to express the temperature dependence of the chemical shielding tensor beyond the point-dipole approximation that was assumed by Bleaney.^1^ By inserting eq 67 into eq 8, we straightforwardly obtain (see the Supporting Information Section S2) the following equations for the contact (c) and pseudocontact (pc) contribution to isotropic hyperfine shieldings in the case of an axially symmetric molecule

68 $$\sigma_{\mathrm{iso}}^{c}=-\frac{\mu_{B}A_{\mathrm{iso}}}{3\gamma }\left\{S\left(S+1\right)\beta g_{\mathrm{iso}}-D\beta^{2}\frac{S\left(S+1\right)\left(2S-1\right)\left(2S+3\right)}{15}\Delta g-D^{2}\beta^{3}\frac{S\left(S+1\right)\left(2S-1\right)\left(2S+3\right)}{45}\left[\frac{1}{2}g_{\mathrm{iso}}-\frac{2\left(S+2\right)\left(S-1\right)}{7}\Delta g\right]\right\}$$

69 $$\sigma_{\mathrm{iso}}^{\mathrm{pc}}=-\frac{\mu_{B}\Delta A}{3\gamma }\left\{S\left(S+1\right)\beta 2\Delta g-D\beta^{2}\frac{S\left(S+1\right)\left(2S-1\right)\left(2S+3\right)}{15}\left(g_{\mathrm{iso}}+\Delta g\right)-D^{2}\beta^{3}\frac{S\left(S+1\right)\left(2S-1\right)\left(2S+3\right)}{45}\left[\Delta g-\frac{2\left(S+2\right)\left(S-1\right)}{7}\left(g_{\mathrm{iso}}+\Delta g\right)\right]\right\}$$

These two equations unify the separate equations that Martin and Autschbach derived for each individual spin quantum number *S*,^28^ replacing them with a single set of equations valid for any *S*. Notably, no diagonalization of the field-free Hamiltonian (as used by them) is necessary to arrive at these equations. Furthermore, having a single set of equations that is valid for any *S* is less error-prone. For example, we could still identify a few small mistakes in their equations that were not corrected in the erratum:^46^ their corrected eq 23e lacks a factor of 1/2 in the *T* ^–3^ term and their corrected eq 23f has the wrong sign for the *T* ^–2^ term. Finally, their eq 23h has the wrong sign for the *T* ^–3^ term. The general conclusions of their paper and the erratum are not affected by these small mistakes.

## 3 Results

All results reported below were obtained with our Julia package ParaMag.jl,^34,47^ in which we implemented the analytical dyadic up to third order according to eq 13 with eqs 25–28. For transition-metal complexes with ZFS Hamiltonian **S** ^T^ **DS**, these equations are equivalent to eq 67.

### 3.1 Test Sets

We tested the convergence of the expansion of the angular momentum dyadic for both TM and Ln complexes. For an approximation **M** ^approx^ to a matrix **M** ^exact^ (**M** can for example be the complete dyadic or one of its derivatives with respect to β), we use the “relative error” defined as

70 $$\mathrm{relative}\;\mathrm{error}=\frac{∥M^{\mathrm{approx}}-M^{\mathrm{exact}}∥}{∥M^{\mathrm{exact}}∥}$$

as a quality measure. In this equation, the double vertical bars denote the Frobenius norm.

For TM complexes parametrized by eq 53, we can choose the principal axis frame of the **D**-tensor such that the field-free Hamiltonian is described by only two parameters *D* and *E*/*D*. Apart from these, the spin dyadic depends on the spin quantum number *S* and on the temperature *T*. We derive in the Supporting Information Section S3 that the *relative error* (in %) of a truncated power expansion of the dyadic compared to the exact dyadic stays constant when scaling the constant *D* and the temperature *T* by the same factor. We also checked this numerically. This means that the relative error only depends on the ratio *D*/*k*_B_ *T* and not on *D* and *T* separately. Since we will present results as a function of *D*/*k*_B_ *T*, we do not need to distinguish between different values of *D*: the analysis is valid for all possible values. Below, we still separately investigate positive and negative values of *D* (i.e., positive and negative values of *D*/*k*_B_ *T*) for clarity. For the rhombicity *E*/*D* we consider three different values: 0 (axial), 1/6 (intermediate rhombicity), and 1/3 (maximal rhombicity). We consider the total spin quantum numbers *S* = 1, *S* = 3/2, *S* = 2, and *S* = 5/2. These are all nontrivial cases that can occur when the unpaired electrons occupy metal *d*-orbitals. We do not consider the case *S* = 1/2 because it is trivial: the first-order term (linear in β) already gives the exact dyadic and all higher-order terms are zero.

Regarding Ln complexes, we investigate the isostructural series of complexes for which Suturina et al. found a breakdown of Bleaney’s theory;^25^ see Figure 1. For our investigation, we use the ligand-field parameters obtained by them from *ab initio* complete active space self-consistent field (CASSCF) calculations.

**Figure 1:**
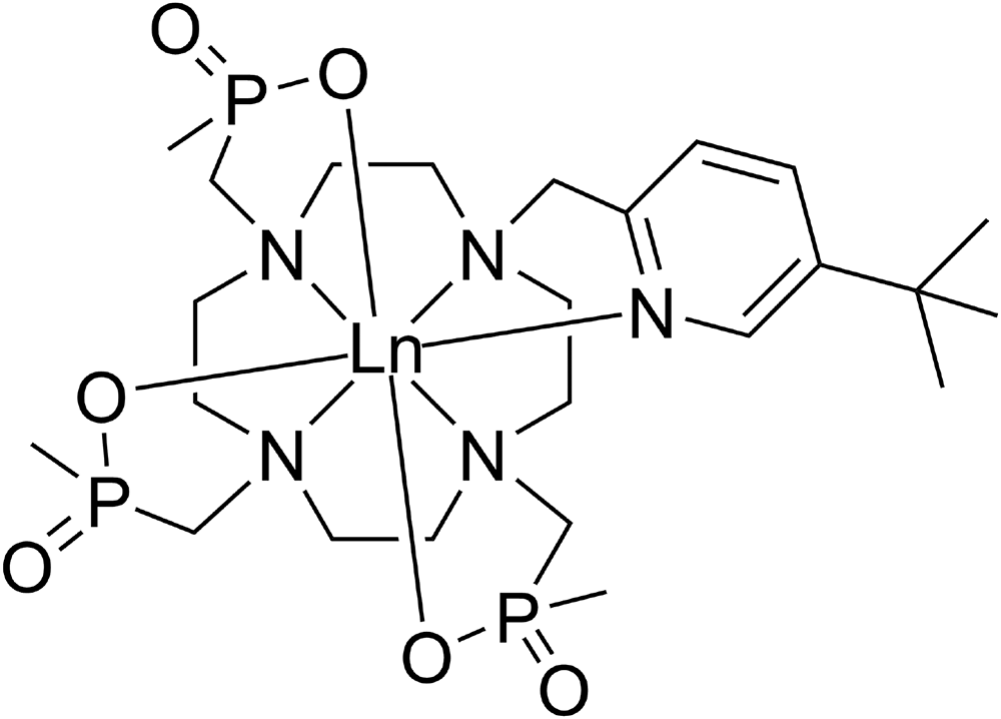
Isostructural series of Ln complexes of Suturina et al.^25^

### 3.2 Comparison of Analytical and Numerical Derivatives

In order to examine the convergence behavior of the dyadic, we implemented numerical derivatives up to sixth order using 12-point stencils with spacing *h* between the evaluation points. For completeness, we report two alternative derivations of the finite-difference equations we used in the Supporting Information Section S1.

For testing the correctness of the newly derived analytical equation for the third derivative of the angular momentum dyadic and for finding optimal finite difference values *h* for evaluating the numerical derivatives, we plotted the relative error in the first three numerical derivatives compared to the corresponding analytical derivatives as a function of *h*. This is shown for two specific examples (one TM and one Ln example) in Figure 2. The results for other cases can be found in the Supporting Information Section S4.

**Figure 2:**
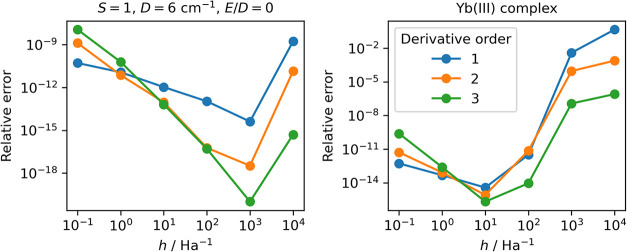
Relative error in the first three numerical derivatives of the spin dyadic as a function of the finite difference *h*. Left: TM case with *S* = 1, *D* = 6 cm^–1^ and *E*/*D* = 0. Right: ytterbium­(III) complex.

In these plots, one can observe the “V” shape that is typical for numerical derivatives, where there is an optimal *h* value that balances truncation errors (that grow large for too large *h* values) and numerical precision errors (that grow large for too small *h* values). Furthermore, in all cases one can choose an *h* value that makes the relative error in all three derivatives very small (below 10^–10^). These small deviations between numerical and analytical derivatives confirm the correctness of the newly derived analytical equations.

We found that the optimal *h* value (average of the optimal *h* values for the three derivatives) was typically in the range between 10 and 100 Ha^–1^ for the Ln complexes and between 100 and 1000 Ha^–1^ for the TM cases. Note that these numbers are considered “small” because ParaMag.jl internally works with atomic units. For example, at room temperature (*T* = 298 K), β = 1/*k*_B_ *T* ≈ 1060 Ha^–1^. Since we found the optimal *h* value to be relatively insensitive to the order of the derivative, we assume that the same *h* values can also be used for the numerical evaluation of higher-order derivatives.

### 3.3 Convergence of the Expansion of the Spin Dyadic in Powers of β

Next, we investigated the relative error of the dyadic approximated via truncated expansions in powers of β as a function of *T* (for the Ln complexes) or *D*/*k*_B_ *T* (for the TM cases). This was done for the complete test sets introduced above. The full set of convergence plots for all combinations of parameters and all Ln complexes from our test sets can be found in the Supporting Information Section S4.

For the TM cases, we could generally observe that the rate of convergence depends very little on the sign of *D* and the rhombicity *E*/*D*. Only the total spin quantum number *S* had a marked influence: the larger *S*, the slower/worse is the convergence of the spin dyadic with the order in β. As an illustration, plots for *S* = 1 and for *S* = 5/2 (with positive *D* and vanishing rhombicity) are shown in Figure 3. For *S* = 1, it can be observed that the border between convergence and divergence of the expansion lies around a value of *D*/*k*_B_ *T* = 3. For a value of *D*/*k*_B_ *T* = 0.1, which corresponds to *D* ≈ 20 cm^–1^ at room temperature, already the second-order expansion (“Bleaney’s theory”) has a relative error of less than 0.1%, which is negligible. In contrast, for *S* = 5/2, (slow) divergence is already observed for a value of *D*/*k*_B_ *T* = 1. For *D*/*k*_B_ *T* = 0.1, Bleaney’s theory gives a relative error of 1.2% and the third-order dyadic is required to obtain an error of 0.2%.

**Figure 3:**
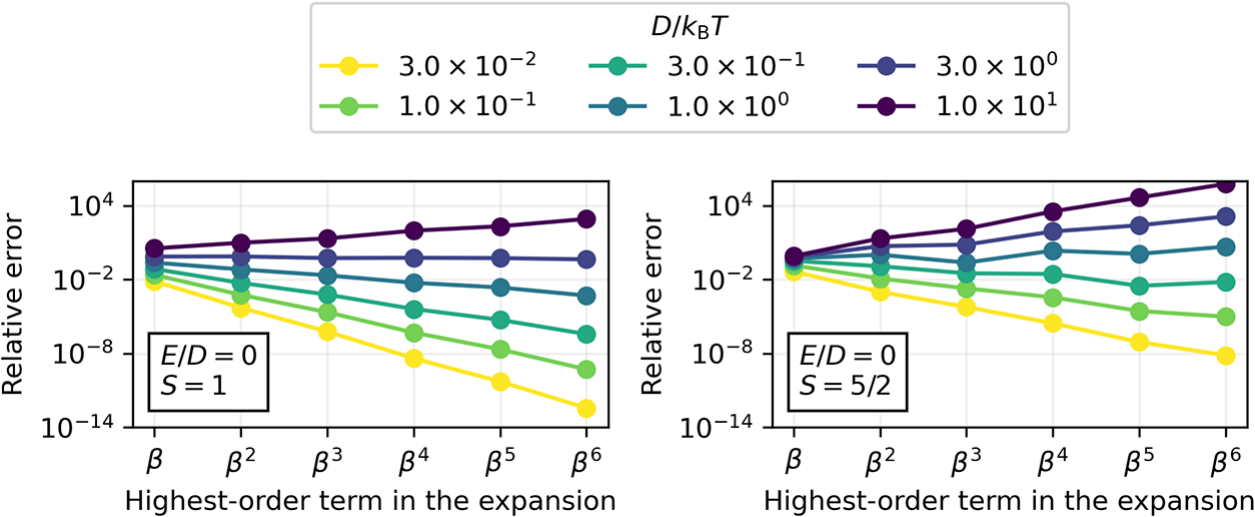
Relative error in the dyadic of a TM complex with positive *D*, *E*/*D* = 0 and *S* = 1 (left) or *S* = 2.5 (right) when it is truncated at different orders in β and evaluated for different *D*/*k*_B_ *T*.

For the Ln complexes, the rate of convergence of the dyadic with the order in β is correlated with the amount of ligand-field splitting as displayed by Suturina et al.^25^ The fastest convergence is observed for the holmium­(III) and erbium­(III) complexes, which have the smallest ligand-field splitting, whereas the slowest convergence (or fastest divergence) is observed for the dysprosium­(III) and ytterbium­(III) complexes, which have the largest ligand-field splitting. Overall, the holmium­(III) complex shows the fastest convergence and the ytterbium­(III) complex shows the slowest convergence (Figure 4). It is interesting that for the holmium­(III) complex, the expansion of the dyadic seems to converge even for the lowest tested temperature of 100 K. In contrast, for the ytterbium­(III) complex, convergence is only observed for temperatures of 200 K or higher. For lower temperatures, the expansion is outside its radius of convergence and the dyadic diverges. The traditional version of Bleaney’s theory for pseudocontact NMR shifts is mostly applied around room temperature, at which most solution NMR spectra are measured. At *T* = 300 K, the holmium­(III) complex has relative errors of 2.5% at second order (Bleaney’s theory) and 0.6% at third order. In contrast, the ytterbium­(III) complex has relative errors of 14.3% at second order and still 7.3% at third order. However, note that these errors also include the isotropic part of the dyadic, which is irrelevant for the calculation of pseudocontact shifts in the point-dipole approximation. In the next section, we examine the susceptibility anisotropy, which only depends on the anisotropic part of the angular momentum dyadic.

**Figure 4:**
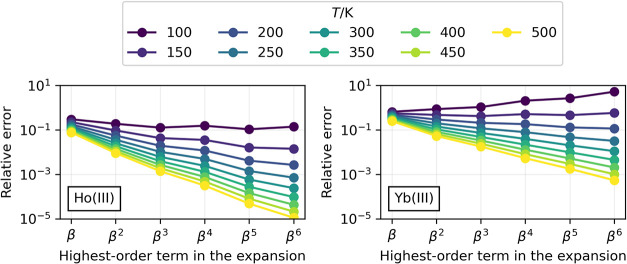
Relative error in the dyadic of the holmium­(III) complex (left) and ytterbium­(III) complex (right) when it is truncated at different orders in β and evaluated at different temperatures.

### 3.4 Susceptibility Anisotropy

Figure 4 shows the entire error made in approximating the angular momentum dyadic. For pseudocontact NMR shifts, the original property for which Bleaney derived his high-temperature expansion, only the *anisotropic* part of the dyadic/susceptibility tensor is relevant. The susceptibility anisotropies are defined in eqs 47 and 48.

Figure 5 shows χ_ax_ as well as the ratio χ_rh_/χ_ax_ (with values between 0 for axial systems and 1/3 for extremely rhombic systems) for the complexes in Suturina et al.’s isostructural series at room temperature (298 K). We compare the exact dyadic with the second-order (Bleaney’s theory) and third-order (our new analytical equation) approximation as well as the *ab initio* CASSCF level of theory.

**Figure 5:**
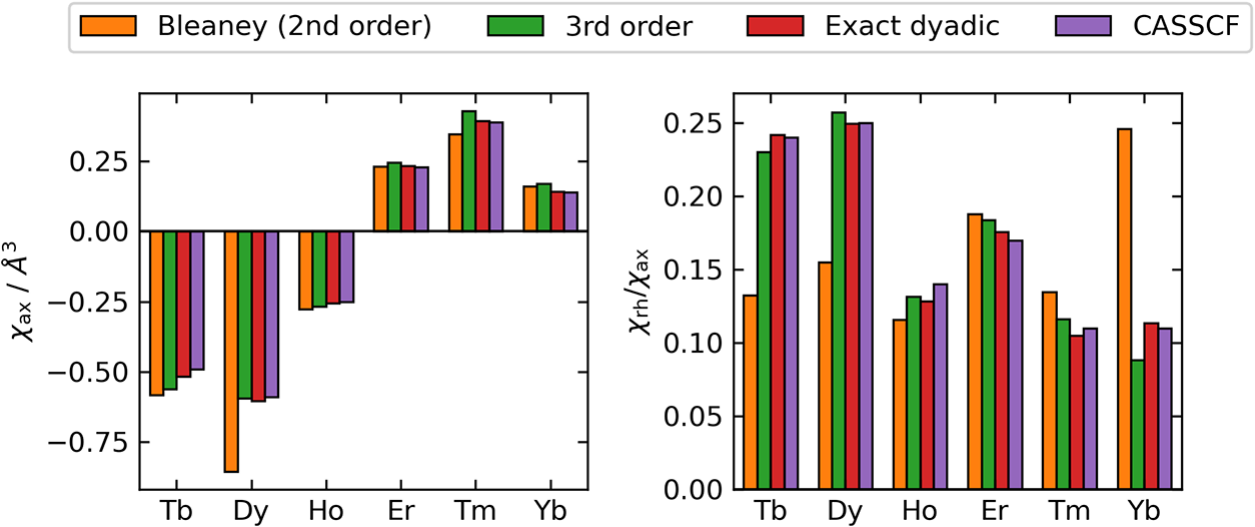
Axial susceptibility anisotropy (left) and ratio of rhombic and axial susceptibility anisotropies (right) for the Ln complexes calculated with four different approaches: the first three use the model Hamiltonian eq 2 and a second-order expansion (Bleaney’s theory), a third-order expansion, or the exact (“infinite order”) dyadic. We chose the ligand-field parameters obtained by Suturina et al.^25^ from CASSCF calculations. The fourth value for each complex is the CASSCF number reported by Suturina et al., which does not employ any effective Hamiltonian.

Generally, it can be observed that the exact dyadic agrees closely with the CASSCF values. The small deviation is due to inherent limitations of the model Hamiltonian.

For χ_ax_, Bleaney’s theory (second-order expansion) already gives a qualitatively good agreement with the exact dyadic for all complexes except for the dysprosium­(III) complex. For this system, Bleaney’s theory grossly overestimates the magnitude of χ_ax_. In contrast, the third-order dyadic derived in this work leads to an excellent agreement.

For the ratio χ_rh_/χ_ax_, Bleaney’s theory gives qualitatively wrong results for three of the complexes: terbium­(III), dysprosium­(III), and ytterbium­(III). In all these cases, the third-order dyadic derived herein significantly improves the agreement with the exact dyadic. The largest remaining discrepancy at third order is observed for ytterbium­(III), where χ_rh_/χ_ax_ is still underestimated by around 22%.

## 4 Discussion

It is sometimes stated in the literature that the field-free energy splitting needs to be much smaller than *k*_B_ *T* for Bleaney’s theory to be applicable.^24,27^ Our data suggest that the expansion of the dyadic in powers of β can converge even in cases where these two energies are of similar size or even when the field-free energy splitting is slightly larger than *k*_B_ *T*. However, in these situations the convergence is relatively slow and one might have to include higher-order terms in the expansion to obtain acceptable errors. In the *S* = 1 TM case (Figure 3), Bleaney’s theory leads to an error below 1% if *D*/*k*_B_ *T* ≤ 0.3, which is smaller than 1 but not much smaller. For all Ln complexes investigated herein, the ligand-field splitting is larger than *k*_B_ *T* at room temperature (see Figure 2 of Suturina et al.^25^). Still, for the fastest-converging Ho­(III) complex, the error of Bleaney’s theory at 300 K is only 2.5%, which leads to a qualitatively correct dyadic. Again, the statement that the ligand-field splitting needs to be *much smaller* than *k*_B_ *T* does not seem to hold.

The strong dependence of the speed of convergence on the spin quantum number *S* observed for the TM cases makes sense in light of the analytical equation up to third order (eq 67). From this equation, one sees that the coefficients scale as *S* ^2^ for the linear term, *S* ^4^ for the second-order term and *S* ^6^ for the third-order term. If this trend generalizes to higher orders, it means that for larger *S*, the relative importance of higher-order terms compared to the lower-order terms increases, which explains the slower convergence.

The third-order expansion, for which we derived a new analytical equation, gives qualitatively and in some cases even quantitatively correct susceptibility anisotropies for most of the complexes of the isostructural Ln series investigated here. However, this comes at the price of an increased complexity. While Bleaney’s theory (second-order expansion) leads to a one-to-one correspondence between the second-order ligand-field parameters and the components of the susceptibility tensor via a linear relationship, the third-order contribution to the susceptibility is quadratic in ligand-field parameters. From the analytical equation (eq 28), it can be seen that there are in total five different third-order contributions to the anisotropy of the angular momentum dyadic: three where *B*_*q*_^(*k*)^ parameters of the same order are coupled (for *k* = 2, 4, 6) and two where the parameters are coupled with those whose order *k* is 2 smaller (for *k* = 4, 6). This means that the next-order correction to Bleaney’s theory is considerably more complex than Bleaney’s theory itself.

In order to calculate the exact angular momentum dyadic, the field-free Hamiltonian has to be diagonalized, which leads to a very intransparent dependence on the ligand-field parameters. In contrast, our new analytical third-order equations have the advantage that the dependence is much more explicit. In particular in high-symmetry situations, where many of the ligand-field parameters are exactly zero, an analysis of the dyadic in terms of this new analytical equation might be advantageous.

## 5 Conclusions

We generalized Bleaney’s theory—originally derived as a high-temperature approximation for pseudocontact NMR shifts valid up to order 1/*T* ^2^—in two ways. First, we formulated our derivation in terms of the angular momentum (or spin) dyadic for arbitrary effective Hamiltonians parametrized in terms of angular momentum (or spin) operators. This encompasses not only the lowest *J*-manifold of Ln complexes but also the lowest spin multiplet of TM complexes and extends the validity to a larger range of properties, for example, chemical shielding tensors beyond the point-dipole approximation. Second, we derived analytical expressions for the 1/*T* ^3^ contribution in terms of Hamiltonian parameters, which extends the validity of the theory to lower temperatures and/or larger field-free energy splittings. Furthermore, in order to investigate the convergence behavior of the expansion in powers of 1/*T*, we obtained even higher-order terms using numerical derivatives of the exact dyadic.

The analytical third-order equation obtained by us generalizes the analytical equations derived by McGarvey^19^ to situations where the principal axis frame is not known a priori. In addition, our equation is much more compact than McGarvey’s expressions, which facilitates its application. Applying this equation to the TM case, we could unify the equations for contact and pseudocontact isotropic shieldings that were separately derived for each spin quantum number *S* by Martin and Autschbach.^28^

Our investigation of the convergence behavior of the dyadic revealed, as expected, that the expansion in powers of 1/*T* converges more slowly and eventually diverges if the ratio of the field-free energy splittings and the temperature is too large. However, we still found convergent behavior in situations where the two are of a similar order of magnitude, as exemplified by the Ln series of Suturina et al.^25^ This shows that the field-free energy splittings do not need to be “much smaller” than *k*_B_ *T*, contrary to what is often claimed in the literature. However, one should admit that if the convergence is slow, low-order truncations will be inaccurate and it is more practical to employ the exact dyadic instead.

Our results indicate that for the amount of zero-field splitting typically found in TM complexes, the second- or third-order dyadic will be sufficient for accurate results. For the Ln series, where a failure of Bleaney’s theory was reported in the past,^25^ we found that our analytical third-order equation significantly improves the calculated susceptibility anisotropies, giving semiquantitative results. These analytical equations up to third order have the advantage that the influence of the Hamiltonian parameters on the magnetic properties is much more transparent than when using the exact dyadic, the latter of which requires the diagonalization of the Hamiltonian.

The expansion up to the third order should still be used with caution for some Ln complexes with large ligand-field splittings. In practice, one should always check the validity of such a truncated expansion by comparing with the results obtained with the exact dyadic. If the results agree, the analytical equations make it more transparent how different Hamiltonian parameters influence the calculated properties.

Finally, we should mention once more that we only investigated the validity of truncated expansions of temperature-dependent magnetic properties in powers of 1/*T*. Other questionable assumptions often associated with Bleaney’s theory, for example, the transferability of ligand-field parameters across an isostructural series, were not investigated here.

## Supplementary Material





## Figures and Tables

**Figure 6:**
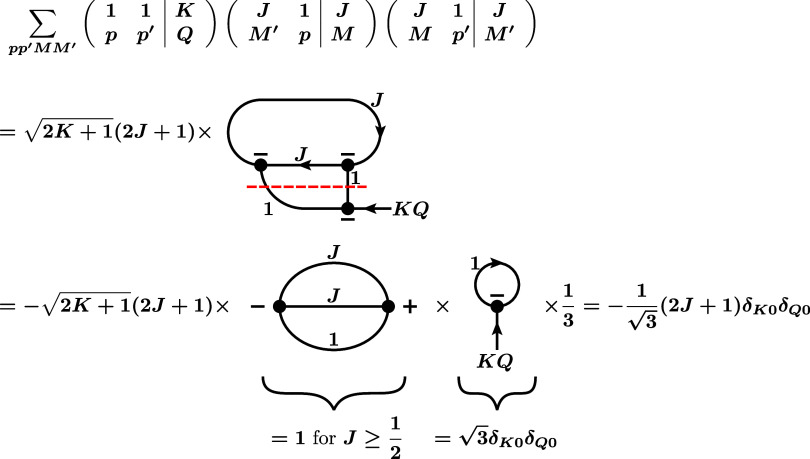
Graphical evaluation of the contraction of CGCs occurring in the first derivative of the angular momentum dyadic. The red dashed line indicates how the diagram is factorized.

**Figure 7:**
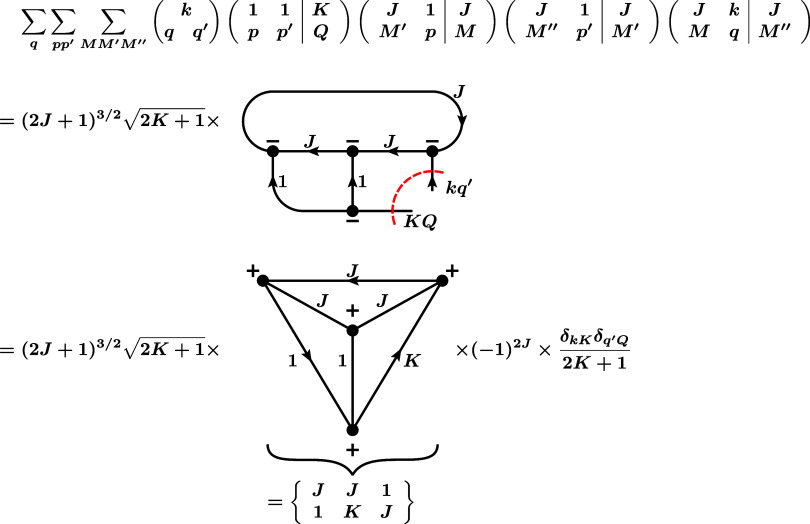
Graphical evaluation of the contraction of antisymmetric symbols and CGCs occurring in the second derivative of the angular momentum dyadic. The red dashed line indicates how the diagram is factorized.

**Figure 8:**
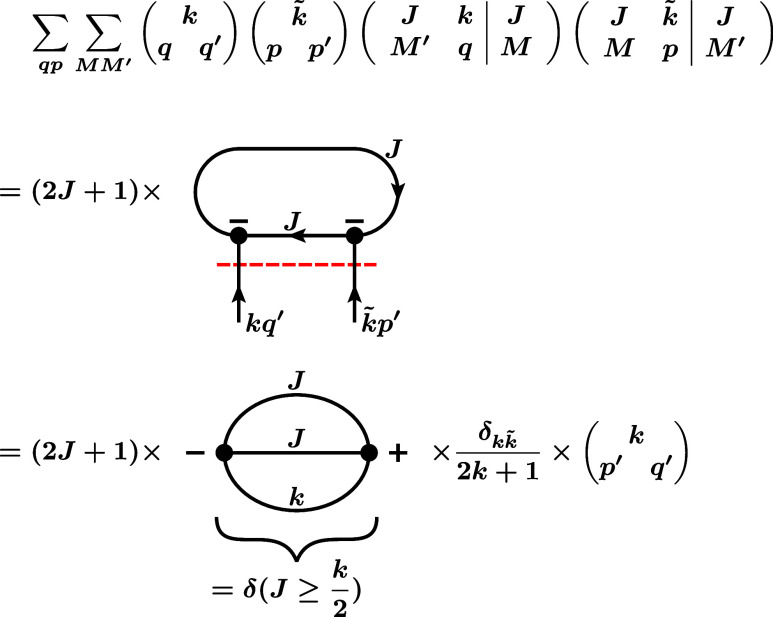
Graphical evaluation of the contraction of antisymmetric symbols and CGCs occurring in the first part of the third derivative of the angular momentum dyadic. The red dashed line indicates how the diagram is factorized.

**Figure 9:**
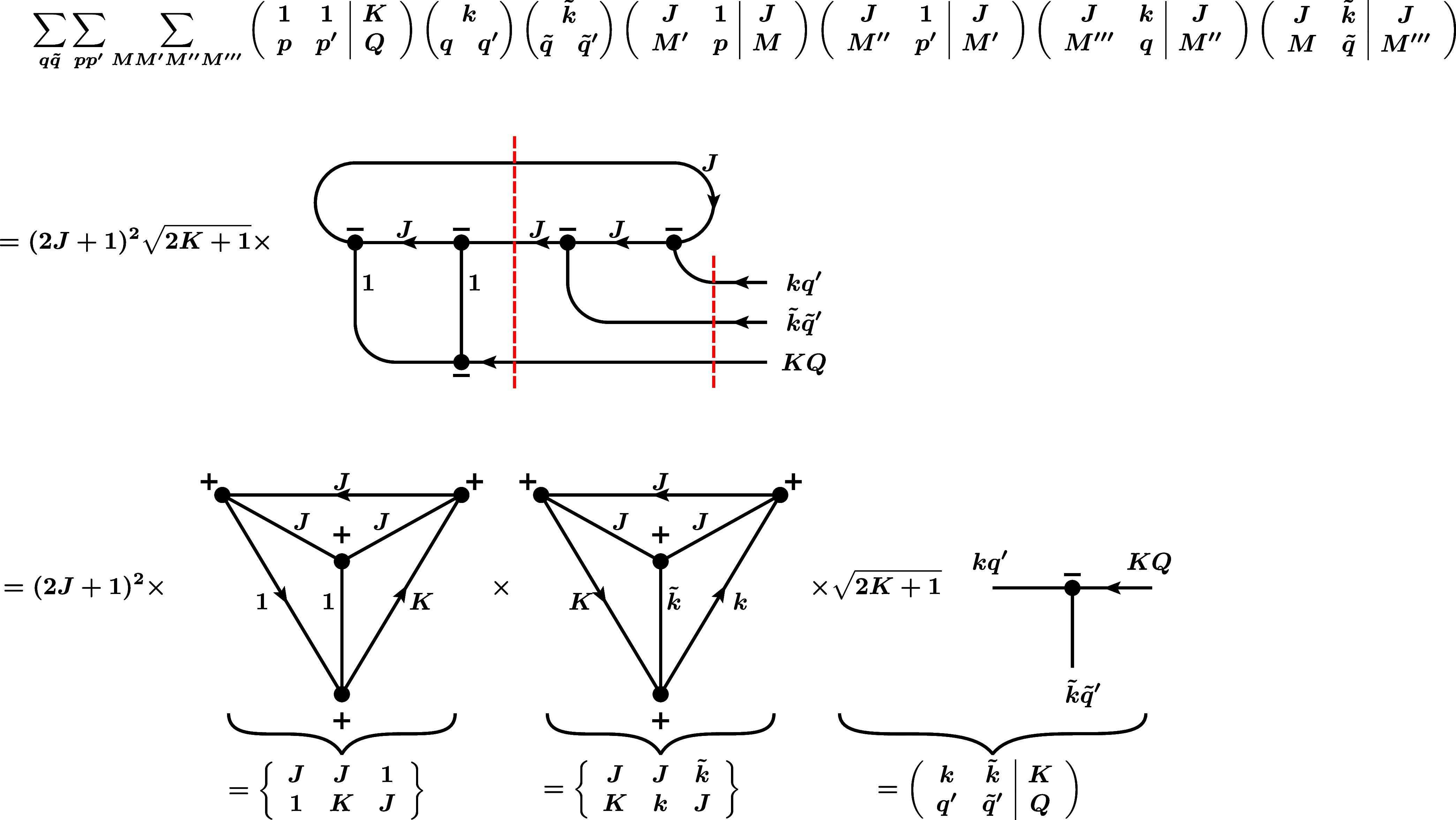
Graphical evaluation of the contraction of antisymmetric symbols and CGCs occurring in the second part of the third derivative of the angular momentum dyadic. The red dashed lines indicate how the diagram is factorized.

**Figure 10:**
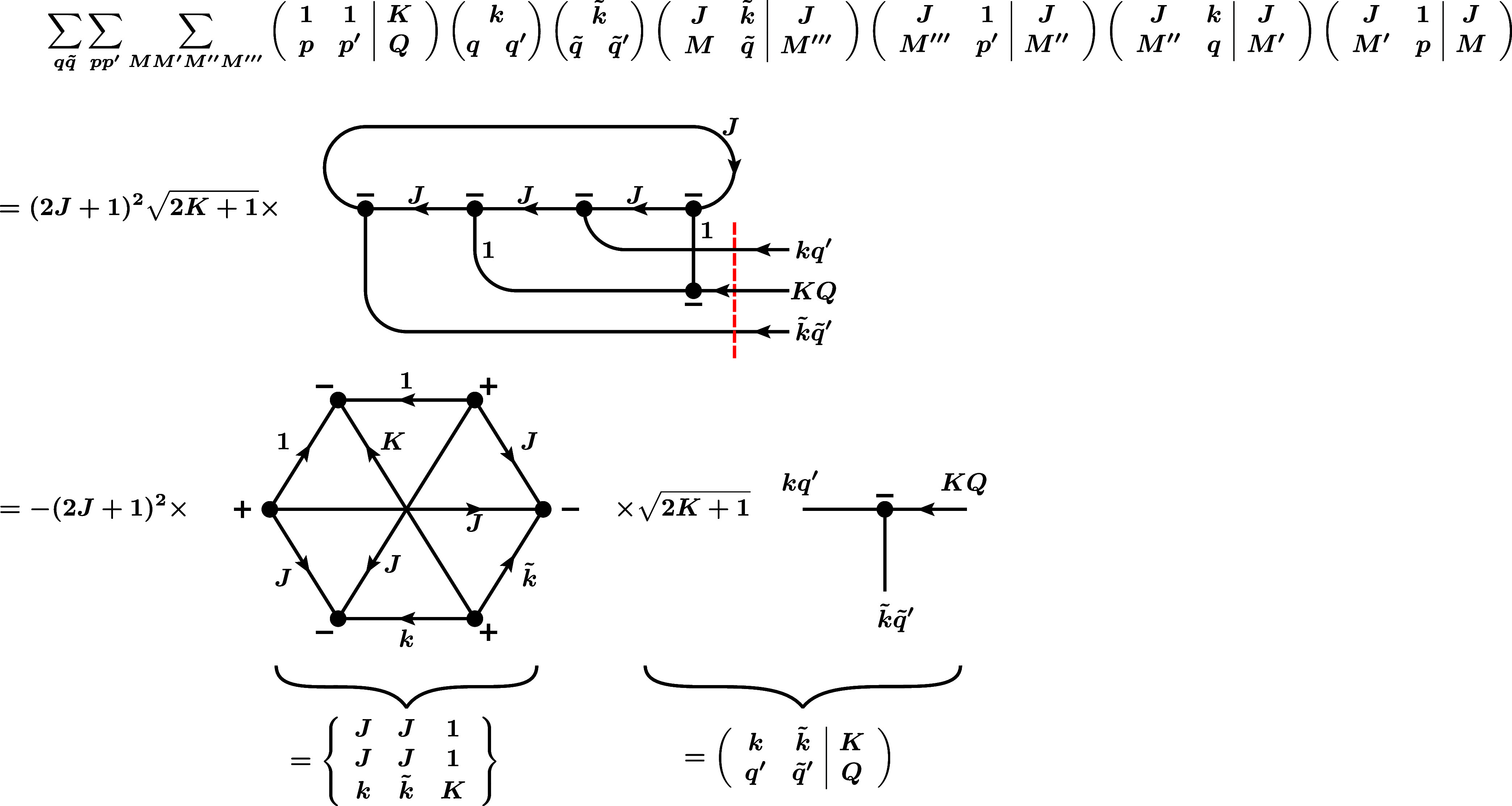
Graphical evaluation of the contraction of antisymmetric symbols and CGCs occurring in the third part of the third derivative of the angular momentum dyadic. The red dashed line indicates how the diagram is factorized.

## Data Availability

The data underlying this study are openly available on Zenodo at 10.5281/zenodo.18659734. All calculation and analysis steps were performed with a Snakemake^50^ workflow that can be obtained from https://github.com/LucasLang/Bleaney_analysis and 10.5281/zenodo.18659655.

## A: Model-Agnostic Equations for the First Three Derivatives of ⟨⟨*H*_*k*_ *H*_*l*_⟩⟩

The first three derivatives of eq 9 with respect to β are given by

71
$$\frac{\partial \left\langle \left\langle H_{k}H_{l}\right\rangle \right\rangle }{\partial \beta }=-\left(\frac{\partial }{\partial \beta }\frac{1}{Z}\right)\int_{0}^{\beta }\mathrm{tr}\left(H_{k}e^{-\left(\beta -\omega \right)H}H_{l}e^{-\omega H}\right)d\omega -\frac{1}{Z}\int_{0}^{\beta }\mathrm{tr}\left(H_{k}\left(-H\right)e^{-\left(\beta -\omega \right)H}H_{l}e^{-\omega H}\right)d\omega -\frac{1}{Z}\mathrm{tr}\left(H_{k}H_{l}e^{-\beta H}\right)$$

72
$$\frac{\partial^{2}\left\langle \left\langle H_{k}H_{l}\right\rangle \right\rangle }{\partial \beta^{2}}=-\left(\frac{\partial^{2}}{\partial \beta^{2}}\frac{1}{Z}\right)\int_{0}^{\beta }\mathrm{tr}\left(H_{k}e^{-\left(\beta -\omega \right)H}H_{l}e^{-\omega H}\right)d\omega -2\left(\frac{\partial }{\partial \beta }\frac{1}{Z}\right)\int_{0}^{\beta }\mathrm{tr}\left(H_{k}\left(-H\right)e^{-\left(\beta -\omega \right)H}H_{l}e^{-\omega H}\right)d\omega -2\left(\frac{\partial }{\partial \beta }\frac{1}{Z}\right)\mathrm{tr}\left(H_{k}H_{l}e^{-\beta H}\right)-\frac{1}{Z}\int_{0}^{\beta }\mathrm{tr}\left(H_{k}{\left(-H\right)}^{2}e^{-\left(\beta -\omega \right)H}H_{l}e^{-\omega H}\right)d\omega -\frac{1}{Z}\mathrm{tr}\left(H_{k}\left(-H\right)H_{l}e^{-\beta H}\right)-\frac{1}{Z}\mathrm{tr}\left(H_{k}H_{l}\left(-H\right)e^{-\beta H}\right)$$

73
$$\frac{\partial^{3}\left\langle \left\langle H_{k}H_{l}\right\rangle \right\rangle }{\partial \beta^{3}}=\int_{0}^{\beta }\cdot \cdot \cdot d\omega -3\left(\frac{\partial }{\partial \beta }\frac{1}{Z}\right)\mathrm{tr}\left(H_{k}\left(-H\right)H_{l}e^{-\beta H}\right)-3\left(\frac{\partial^{2}}{\partial \beta^{2}}\frac{1}{Z}\right)\mathrm{tr}\left(H_{k}H_{l}e^{-\beta H}\right)-3\left(\frac{\partial }{\partial \beta }\frac{1}{Z}\right)\mathrm{tr}\left(H_{k}H_{l}\left(-H\right)e^{-\beta H}\right)-\frac{1}{Z}\mathrm{tr}\left(H_{k}{\left(-H\right)}^{2}H_{l}e^{-\beta H}\right)-\frac{1}{Z}\mathrm{tr}\left(H_{k}\left(-H\right)H_{l}\left(-H\right)e^{-\beta H}\right)-\frac{1}{Z}\mathrm{tr}\left(H_{k}H_{l}{\left(-H\right)}^{2}e^{-\beta H}\right)$$

In the third derivative, we did not write out the terms with a remaining integral explicitly, because they vanish upon setting β = 0. We still need expressions for the derivatives of 1/*Z*. These are given by

74
$$\frac{\partial }{\partial \beta }\frac{1}{Z}=-\frac{\frac{\partial Z}{\partial \beta }}{Z^{2}}$$

75
$$\frac{\partial^{2}}{\partial \beta^{2}}\frac{1}{Z}=-\frac{\frac{\partial^{2}Z}{\partial \beta^{2}}}{Z^{2}}+2\frac{{\left(\frac{\partial Z}{\partial \beta }\right)}^{2}}{Z^{3}}$$

with

76
$$Z=\mathrm{tr}\left(e^{-\beta H}\right)$$

77
$$\frac{\partial Z}{\partial \beta }=\mathrm{tr}\left(\left(-H\right)e^{-\beta H}\right)$$

78
$$\frac{\partial^{2}Z}{\partial \beta^{2}}=\mathrm{tr}\left({\left(-H\right)}^{2}e^{-\beta H}\right)$$

Evaluating the derivatives at β = 0 and assuming that the Hamiltonian is traceless, tr­(*H*) = 0, one obtains

79
$${\left(\frac{1}{Z}\right)}_{\beta =0}=\frac{1}{\left|H\right|}$$

80
$${\left(\frac{\partial }{\partial \beta }\frac{1}{Z}\right)}_{\beta =0}=0$$

81
$${\left(\frac{\partial^{2}}{\partial \beta^{2}}\frac{1}{Z}\right)}_{\beta =0}=-\frac{\mathrm{tr}\left(H^{2}\right)}{|H{|}^{2}}$$

Here, we used the notation |H| for the dimension of the Hilbert space. Combining these results directly leads to

82
$$\langle {\left\langle H_{k}H_{l}\right\rangle \rangle }_{\beta =0}=0$$

83
$${\left(\frac{\partial \left\langle \left\langle H_{k}H_{l}\right\rangle \right\rangle }{\partial \beta }\right)}_{\beta =0}=-\frac{1}{\left|H\right|}\mathrm{tr}\left(H_{k}H_{l}\right)$$

84
$${\left(\frac{\partial^{2}\left\langle \left\langle H_{k}H_{l}\right\rangle \right\rangle }{\partial \beta^{2}}\right)}_{\beta =0}=\frac{1}{\left|H\right|}\mathrm{tr}\left[\left(H_{k}H_{l}+H_{l}H_{k}\right)H\right]$$

85
$${\left(\frac{\partial^{3}\left\langle \left\langle H_{k}H_{l}\right\rangle \right\rangle }{\partial \beta^{3}}\right)}_{\beta =0}=3\frac{\mathrm{tr}\left(H^{2}\right)}{|H{|}^{2}}\mathrm{tr}\left(H_{k}H_{l}\right)-\frac{1}{\left|H\right|}\mathrm{tr}\left[\left(H_{k}H_{l}+H_{l}H_{k}\right)H^{2}\right]-\frac{1}{\left|H\right|}\mathrm{tr}\left(H_{k}HH_{l}H\right)$$

These equations are valid for any thermal linear response function and in particular for the susceptibility tensor (Van Vleck equation)^48^ and the chemical shielding tensor (Van den Heuvel–Soncini equation^33^ without diamagnetic term).

## B: First Three Derivatives of the Angular Momentum Dyadic in Terms of Spherical Tensor Parameters

### B.1 First Derivative

Using eq 15, the first derivative of a spherical component of the angular momentum dyadic (eq 24) is given by

86
(∂⟨⟨JJT⟩⟩Q(K)∂β)β=0=∑pp′(11pp′|KQ)(∂⟨⟨Jp(1)Jp′(1)⟩⟩∂β)β=0=−12J+1∑pp′(11pp′|KQ)tr(Jp(1)Jp′(1))=−12J+1∑pp′(11pp′|KQ)∑MM′⟨JM|Jp(1)|JM′⟩⟨JM′|Jp′(1)|JM⟩=−12J+1⟨J||J(1)||J⟩2∑pp′MM′(11pp′|KQ)(J1M′p|JM)(J1Mp′|JM′)

Here, we first used the *J*_*z*_ eigenbasis |*JM*⟩ to evaluate the trace and then replaced the matrix elements of the spherical angular momentum operators using the Wigner–Eckart theorem. The remaining contraction over CGCs can in principle be evaluated algebraically, but a particularly intuitive alternative that we will employ is the use of graphical methods.^35,36^ Using these angular momentum diagrams, the contraction of the CGCs can be reformulated as shown in Figure 6.

The value of the reduced matrix element is

87
$$\left\langle J∥J^{\left(1\right)}∥J\right\rangle =\sqrt{J\left(J+1\right)}$$

which leads to the final result given in eq 25 of the main text.

### B.2 Second Derivative

Using eq 16, the second derivative is given by

88
$${\left(\frac{\partial^{2}\langle {\left\langle {\mathrm{JJ}}^{T}\rangle \right\rangle }_{Q}^{\left(K\right)}}{\partial \beta^{2}}\right)}_{\beta =0}=\sum_{\mathrm{pp}\prime }\left(\begin{array}{ll} 1 & 1\\ p & p^{\prime } \end{array}|\begin{array}{l} K\\ Q \end{array}\right){\left(\frac{\partial^{2}\left\langle \left\langle J_{p}^{\left(1\right)}J_{p\prime }^{\left(1\right)}\right\rangle \right\rangle }{\partial \beta^{2}}\right)}_{\beta =0}=\frac{1}{2J+1}\sum_{\mathrm{pp}\prime }\left(\begin{array}{ll} 1 & 1\\ p & p^{\prime } \end{array}|\begin{array}{l} K\\ Q \end{array}\right)\mathrm{tr}\left[\left(J_{p}^{\left(1\right)}J_{p\prime }^{\left(1\right)}+J_{p\prime }^{\left(1\right)}J_{p}^{\left(1\right)}\right)H^{\left(0\right)}\right]$$

Since *K* = 0 or *K* = 2, the CGC is symmetric in the first two columns, i.e.

89
$$\left(\begin{array}{ll} 1 & 1\\ p & p^{\prime } \end{array}|\begin{array}{l} K\\ Q \end{array}\right)=\left(\begin{array}{ll} 1 & 1\\ p^{\prime } & p \end{array}|\begin{array}{l} K\\ Q \end{array}\right)$$

This means that the two terms inside the trace can be combined

90
$${\left(\frac{\partial^{2}\langle {\left\langle {\mathrm{JJ}}^{T}\rangle \right\rangle }_{Q}^{\left(K\right)}}{\partial \beta^{2}}\right)}_{\beta =0}=\frac{2}{2J+1}\sum_{\mathrm{pp}\prime }\left(\begin{array}{ll} 1 & 1\\ p & p^{\prime } \end{array}|\begin{array}{l} K\\ Q \end{array}\right)\mathrm{tr}\left(J_{p}^{\left(1\right)}J_{p\prime }^{\left(1\right)}H^{\left(0\right)}\right)$$

Inserting the field-free Hamiltonian written in terms of the antisymmetric symbol, evaluating the trace in the *J*_*z*_ eigenbasis, and employing the Wigner–Eckart theorem, one obtains

$${\left(\frac{\partial^{2}\langle {\left\langle {\mathrm{JJ}}^{T}\rangle \right\rangle }_{Q}^{\left(K\right)}}{\partial \beta^{2}}\right)}_{\beta =0}=\frac{2J\left(J+1\right)\left\langle J||O^{\left(k\right)}||J\right\rangle }{2J+1}\underset{\mathrm{kq}\prime }{\sum }B_{q\prime }^{\left(k\right)}\times \underset{q}{\sum }\underset{\mathrm{pp}\prime }{\sum }\underset{\mathrm{MM}\prime M\prime \prime }{\sum }\left(\begin{array}{c} k\\ q\;q^{\prime } \end{array})(\begin{array}{cc} 1 & 1\\ p & p^{\prime } \end{array}|\begin{array}{c} K\\ Q \end{array}\right)\left(\begin{array}{ll} J & 1\\ M^{\prime } & p \end{array}|\begin{array}{l} J\\ M \end{array}\right)\left(\begin{array}{ll} J & 1\\ M^{\prime \prime } & p^{\prime } \end{array}|\begin{array}{l} J\\ M^{\prime } \end{array}\right)\left(\begin{array}{cc} J & k\\ M & q \end{array}|\begin{array}{l} J\\ M^{\prime \prime } \end{array}\right)$$
91

The contraction of the CGCs and the antisymmetric symbol can again be evaluated using angular momentum graphs, as demonstrated in Figure 7.

This leads to

$${\left(\frac{\partial^{2}\langle {\left\langle {\mathrm{JJ}}^{T}\rangle \right\rangle }_{Q}^{\left(K\right)}}{\partial \beta^{2}}\right)}_{\beta =0}=2J\left(J+1\right)\left\langle J||O^{\left(K\right)}||J\right\rangle {\left(-1\right)}^{2J}\left\{\begin{array}{lll} J & J & 1\\ 1 & K & J \end{array}\right\}\sqrt{\frac{2J+1}{2K+1}}B_{Q}^{\left(K\right)}$$
92

Since we chose the Hamiltonian traceless, *B*_0_^(0)^ = 0 and the second derivative of the *K* = 0 part of the dyadic is zero. For the remaining *K* = 2 part, the 6*j* symbol can be written as

93
$${\left(-1\right)}^{2J}\left\{\begin{array}{lll} J & J & 1\\ 1 & 2 & J \end{array}\right\}=\delta \left(J\geq 1\right)\sqrt{\frac{\left(2J-1\right)\left(2J+3\right)}{30J\left(J+1\right)\left(2J+1\right)}}$$

and the BST operator RME can be written as

94
$$\left\langle J∥O^{\left(2\right)}∥J\right\rangle =\frac{1}{2}\sqrt{J\left(J+1\right)\left(2J-1\right)\left(2J+3\right)}$$

which is a special case of eq 5. Inserting both gives eq 26 of the main text.

### B.3 Third Derivative

The third derivative

95
$${\left(\frac{\partial^{3}\langle {\left\langle {\mathrm{JJ}}^{T}\rangle \right\rangle }_{Q}^{\left(K\right)}}{\partial \beta^{3}}\right)}_{\beta =0}=\sum_{\mathrm{pp}\prime }\left(\begin{array}{ll} 1 & 1\\ p & p^{\prime } \end{array}|\begin{array}{l} K\\ Q \end{array}\right){\left(\frac{\partial^{3}\left\langle \left\langle J_{p}^{\left(1\right)}J_{p\prime }^{\left(1\right)}\right\rangle \right\rangle }{\partial \beta^{3}}\right)}_{\beta =0}$$

consists of three different parts, see eq 17, which we will treat separately.

#### First Part

The first part of the third derivative is basically identical to the first derivative (trace of the product of two angular momentum operators). What remains is the trace of the square of the field-free Hamiltonian, which we can write as

$$\mathrm{tr}\left(H^{\left(0\right)}H^{\left(0\right)}\right)=\underset{k\mathrm{k̃}}{\sum }\left\langle J∥O^{\left(k\right)}∥J\right\rangle \left\langle J∥O^{\left(\mathrm{k̃}\right)}∥J\right\rangle \underset{q\prime p\prime }{\sum }B_{q\prime }^{\left(k\right)}B_{p\prime }^{\left(\mathrm{k̃}\right)}\times \underset{\mathrm{qp}}{\sum }\underset{\mathrm{MM}\prime }{\sum }\left(\begin{array}{c} k\\ q\;q^{\prime } \end{array}\right)\left(\begin{array}{c} \mathrm{k̃}\\ p\;p^{\prime } \end{array}\right)\left(\begin{array}{ll} J & k\\ M^{\prime } & q \end{array}|\begin{array}{l} J\\ M \end{array}\right)\left(\begin{array}{ll} J & \mathrm{k̃}\\ M & p \end{array}|\begin{array}{l} J\\ M^{\prime } \end{array}\right)$$
96

The contraction of the CGCs and the antisymmetric symbols can be evaluated graphically as shown in Figure 8. This leads to

97
$$\mathrm{tr}\left(H^{\left(0\right)}H^{\left(0\right)}\right)=\sum_{k\leq 2J}\langle J||O^{\left(k\right)}||J\rangle^{2}\frac{2J+1}{2k+1}\sum_{q\prime p\prime }\left(\begin{array}{c} k\\ p^{\prime }\;q^{\prime } \end{array}\right)B_{p\prime }^{\left(k\right)}B_{q\prime }^{\left(k\right)}$$

The antisymmetric symbol can be expressed in terms of a CGC

$$\left(\begin{array}{c} k\\ p^{\prime }\;q^{\prime } \end{array}\right)=\sqrt{2k+1}\left(\begin{array}{ll} k & k\\ p^{\prime } & q^{\prime } \end{array}|\begin{array}{l} 0\\ 0 \end{array}\right)$$
98

Furthermore, we define

$${\left(B^{\left(k\right)}⊗B^{\left(\mathrm{k̃}\right)}\right)}_{Q}^{\left(K\right)}=\underset{q\mathrm{q̃}}{\sum }\left(\begin{array}{ll} k & \mathrm{k̃}\\ q & \mathrm{q̃} \end{array}|\begin{array}{l} K\\ Q \end{array}\right)B_{q}^{\left(k\right)}B_{\mathrm{q̃}}^{\left(\mathrm{k̃}\right)}$$
99

This quantity is a new spherical tensor of rank *K* constructed from products of Hamiltonian parameters. Using the last two equations, we can write

$$\mathrm{tr}\left(H^{\left(0\right)}H^{\left(0\right)}\right)=\underset{k\leq 2J}{\sum }\langle J||O^{\left(k\right)}||J\rangle^{2}\frac{2J+1}{\sqrt{2k+1}}{\left(B^{\left(k\right)}⊗B^{\left(k\right)}\right)}_{0}^{\left(0\right)}$$
100

The complete first part of the third derivative is given by

$${\left(\frac{\partial^{3}\langle {\left\langle {\mathrm{JJ}}^{T}\rangle \right\rangle }_{Q}^{\left(K\right)}}{\partial \beta^{3}}\right)}_{\beta =0}\leftarrow -\sqrt{3}J\left(J+1\right)\delta_{K0}\delta_{Q0}\underset{k\leq 2J}{\sum }\frac{\langle J∥O^{\left(k\right)}∥J\rangle^{2}}{\sqrt{2k+1}}{\left(B^{\left(k\right)}⊗B^{\left(k\right)}\right)}_{0}^{\left(0\right)}$$
101

#### Second Part

For the second part of the third derivative, we can again use eq 89 to combine two terms and write

102
$${\left(\frac{\partial^{3}\langle {\left\langle {\mathrm{JJ}}^{T}\rangle \right\rangle }_{Q}^{\left(K\right)}}{\partial \beta^{3}}\right)}_{\beta =0}\leftarrow -\frac{2}{2J+1}\sum_{\mathrm{pp}\prime }\left(\begin{array}{ll} 1 & 1\\ p & p^{\prime } \end{array}|\begin{array}{l} K\\ Q \end{array}\right)\mathrm{tr}\left(J_{p}^{\left(1\right)}J_{p\prime }^{\left(1\right)}H^{\left(0\right)}H^{\left(0\right)}\right)$$

Again, we can insert an eigenbasis, write the field-free Hamiltonian in terms of the antisymmetric symbol, and use the Wigner–Eckart theorem to arrive at

103
$${\left(\frac{\partial^{3}\langle {\left\langle {\mathrm{JJ}}^{T}\rangle \right\rangle }_{Q}^{\left(K\right)}}{\partial \beta^{3}}\right)}_{\beta =0}\leftarrow -\frac{2J\left(J+1\right)}{2J+1}\sum_{k\mathrm{k̃}}\left\langle J∥O^{\left(k\right)}J\right\rangle \left\langle J∥O^{\left(\mathrm{k̃}\right)}∥J\right\rangle \sum_{q\prime \mathrm{q̃}\prime }B_{q\prime }^{\left(k\right)}B_{\mathrm{q̃}\prime }^{\left(\mathrm{k̃}\right)}\times \sum_{q\mathrm{q̃}}\sum_{\mathrm{pp}\prime }\sum_{\mathrm{MM}\prime M\prime \prime M\prime \prime \prime }\left(\begin{array}{ll} 1 & 1\\ p & p^{\prime } \end{array}|\begin{array}{l} K\\ Q \end{array}\right)\left(\begin{array}{c} k\\ q\;q^{\prime } \end{array}\right)\left(\begin{array}{c} \mathrm{k̃}\\ \mathrm{q̃}\;{\mathrm{q̃}}^{\prime } \end{array}\right)\left(\begin{array}{ll} J & 1\\ M^{\prime } & p \end{array}|\begin{array}{l} J\\ M \end{array}\right)\times \left(\begin{array}{ll} J & 1\\ M^{\prime \prime } & p^{\prime } \end{array}|\begin{array}{l} J\\ M^{\prime } \end{array}\right)\left(\begin{array}{ll} J & k\\ M^{\prime \prime \prime } & q \end{array}|\begin{array}{l} J\\ M^{\prime \prime } \end{array}\right)\left(\begin{array}{ll} J & \mathrm{k̃}\\ M & \mathrm{q̃} \end{array}|\begin{array}{l} J\\ M^{\prime \prime \prime } \end{array}\right)$$

The contraction of the CGCs and the antisymmetric symbols can be evaluated as shown in Figure 9. The result is

$${\left(\frac{\partial^{3}\langle {\left\langle {\mathrm{JJ}}^{T}\rangle \right\rangle }_{Q}^{\left(K\right)}}{\partial \beta^{3}}\right)}_{\beta =0}\leftarrow -2J\left(J+1\right)\left(2J+1\right)\underset{k\mathrm{k̃}}{\sum }\left\langle J∥O^{\left(k\right)}∥J\right\rangle \left\langle J∥O^{\left(\mathrm{k̃}\right)}∥J\right\rangle \times \left\{\begin{array}{lll} J & J & 1\\ 1 & K & J \end{array}\right\}\left\{\begin{array}{lll} J & J & \mathrm{k̃}\\ K & k & J \end{array}\right\}{\left(B^{\left(k\right)}⊗B^{\left(\mathrm{k̃}\right)}\right)}_{Q}^{\left(K\right)}$$
104

#### Third Part

The third part of the third derivative can be written as

$${\left(\frac{\partial^{3}\langle {\left\langle {\mathrm{JJ}}^{T}\rangle \right\rangle }_{Q}^{\left(K\right)}}{\partial \beta^{3}}\right)}_{\beta =0}\leftarrow -\frac{J\left(J+1\right)}{2J+1}\underset{k\mathrm{k̃}}{\sum }\left\langle J∥O^{\left(k\right)}∥J\right\rangle \left\langle J∥O^{\left(\mathrm{k̃}\right)}∥J\right\rangle \underset{q\prime \mathrm{q̃}\prime }{\sum }B_{q\prime }^{\left(k\right)}B_{\mathrm{q̃}\prime }^{\left(\mathrm{k̃}\right)}\times \underset{q\mathrm{q̃}}{\sum }\underset{\mathrm{pp}\prime }{\sum }\underset{\mathrm{MM}\prime M\prime \prime M\prime \prime \prime }{\sum }\left(\begin{array}{ll} 1 & 1\\ p & p^{\prime } \end{array}|\begin{array}{l} K\\ Q \end{array}\right)\left(\begin{array}{c} k\\ q\;q^{\prime } \end{array}\right)\left(\begin{array}{c} \mathrm{k̃}\\ \mathrm{q̃}\;{\mathrm{q̃}}^{\prime } \end{array}\right)\left(\begin{array}{ll} J & \mathrm{k̃}\\ M & \mathrm{q̃} \end{array}|\begin{array}{l} J\\ M^{\prime \prime \prime } \end{array}\right)\times \left(\begin{array}{ll} J & 1\\ M^{\prime \prime \prime } & p^{\prime } \end{array}|\begin{array}{l} J\\ M^{\prime \prime } \end{array}\right)\left(\begin{array}{ll} J & k\\ M^{\prime \prime } & q \end{array}|\begin{array}{l} J\\ M^{\prime } \end{array}\right)\left(\begin{array}{ll} J & 1\\ M^{\prime } & p \end{array}|\begin{array}{l} J\\ M \end{array}\right)$$
105

Evaluating the contraction of CGCs and antisymmetric symbols graphically as shown in Figure 10 and inserting the result gives

$${\left(\frac{\partial^{3}\langle {\left\langle {\mathrm{JJ}}^{T}\rangle \right\rangle }_{Q}^{\left(K\right)}}{\partial \beta^{3}}\right)}_{\beta =0}\leftarrow J\left(J+1\right)\left(2J+1\right)\underset{k\mathrm{k̃}}{\sum }\left\langle J∥O^{\left(k\right)}∥J\right\rangle \left\langle J∥O^{\left(\mathrm{k̃}\right)}∥J\right\rangle \times \left\{\begin{array}{lll} J & J & 1\\ J & J & 1\\ k & \mathrm{k̃} & K \end{array}\right\}{\left(B^{\left(k\right)}⊗B^{\left(\mathrm{k̃}\right)}\right)}_{Q}^{\left(K\right)}$$
106

#### Combining the Parts

In order to proceed, we need explicit expressions for the 6*j* and 9*j* symbols occurring in eqs 104 and 106, which may be obtained from the analytical expressions listed in the sections “9.11. Tables of Algebraic Expressions for the 6*j* Symbols” and “10.11. Tables of Algebraic Formulas of the 9*j* Symbols” of Reference 49. The expressions for the first 6*j* symbol are

107
$$\left\{\begin{array}{lll} J & J & 1\\ 1 & 0 & J \end{array}\right\}={\left(-1\right)}^{2J+1}\frac{1}{\sqrt{3}}\frac{1}{\sqrt{2J+1}}$$

108
$$\left\{\begin{array}{lll} J & J & 1\\ 1 & 2 & J \end{array}\right\}={\left(-1\right)}^{2J}\delta \left(J\geq 1\right)\frac{1}{2\sqrt{30}}\sqrt{\frac{\left(2J+3\right)!}{\left(2J-2\right)!}}\frac{1}{J\left(J+1\right)\left(2J+1\right)}$$

­(The latter is exactly the 6*j* symbol we already needed for the second derivative/Bleaney’s theory, eq 93).

The two symbols

$$\left\{\begin{array}{lll} J & J & \mathrm{k̃}\\ K & k & J \end{array}\right\}\;\mathrm{and}\;\left\{\begin{array}{lll} J & J & 1\\ J & J & 1\\ k & \mathrm{k̃} & K \end{array}\right\}$$

vanish if |*k* – *k̃*| > *K*. Furthermore, they are symmetric under interchanging *k* and k̃. Therefore, we only need the following expressions

109
$$\left\{\begin{array}{lll} J & J & k\\ 0 & k & J \end{array}\right\}={\left(-1\right)}^{2J}\delta \left(J\geq 1/2\right)\sqrt{\frac{1}{\left(2k+1\right)\left(2J+1\right)}}$$

110
$$\left\{\begin{array}{lll} J & J & k\\ 2 & k & J \end{array}\right\}={\left(-1\right)}^{2J+1}\delta \left(J\geq 1/2\right)2k\left(k+1\right)\sqrt{\frac{\left(2k-2\right)!\left(2J-2\right)!}{\left(2k+3\right)!\left(2J+3\right)!}}\left[4J\left(J+1\right)-3k\left(k+1\right)+3\right]$$

111
$$\left\{\begin{array}{lll} J & J & k\\ 2 & k-2 & J \end{array}\right\}=\left\{\begin{array}{lll} J & J & k-2\\ 2 & k & J \end{array}\right\}={\left(-1\right)}^{2J}\delta \left(J\geq 1/2\right)2\sqrt{6}\sqrt{\frac{k\left(k-1\right)}{\left(2k+1\right)\left(2k-1\right)\left(2k-3\right)}}\frac{\left\langle J∥O^{\left(k\right)}∥J\right\rangle }{\left\langle J∥O^{\left(k-2\right)}∥J\right\rangle }\sqrt{\frac{\left(2J-2\right)!}{\left(2J+3\right)!}}$$

112
$$\left\{\begin{array}{lll} J & J & 1\\ J & J & 1\\ k & k & 0 \end{array}\right\}=\delta \left(J\geq 1/2\right)\frac{J\left(J+1\right)-\frac{1}{2}k\left(k+1\right)}{\sqrt{3}\sqrt{2k+1}J\left(J+1\right)\left(2J+1\right)}$$

113
$$\left\{\begin{array}{lll} J & J & 1\\ J & J & 1\\ k & k & 2 \end{array}\right\}=\delta \left(J\geq 1/2\right)\sqrt{\frac{2}{15}}k\left(k+1\right)\sqrt{\frac{\left(2k-2\right)!}{\left(2k+3\right)!}}\frac{2J\left(J+1\right)+\frac{1}{2}k\left(k+1\right)}{J\left(J+1\right)\left(2J+1\right)}$$

114
$$\left\{\begin{array}{lll} J & J & 1\\ J & J & 1\\ k & k-2 & 2 \end{array}\right\}=\left\{\begin{array}{lll} J & J & 1\\ J & J & 1\\ k-2 & k & 2 \end{array}\right\}=-\delta \left(J\geq 1/2\right)\frac{1}{\sqrt{5}}\sqrt{\frac{k\left(k-1\right)}{\left(2k+1\right)\left(2k-1\right)\left(2k-3\right)}}\frac{\left\langle J∥O^{\left(k\right)}∥J\right\rangle }{\left\langle J∥O^{\left(k-2\right)}∥J\right\rangle }\frac{1}{J\left(J+1\right)\left(2J+1\right)}$$

Combining all three parts and inserting these expressions, we obtain for the third derivative of the *K* = 0 part eq 27 of the main text. Since *k*, *k̃*, *K* are all even

115
$$\left(\begin{array}{ll} k & \mathrm{k̃}\\ q & \mathrm{q̃} \end{array}|\begin{array}{l} K\\ Q \end{array}\right)=\left(\begin{array}{ll} \mathrm{k̃} & k\\ \mathrm{q̃} & q \end{array}|\begin{array}{l} K\\ Q \end{array}\right)$$

which leads to

$${\left(B^{\left(k\right)}⊗B^{\left(\mathrm{k̃}\right)}\right)}_{Q}^{\left(K\right)}={\left(B^{\left(\mathrm{k̃}\right)}⊗B^{\left(k\right)}\right)}_{Q}^{\left(K\right)}$$
116

Using this fact, the sum of all contributions to the third derivative of the *K* = 2 part are equal to eq 28 of the main text.

## Notes

Derivation of numerical derivative equations, derivation of eqs 68 and 69, results on simultaneous scaling of *H* ^(0)^ and *T*, plots of relative error versus *h* and convergence plots for all investigated systems (PDF) (PDF)
Python script for obtaining the numerical prefactors in the Cartesian form of the tensor product of the **D**-tensor with itself (TXT)
