# Supplementary material for: Generalization of Bleaney’s Theory
Source: J Phys Chem A. 2026 Jun 18;130(26):5057–75. doi: 10.1021/acs.jpca.6c01791 (PMC13339661; doi:10.1021/acs.jpca.6c01791)
Supplement: Supplementary file 1 [file jp6c01791_si_001.pdf]

Supporting Information: Generalization of Bleaney's Theory

Lucas Lang,[*,†] Bryan Lauw,[†] and Letizia Fiorucci[‡]

†*Technische Universität Berlin, Institut für Chemie, Theoretische Chemie/Quantenchemie, Sekr. C7, Straße des 17. Juni 135, 10623 Berlin, Germany*

‡*Max-Planck-Institut für Kohlenforschung, Kaiser-Wilhelm-Platz 1, 45470 Mülheim an der Ruhr, Germany*

E-mail: lucas.lang@chem.tu-berlin.de

S1 Derivation of finite-difference equations for numerical derivatives

S1.1 Approach 1: Taylor expansions

Our goal is to evaluate the derivatives of the function $f(x)$ (where x is a real number) at $x = 0$ numerically. f is not necessarily single-valued. In our case, f is a matrix-valued function (the angular momentum dyadic). The function can be expanded in the vicinity of this point via the Taylor expansion

$$f(x) = f(0) + f'(0)x + \frac{1}{2}f''(0)x^2 + \cdots = \sum_{n=0}^{\infty} \frac{1}{n!} f^{(n)}(0)x^n \tag{S1}$$

Next, assume that h is a small positive number and that the function f is evaluated at the values $n_i h$, where there are a total of M different (positive and/or negative) multiples of h with prefactor n_i. Taking a linear combination of these M function values with coefficients a_i, one obtains

$$\begin{aligned}\sum_{i=1}^{M} a_i f(n_i h) &= \sum_i a_i \left[\sum_{n=0}^{M-1} \frac{1}{n!} f^{(n)}(0)(n_i h)^n + \mathcal{O}(h^M)\right] \\ &= \sum_{n=0}^{M-1} c_n h^n f^{(n)}(0) + \mathcal{O}(h^M)\end{aligned} \tag{S2}$$

where the vectors $\mathbf{c}$ and $\mathbf{a}$, containing the coefficients c_n and a_i, are related via

$$\mathbf{c} = \mathbf{C}\mathbf{a} \tag{S3}$$

Here, the matrix $\mathbf{C}$ is given by

$$C_{ni} = \frac{n_i^n}{n!} \tag{S4}$$

This matrix is closely related to a Vandermonde matrix, and is invertible if the values n_i are unique (see section S1.3 below).

Now suppose that we are interested in the jth derivative of f at $x = 0$. Then the coefficients a_i need to be chosen such that the coefficient c_n is 1 for $n = j$ and 0 for all other derivatives, i.e., $c_n = \delta_{nj}$. Call the coefficients a_i for which this is the case $a_i^{(j)}$. From Eq. (S3),

$$a_i^{(j)} = \sum_n C_{in}^{-1} \delta_{nj} = C_{ij}^{-1} \tag{S5}$$

Inserting this into Eq. (S2), one obtains

$$\sum_{i=1}^{M} C_{ij}^{-1} f(n_i h) = h^j f^{(j)}(0) + \mathcal{O}(h^M) \tag{S6}$$

or

$$f^{(j)}(0) = \frac{1}{h^j} \sum_{i=1}^{M} C_{ij}^{-1} f(n_i h) + \mathcal{O}(h^{M-j}) \tag{S7}$$

As a concrete example, consider the five-point stencil where the function f is evaluated at $0, \pm h, \pm 2h$. Then for the first two derivatives of f at $x = 0$, Eq. (S7) becomes

$$f'(0) \approx \frac{1}{12h} \left[f(-2h) - 8f(-h) + 8f(h) - f(2h) \right] \tag{S8}$$

$$f''(0) \approx \frac{1}{12h^2} \left[-f(-2h) + 16f(-h) - 30f(0) + 16f(h) - f(2h) \right] \tag{S9}$$

S1.2 Approach 2: Fitting to a polynomial

An alternative but closely related point of view involves least-squares fitting of the function f to the Mth order polynomial $\sum_{n=0}^{M-1} \frac{1}{n!} f^{(n)}(0) x^n$. From this point of view, the derivatives are the fitting parameters. The data we use for fitting are the function evaluations at the points $n_i h$:

$$f(n_i h) \stackrel{!}{=} \sum_{n=0}^{M-1} \frac{1}{n!} f^{(n)}(0) n_i^n h^n = \sum_{n=0}^{M-1} A_{in} f^{(n)}(0) \tag{S10}$$

with the matrix $\mathbf{A}$ given by

$$A_{in} = \frac{n_i^n}{n!} h^n = C_{ni} h^n \tag{S11}$$

In general for a linear model, the parameters that minimize the sum of squared deviations between the "model values" and the "true values" $f(n_i h)$ (least-squares fitting) are given by

$$f^{(j)}(0) = \sum_{i=1}^{M} A^{+}_{ji} f(n_i h) \tag{S12}$$

where $\mathbf{A}^+$ is the Moore-Penrose pseudoinverse of $\mathbf{A}$. Since $\mathbf{C}$ is invertible, also $\mathbf{A}$ is invertible and

$$A^{+}_{ml} = A^{-1}_{ml} = \frac{1}{h^m} C^{-1}_{lm} \tag{S13}$$

Invertibility of $\mathbf{A}$ means that the Mth order polynomial can *exactly* fit the data and that the sum of squared deviations is zero. Eqs. (S12) and (S13) can be combined to give the "optimal" derivatives

$$f^{(j)}(0) = \frac{1}{h^j} \sum_{i=1}^{M} C^{-1}_{ij} f(n_i h) \tag{S14}$$

One can see that this equation is identical to the one we obtained in the previous section via a slightly different route. The disadvantage of this second derivation is that the size of the error in the derivative (which, as we have seen before, is of order $\mathcal{O}(h^{M-j})$) is unknown.

S1.3 Invertibility of the matrices

The Vandermonde matrix $\mathbf{V}$ of the numbers n_i is given by

$$V_{ij} = n_i^j \tag{S15}$$

The determinant of a Vandermonde matrix is equal to the product of all unique differences between two n_i values, which shows that the determinant must be nonzero (and $\mathbf{V}$ invertible) if all n_i are unique, because then all the differences between different n_i are non-zero.

The matrix $\mathbf{C}$ introduced above is related to the Vandermonde matrix via

$$C_{ni} = \frac{V_{in}}{n!} \tag{S16}$$

Given this relationship, one can easily check that

$$C^{-1}_{jm} = m! V^{-1}_{mj} \tag{S17}$$

defines the inverse of $\mathbf{C}$ (i.e., it gives the identity matrix when multiplied with $\mathbf{C}$ from the left or from the right).

Alternatively, one can notice that $\mathbf{C}$ is defined by multiplying $\mathbf{V}^{\mathrm{T}}$ with a diagonal matrix having the values $1/n!$ on its diagonal. Since the determinant of the product of two matrices is the product of the two determinants,

$$\det \mathbf{C} = \left(\prod_n \frac{1}{n!} \right) \det \mathbf{V} \tag{S18}$$

which also shows that the invertibility of $\mathbf{V}$ implies the invertibility of $\mathbf{C}$.

S2 Third-order expansion of contact and pseudocontact shifts by Martin and Autschbach

Our definition of the spin dyadic is related to the $\mathbf{Z}$-matrix of Autschbach and co-workers[1] via

$$\langle\langle S_i S_j \rangle\rangle = -\beta Z_{ij} \tag{S19}$$

i.e., the hyperfine shielding tensor and isotropic shielding are given by

$$\boldsymbol{\sigma} = -\frac{\mu_{\mathrm{B}}\beta}{\gamma} \mathbf{g}\mathbf{Z}\mathbf{A}^{\mathrm{T}} \tag{S20}$$

$$\sigma_{\text{iso}} = \frac{1}{3}\,\text{tr}(\boldsymbol{\sigma}) = -\frac{\mu_{\text{B}}\beta}{3\gamma}\,\text{tr}(\mathbf{g}\mathbf{Z}\mathbf{A}^{\text{T}}) \tag{S21}$$

Like Martin and Autschbach,[1] we now assume that the system has axial symmetry, such that $\mathbf{g}$ and $\mathbf{Z}$ are diagonal and the perpendicular x and y components ($\perp$) are equal and differ from the parallel z component ($\|$). Under this assumption,

$$\text{tr}(\mathbf{g}\mathbf{Z}\mathbf{A}^{\text{T}}) = \sum_k g_k Z_k A_{kk} = g_\perp Z_\perp (A_{xx} + A_{yy}) + g_\| Z_\| A_{zz} \tag{S22}$$

Following Martin and Autschbach, we now define

$$A_{\text{iso}} = \frac{1}{3}\,\text{tr}(\mathbf{A}) = \frac{1}{3}(A_{xx} + A_{yy} + A_{zz}) \tag{S23}$$

$$\Delta A = \frac{1}{3}\left[A_{zz} - \frac{1}{2}(A_{xx} + A_{yy})\right] \tag{S24}$$

which implies that

$$A_{xx} + A_{yy} = 2(A_{\text{iso}} - \Delta A) \tag{S25}$$

$$A_{zz} = A_{\text{iso}} + 2\Delta A \tag{S26}$$

Inserting these, one obtains

$$\text{tr}(\mathbf{g}\mathbf{Z}\mathbf{A}^{\text{T}}) = A_{\text{iso}}(2g_\perp Z_\perp + g_\| Z_\|) + \Delta A(2g_\| Z_\| - 2g_\perp Z_\perp) \tag{S27}$$

In complete analogy to the $\mathbf{A}$-tensor, we now define g_{iso} and Δg such that we can write

$$g_\| = g_{\text{iso}} + 2\Delta g \tag{S28}$$

$$g_\perp = g_{\text{iso}} - \Delta g \tag{S29}$$

Inserting these, we obtain

$$\begin{aligned}\mathrm{tr}(\mathbf{g}\mathbf{Z}\mathbf{A}^{\mathrm{T}}) = A_{\mathrm{iso}}\left[g_{\mathrm{iso}}(2Z_{\perp} + Z_{\parallel}) + 2\Delta g(Z_{\parallel} - Z_{\perp})\right] \\ + \Delta A\left[2g_{\mathrm{iso}}(Z_{\parallel} - Z_{\perp}) + 2\Delta g(2Z_{\parallel} + Z_{\perp})\right]\end{aligned} \tag{S30}$$

Martin and Autschbach called the first part (depending on A_{iso}) the contact shielding $\sigma_{\mathrm{iso}}^{\mathrm{c}}$ and the second part (depending on ΔA) the pseudocontact shielding $\sigma_{\mathrm{iso}}^{\mathrm{pc}}$. In this section, we stay with their nomenclature, although we would consider shielding contributions due to the orbital/SOC contribution to the **A**-tensor (which has an isotropic component) as pseudocontact in character.

From our expansion of the spin dyadic (Eq. (67) of the main manuscript), the expansion of the **Z**-matrix up to order β^2 is given by

$$\begin{aligned}\mathbf{Z} = &\frac{S(S+1)}{3}\mathbf{I} - \frac{S(S+1)(2S-1)(2S+3)}{30}\mathbf{D}\beta \\ &- \frac{S(S+1)(2S-1)(2S+3)}{15}\left[\frac{1}{12}\,\mathrm{tr}(\mathbf{D}^2)\mathbf{I} - \frac{(S+2)(S-1)}{7}(\mathbf{D}^2)^{\mathrm{aniso}}\right]\beta^2\end{aligned} \tag{S31}$$

Since we assumed an axial system, the traceless **D**-tensor can be written as

$$\mathbf{D} = D\begin{pmatrix} -1/3 & 0 & 0 \\ 0 & -1/3 & 0 \\ 0 & 0 & 2/3 \end{pmatrix} \tag{S32}$$

from which one obtains

$$\mathrm{tr}(\mathbf{D}^2) = \frac{2D^2}{3} \tag{S33}$$

$$(\mathbf{D}^2)^{\mathrm{aniso}} = D^2\begin{pmatrix} -1/9 & 0 & 0 \\ 0 & -1/9 & 0 \\ 0 & 0 & 2/9 \end{pmatrix} \tag{S34}$$

Hence,

$$Z_\perp = \frac{S(S+1)}{3} + \frac{D\beta}{3}\frac{S(S+1)(2S-1)(2S+3)}{30} - D^2\beta^2\frac{S(S+1)(2S-1)(2S+3)}{135}\left[\frac{1}{2} + \frac{(S+2)(S-1)}{7}\right] \tag{S35}$$

$$Z_\parallel = \frac{S(S+1)}{3} - \frac{2D\beta}{3}\frac{S(S+1)(2S-1)(2S+3)}{30} - D^2\beta^2\frac{S(S+1)(2S-1)(2S+3)}{135}\left[\frac{1}{2} - \frac{2(S+2)(S-1)}{7}\right] \tag{S36}$$

leading to the following combinations:

$$2Z_\perp + Z_\parallel = S(S+1) - D^2\beta^2\frac{S(S+1)(2S-1)(2S+3)}{90} \tag{S37}$$

$$Z_\parallel - Z_\perp = -D\beta\frac{S(S+1)(2S-1)(2S+3)}{30} + D^2\beta^2\frac{S(S+1)(2S-1)(2S+3)(S+2)(S-1)}{315} \tag{S38}$$

$$2Z_\parallel + Z_\perp = S(S+1) - D\beta\frac{S(S+1)(2S-1)(2S+3)}{30} - D^2\beta^2\frac{S(S+1)(2S-1)(2S+3)}{45}\left[\frac{1}{2} - \frac{(S+2)(S-1)}{7}\right] \tag{S39}$$

Inserting these combinations yields the final equations for the contact and pseudocontact isotropic shieldings given in Eqs. (68) and (69) of the main manuscript.

S3 Simultaneous scaling of the field-free Hamiltonian and the temperature

The first few derivatives appearing as coefficients in the Taylor expansion of the exact dyadic around $\beta = 0$,

$$\langle\langle \mathbf{J}\mathbf{J}^{\mathrm{T}} \rangle\rangle = \left(\frac{\partial \langle\langle \mathbf{J}\mathbf{J}^{\mathrm{T}} \rangle\rangle}{\partial \beta} \right)_{\beta=0} \beta + \frac{1}{2} \left(\frac{\partial^2 \langle\langle \mathbf{J}\mathbf{J}^{\mathrm{T}} \rangle\rangle}{\partial \beta^2} \right)_{\beta=0} \beta^2 + \ldots \tag{S40}$$

are given by Eqs. (15)–(17) of the main manuscript. One can see that the nth derivative of the dyadic with respect to β contains the $(n-1)$th power of the field-free Hamiltonian $H^{(0)}$. This means that, if the field-free Hamiltonian is scaled by a real number $\alpha \neq 0$, i.e.,

$$H^{(0)\prime} = \alpha H^{(0)} \tag{S41}$$

then

$$\left(\frac{\partial^n \langle\langle \mathbf{J}\mathbf{J}^{\mathrm{T}} \rangle\rangle}{\partial \beta^n} \right)'_{\beta=0} = \alpha^{n-1} \left(\frac{\partial^n \langle\langle \mathbf{J}\mathbf{J}^{\mathrm{T}} \rangle\rangle}{\partial \beta^n} \right)_{\beta=0} \tag{S42}$$

If at the same time one *inversely* scales β, i.e.,

$$\beta' = \frac{1}{\alpha} \beta \tag{S43}$$

then we can conclude from the Taylor expansion that

$$\langle\langle \mathbf{J}\mathbf{J}^{\mathrm{T}} \rangle\rangle' = \frac{1}{\alpha} \langle\langle \mathbf{J}\mathbf{J}^{\mathrm{T}} \rangle\rangle \tag{S44}$$

Importantly, this equation is not only true for the exact dyadic (i.e., keeping an infinite number of terms in the Taylor expansion), but also for *any truncation at finite order in β*. Therefore, the *relative error* of such a truncated approximation of the dyadic will be

unchanged under simultaneous scaling of $H^{(0)}$ and β in the way just described:

$$\frac{||\langle\langle \mathbf{J}\mathbf{J}^{\mathrm{T}}\rangle\rangle'^{\mathrm{approx}} - \langle\langle \mathbf{J}\mathbf{J}^{\mathrm{T}}\rangle\rangle'||}{||\langle\langle \mathbf{J}\mathbf{J}^{\mathrm{T}}\rangle\rangle'||} = \frac{||\langle\langle \mathbf{J}\mathbf{J}^{\mathrm{T}}\rangle\rangle^{\mathrm{approx}} - \langle\langle \mathbf{J}\mathbf{J}^{\mathrm{T}}\rangle\rangle||}{||\langle\langle \mathbf{J}\mathbf{J}^{\mathrm{T}}\rangle\rangle||} \tag{S45}$$

For the ZFS Hamiltonian (Eq. (53) of the main manuscript), $H^{(0)}$ is proportional to the axial ZFS parameter D. Therefore, when D and T are scaled simultaneously such that $D/k_{\mathrm{B}}T$ stays constant, the relative error will remain unchanged.

S4 Convergence plots for all test systems

S4.1 Transition-metal cases

S4.1.1 $S = 1$

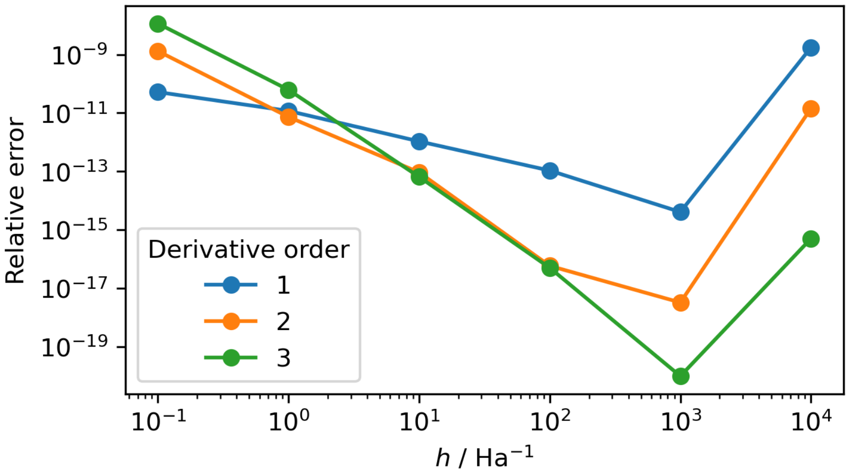

Figure S1: Relative error in the first three numerical derivatives of the spin dyadic as a function of the finite difference h. TM case with $S = 1$, $E/D = 0$ and $D = 6$ cm^{-1}.

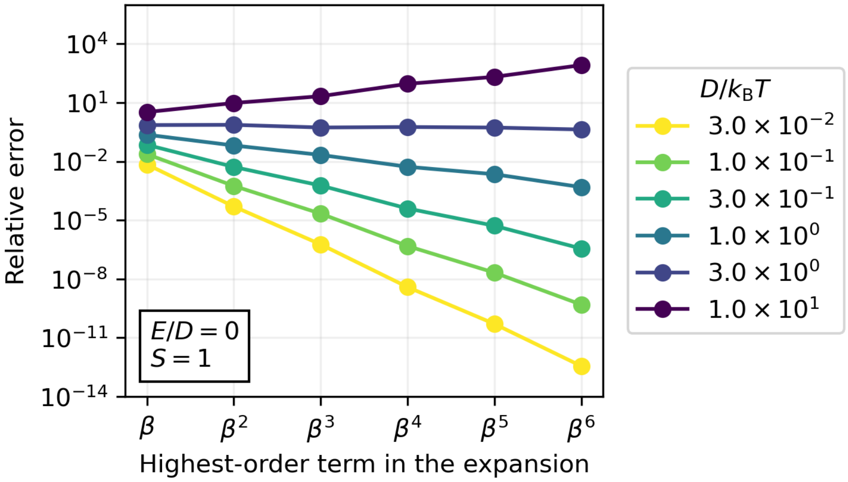

Figure S2: Relative error in the dyadic of a TM complex with $S = 1$, $E/D = 0$ and positive D when it is truncated at different orders in β and evaluated at different $D/k_{\mathrm{B}}T$.

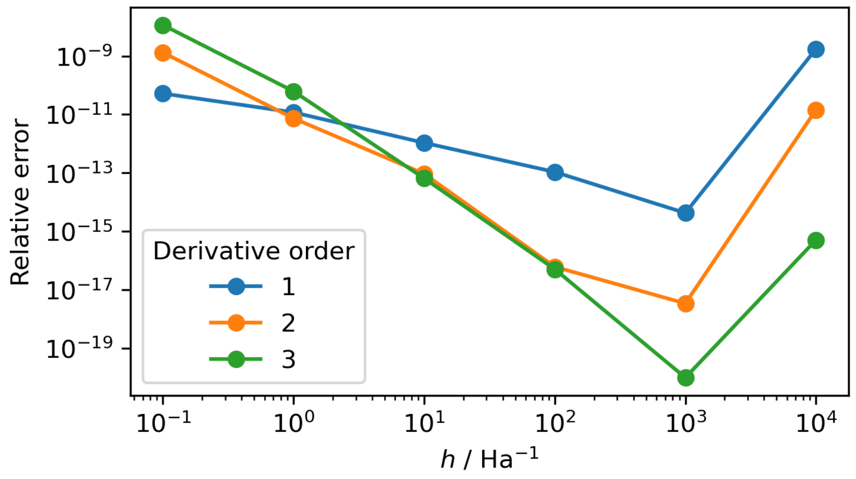

Figure S3: Relative error in the first three numerical derivatives of the spin dyadic as a function of the finite difference h. TM case with $S = 1$, $E/D = 0$ and $D = -6$ cm^{-1}.

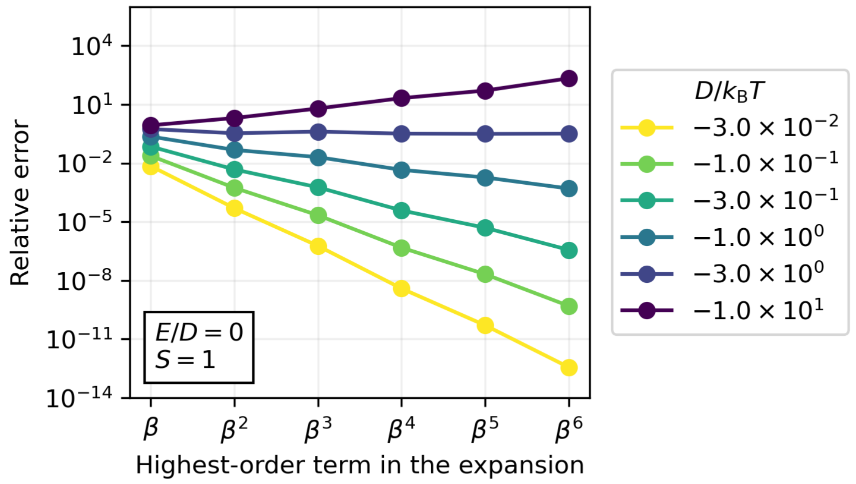

Figure S4: Relative error in the dyadic of a TM complex with $S = 1$, $E/D = 0$ and negative D when it is truncated at different orders in β and evaluated at different $D/k_\mathrm{B}T$.

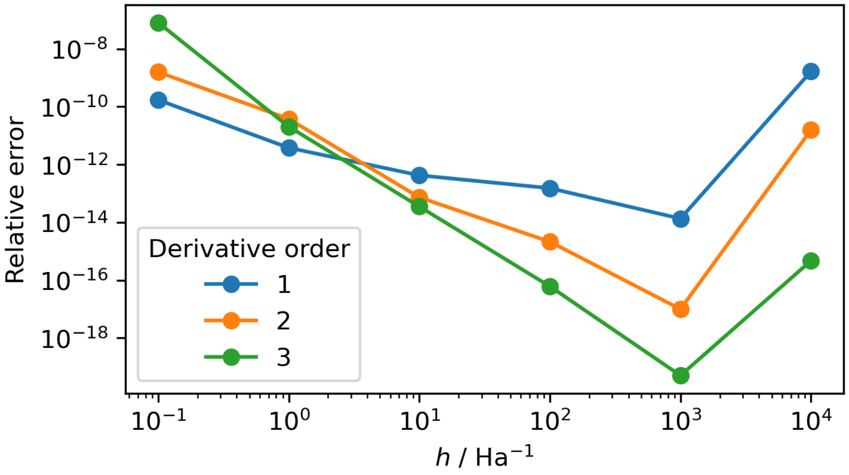

Figure S5: Relative error in the first three numerical derivatives of the spin dyadic as a function of the finite difference h. TM case with $S = 1$, $E/D = 1/6$ and $D = 6$ cm^{-1}.

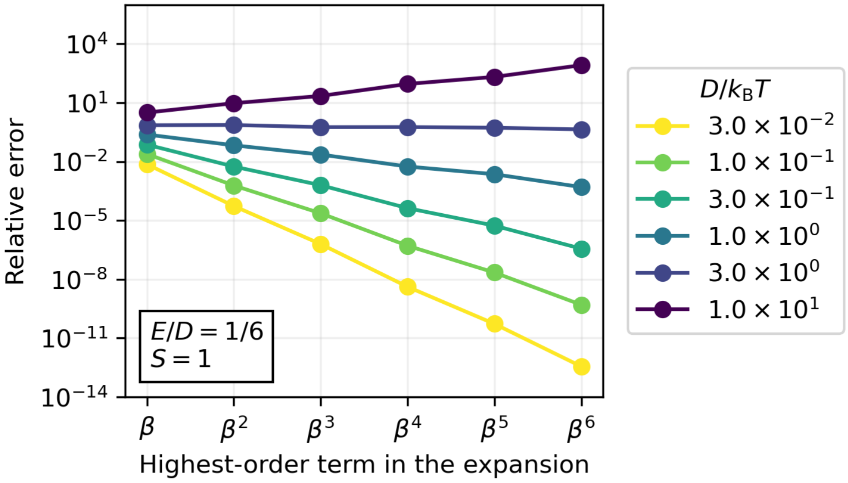

Figure S6: Relative error in the dyadic of a TM complex with $S = 1$, $E/D = 1/6$ and positive D when it is truncated at different orders in β and evaluated at different $D/k_\mathrm{B}T$.

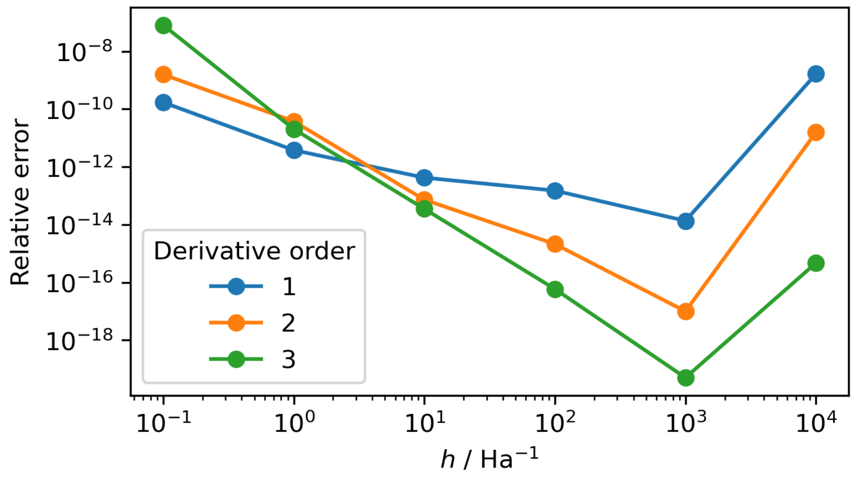

Figure S7: Relative error in the first three numerical derivatives of the spin dyadic as a function of the finite difference h. TM case with $S = 1$, $E/D = 1/6$ and $D = -6$ cm^{-1}.

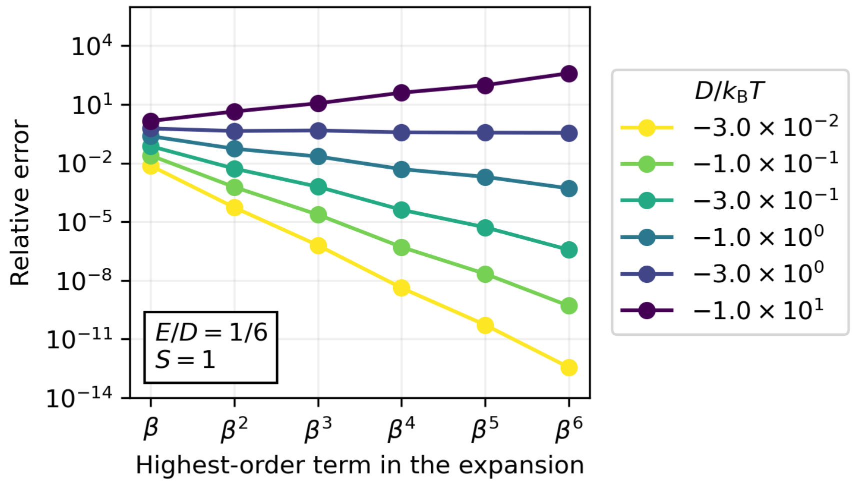

Figure S8: Relative error in the dyadic of a TM complex with $S = 1$, $E/D = 1/6$ and negative D when it is truncated at different orders in β and evaluated at different $D/k_\mathrm{B}T$.

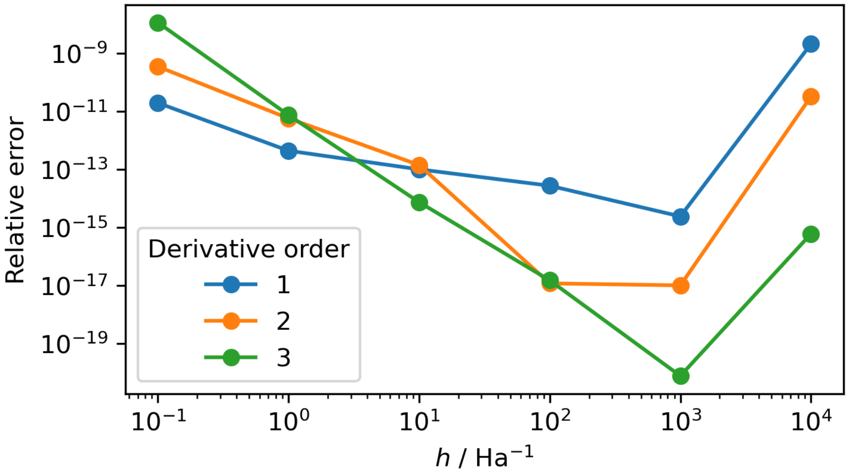

Figure S9: Relative error in the first three numerical derivatives of the spin dyadic as a function of the finite difference h. TM case with $S = 1$, $E/D = 1/3$ and $D = 6$ cm^{-1}.

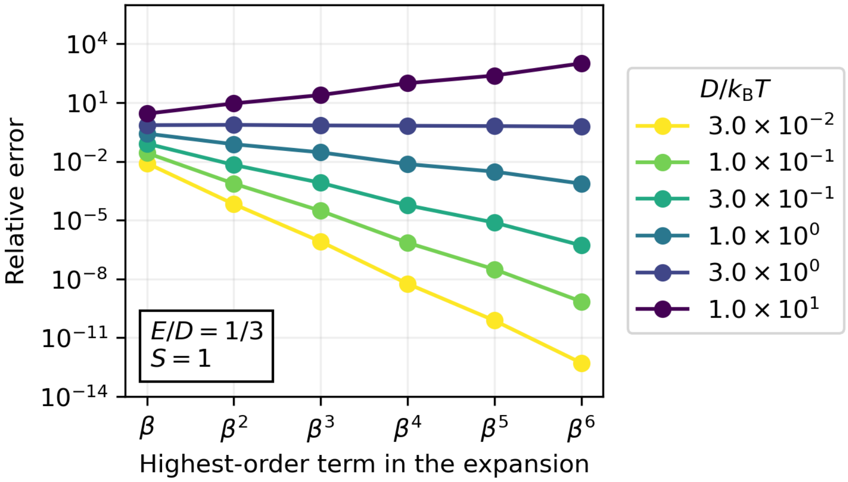

Figure S10: Relative error in the dyadic of a TM complex with $S = 1$, $E/D = 1/3$ and positive D when it is truncated at different orders in β and evaluated at different $D/k_{\mathrm{B}}T$.

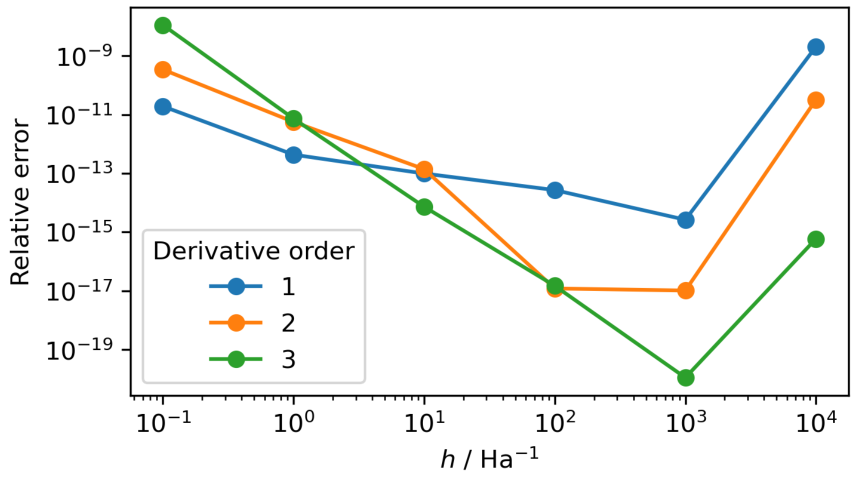

Figure S11: Relative error in the first three numerical derivatives of the spin dyadic as a function of the finite difference h. TM case with $S = 1$, $E/D = 1/3$ and $D = -6$ cm^{-1}.

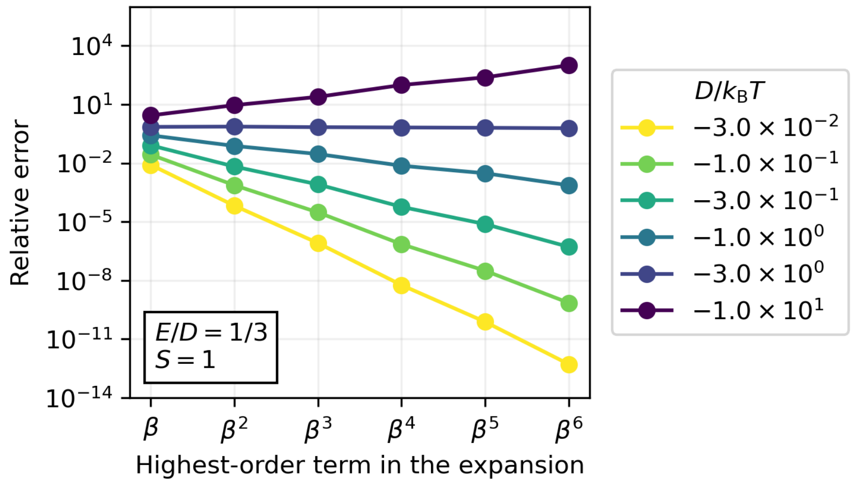

Figure S12: Relative error in the dyadic of a TM complex with $S = 1$, $E/D = 1/3$ and negative D when it is truncated at different orders in β and evaluated at different $D/k_\mathrm{B}T$.

S4.1.2 $S = 3/2$

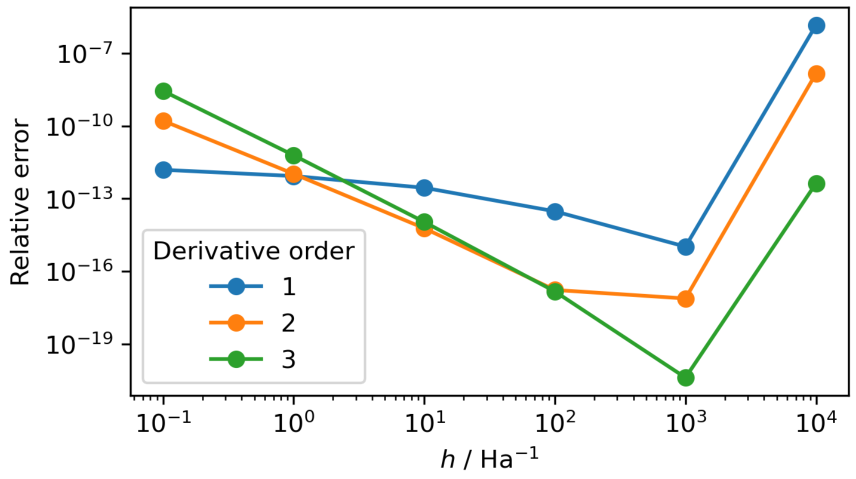

Figure S13: Relative error in the first three numerical derivatives of the spin dyadic as a function of the finite difference h. TM case with $S = 3/2$, $E/D = 0$ and $D = 6$ cm^{-1}.

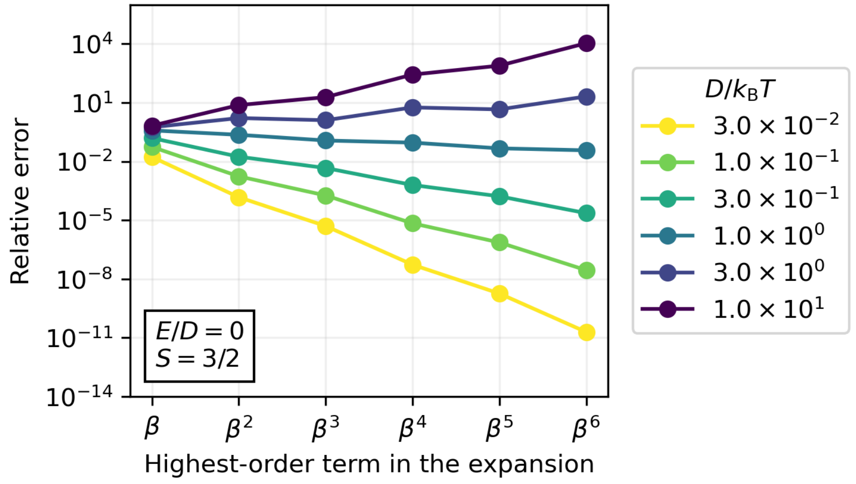

Figure S14: Relative error in the dyadic of a TM complex with $S = 3/2$, $E/D = 0$ and positive D when it is truncated at different orders in β and evaluated at different $D/k_{\mathrm{B}}T$.

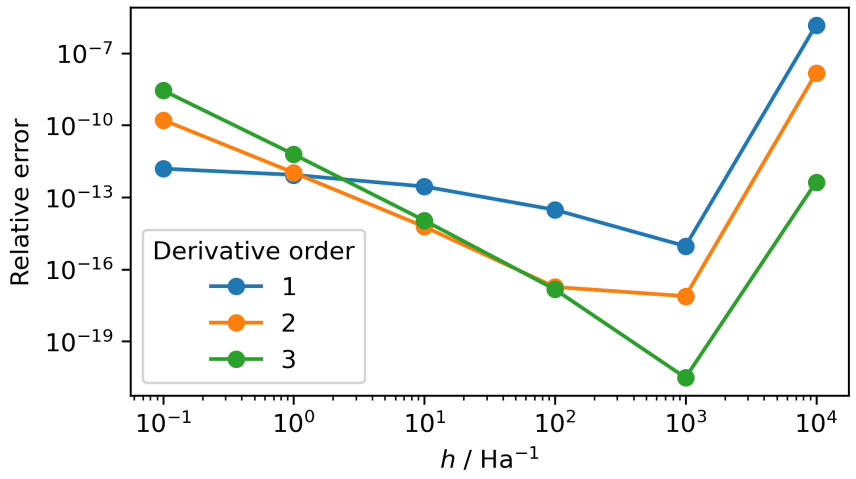

Figure S15: Relative error in the first three numerical derivatives of the spin dyadic as a function of the finite difference h. TM case with $S = 3/2$, $E/D = 0$ and $D = -6\ \mathrm{cm}^{-1}$.

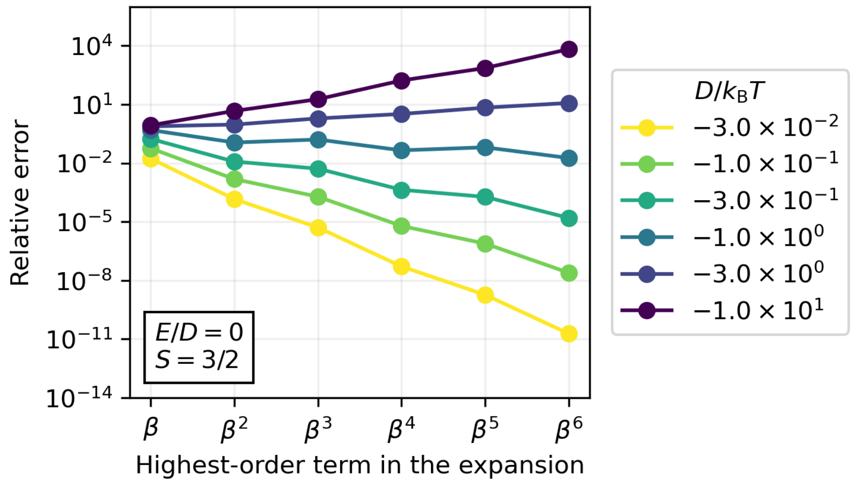

Figure S16: Relative error in the dyadic of a TM complex with $S = 3/2$, $E/D = 0$ and negative D when it is truncated at different orders in β and evaluated at different $D/k_{\mathrm{B}}T$.

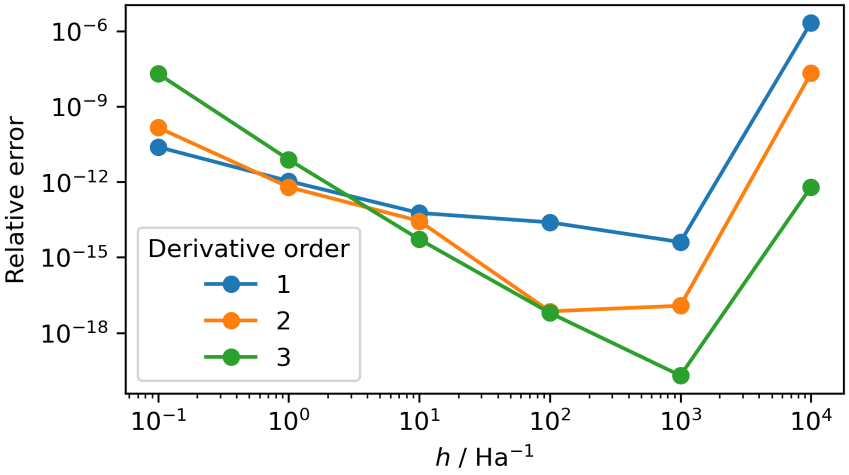

Figure S17: Relative error in the first three numerical derivatives of the spin dyadic as a function of the finite difference h. TM case with $S = 3/2$, $E/D = 1/6$ and $D = 6\ \mathrm{cm}^{-1}$.

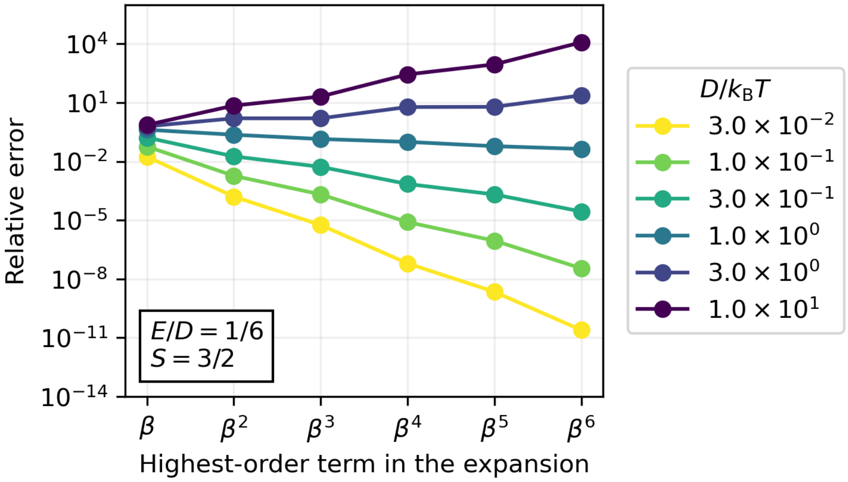

Figure S18: Relative error in the dyadic of a TM complex with $S = 3/2$, $E/D = 1/6$ and positive D when it is truncated at different orders in β and evaluated at different $D/k_\mathrm{B}T$.

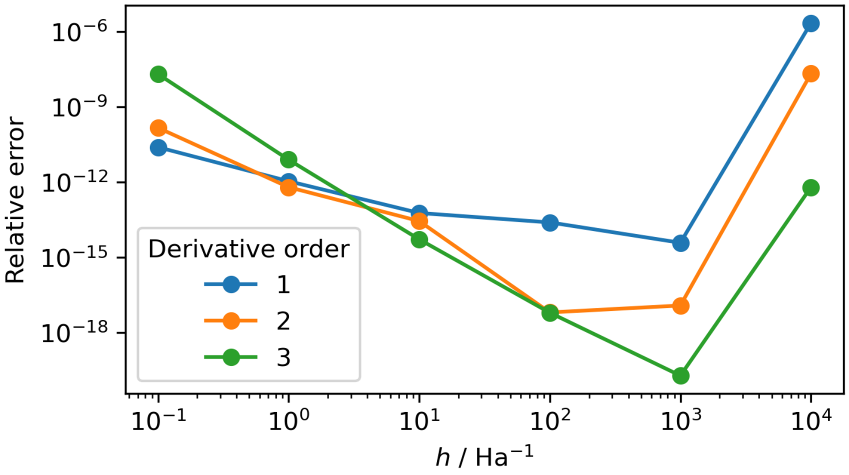

Figure S19: Relative error in the first three numerical derivatives of the spin dyadic as a function of the finite difference h. TM case with $S = 3/2$, $E/D = 1/6$ and $D = -6\ \mathrm{cm}^{-1}$.

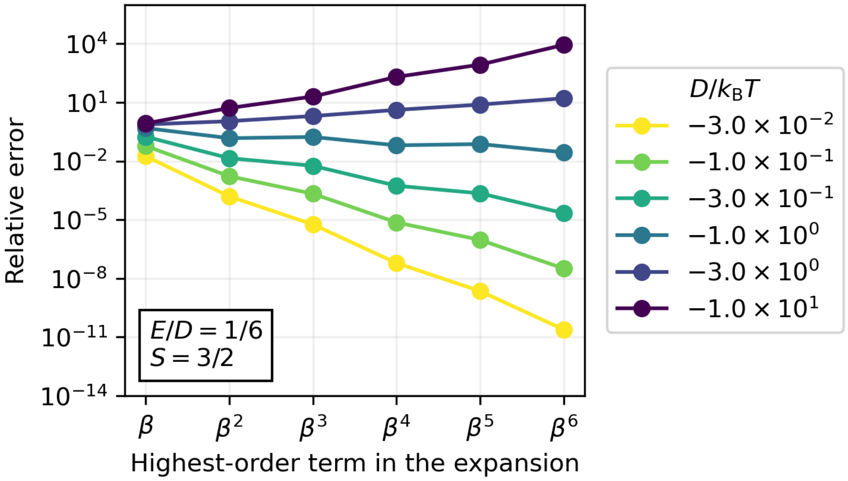

Figure S20: Relative error in the dyadic of a TM complex with $S = 3/2$, $E/D = 1/6$ and negative D when it is truncated at different orders in β and evaluated at different $D/k_\mathrm{B}T$.

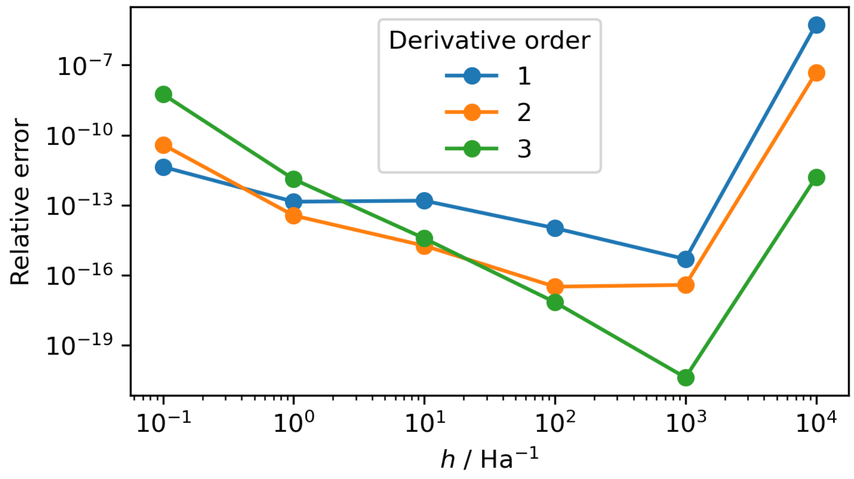

Figure S21: Relative error in the first three numerical derivatives of the spin dyadic as a function of the finite difference h. TM case with $S = 3/2$, $E/D = 1/3$ and $D = 6$ cm^{-1}.

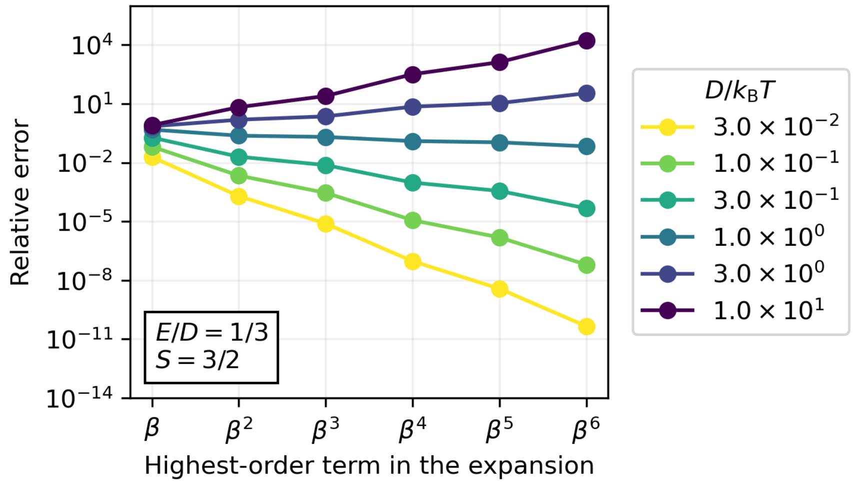

Figure S22: Relative error in the dyadic of a TM complex with $S = 3/2$, $E/D = 1/3$ and positive D when it is truncated at different orders in β and evaluated at different D/k_BT.

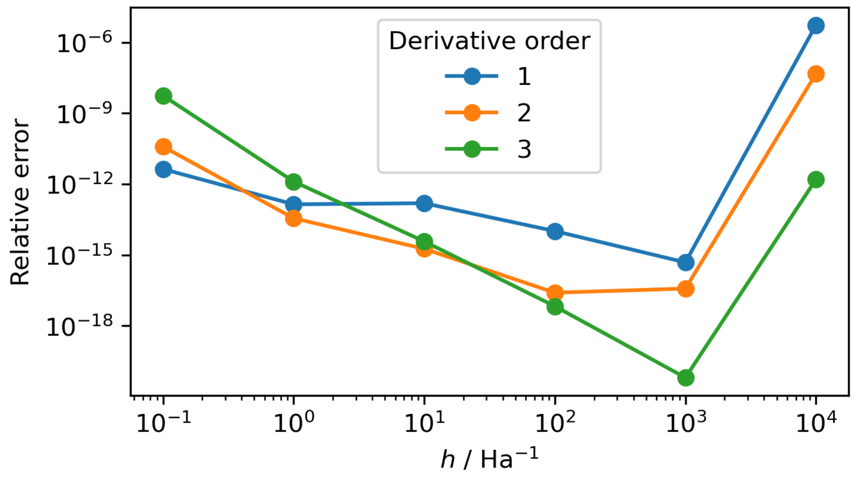

Figure S23: Relative error in the first three numerical derivatives of the spin dyadic as a function of the finite difference h. TM case with $S = 3/2$, $E/D = 1/3$ and $D = -6$ cm^{-1}.

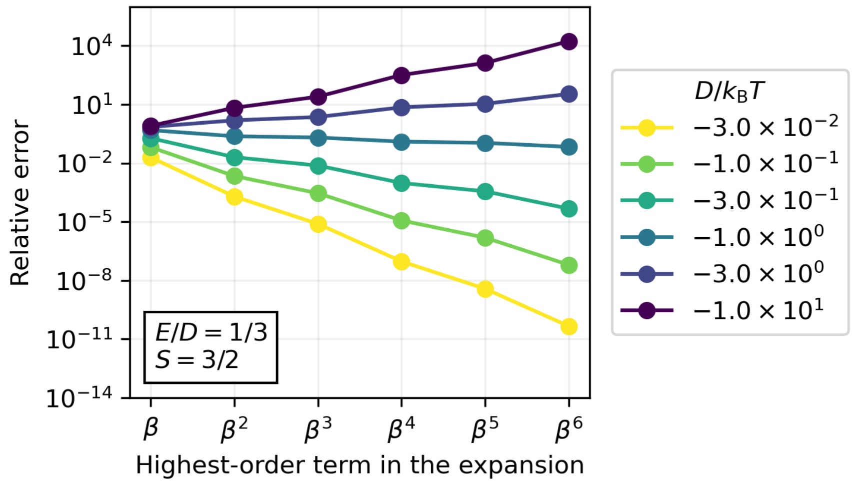

Figure S24: Relative error in the dyadic of a TM complex with $S = 3/2$, $E/D = 1/3$ and negative D when it is truncated at different orders in β and evaluated at different $D/k_\mathrm{B}T$.

S4.1.3 $S = 2$

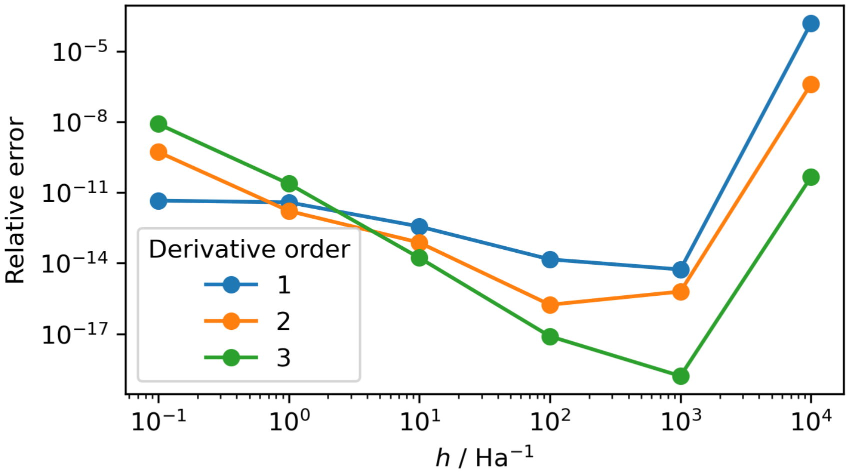

Figure S25: Relative error in the first three numerical derivatives of the spin dyadic as a function of the finite difference h. TM case with $S = 2$, $E/D = 0$ and $D = 6$ cm^{-1}.

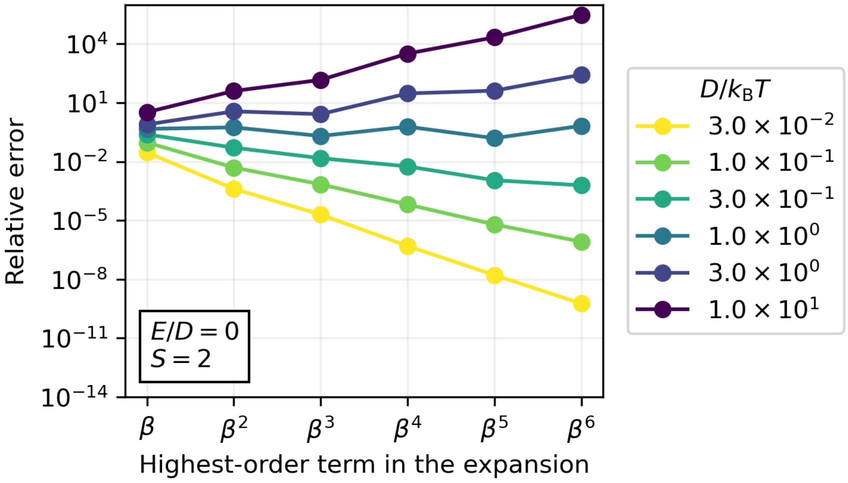

Figure S26: Relative error in the dyadic of a TM complex with $S = 2$, $E/D = 0$ and positive D when it is truncated at different orders in β and evaluated at different $D/k_\mathrm{B}T$.

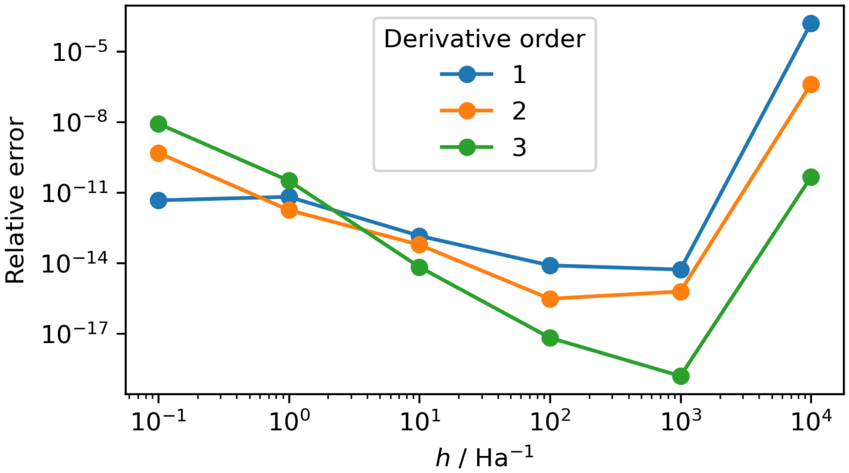

Figure S27: Relative error in the first three numerical derivatives of the spin dyadic as a function of the finite difference h. TM case with $S = 2$, $E/D = 0$ and $D = -6$ cm^{-1}.

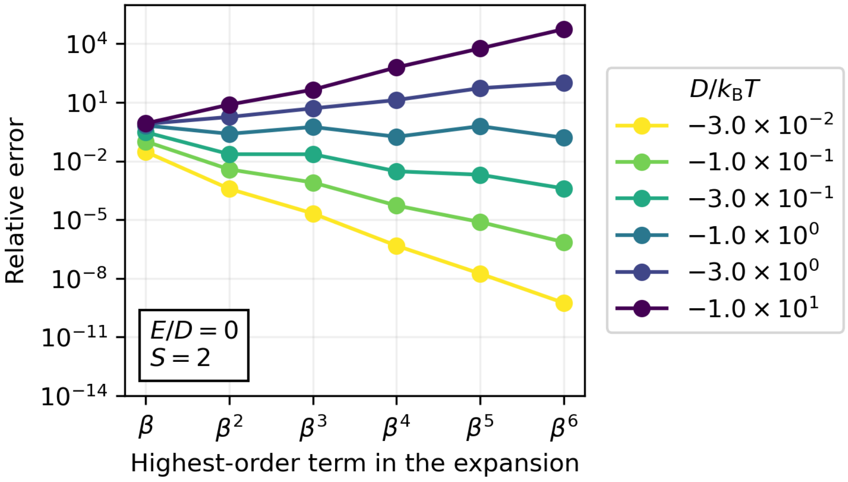

Figure S28: Relative error in the dyadic of a TM complex with $S = 2$, $E/D = 0$ and negative D when it is truncated at different orders in β and evaluated at different $D/k_{\mathrm{B}}T$.

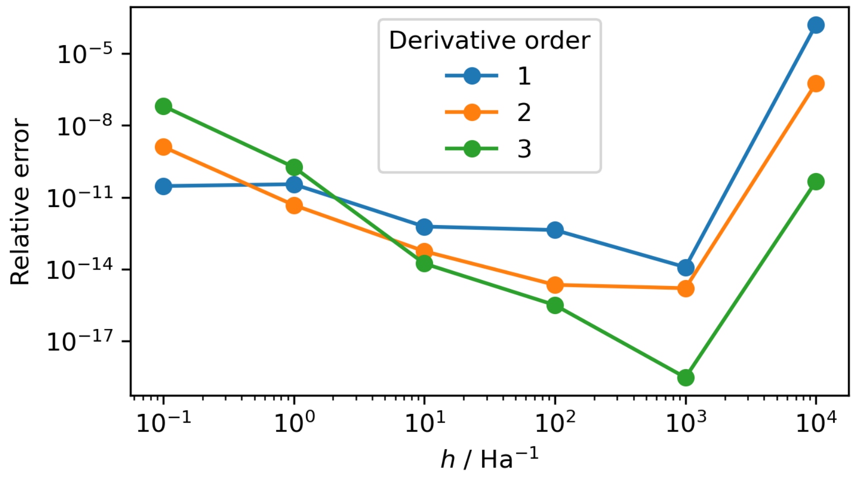

Figure S29: Relative error in the first three numerical derivatives of the spin dyadic as a function of the finite difference h. TM case with $S = 2$, $E/D = 1/6$ and $D = 6\ \text{cm}^{-1}$.

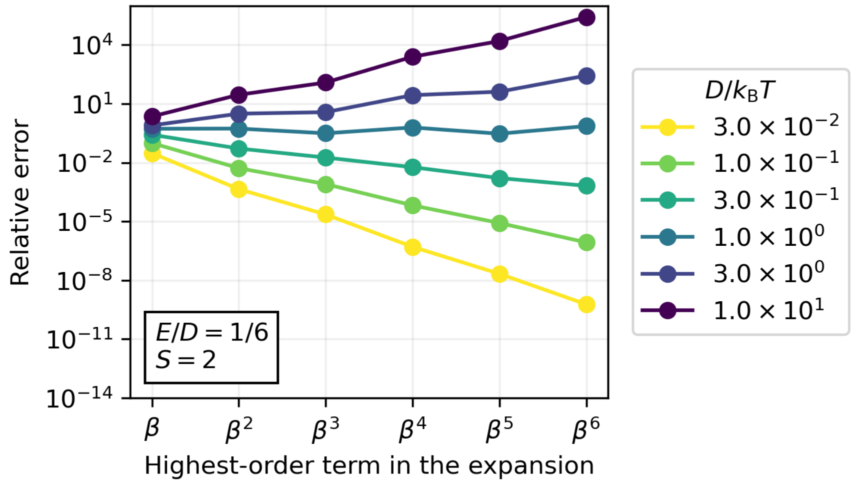

Figure S30: Relative error in the dyadic of a TM complex with $S = 2$, $E/D = 1/6$ and positive D when it is truncated at different orders in β and evaluated at different $D/k_\mathrm{B}T$.

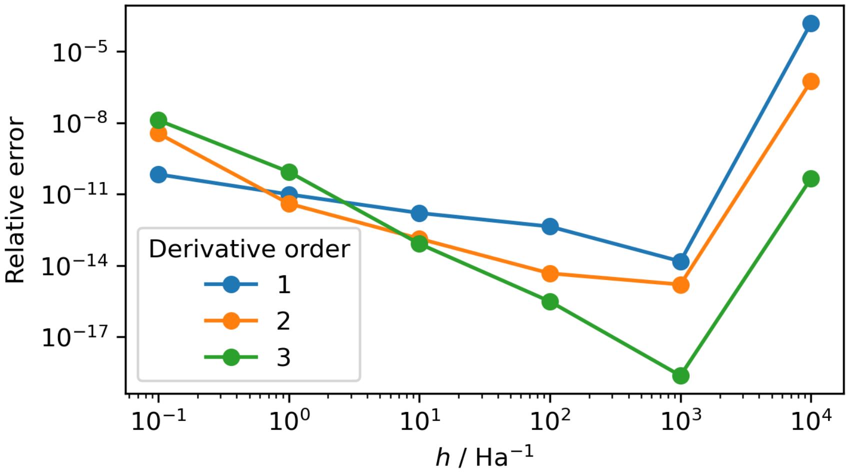

Figure S31: Relative error in the first three numerical derivatives of the spin dyadic as a function of the finite difference h. TM case with $S = 2$, $E/D = 1/6$ and $D = -6\ \mathrm{cm}^{-1}$.

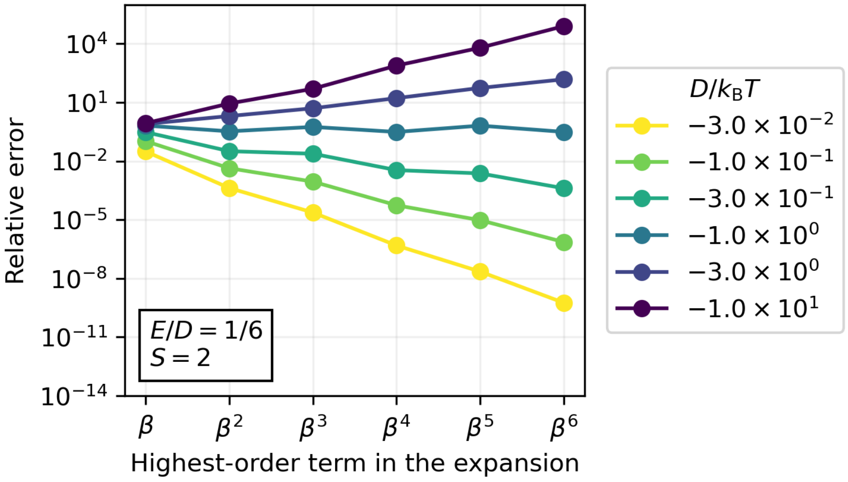

Figure S32: Relative error in the dyadic of a TM complex with $S = 2$, $E/D = 1/6$ and negative D when it is truncated at different orders in β and evaluated at different $D/k_\mathrm{B}T$.

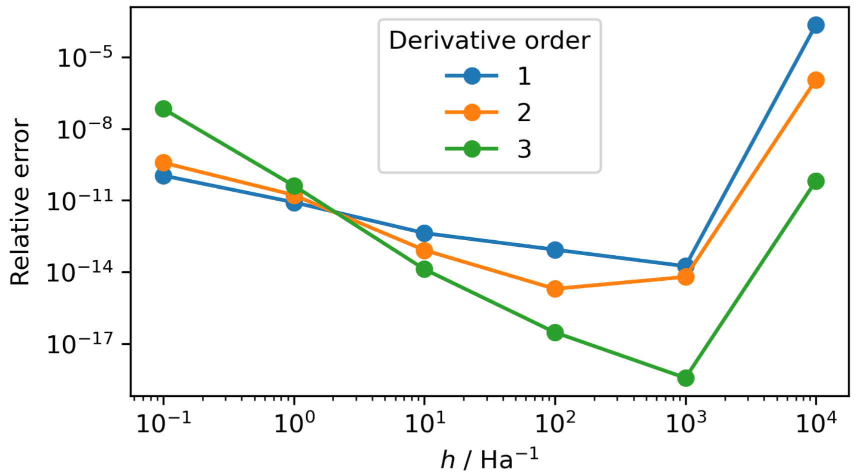

Figure S33: Relative error in the first three numerical derivatives of the spin dyadic as a function of the finite difference h. TM case with $S = 2$, $E/D = 1/3$ and $D = 6\ \mathrm{cm}^{-1}$.

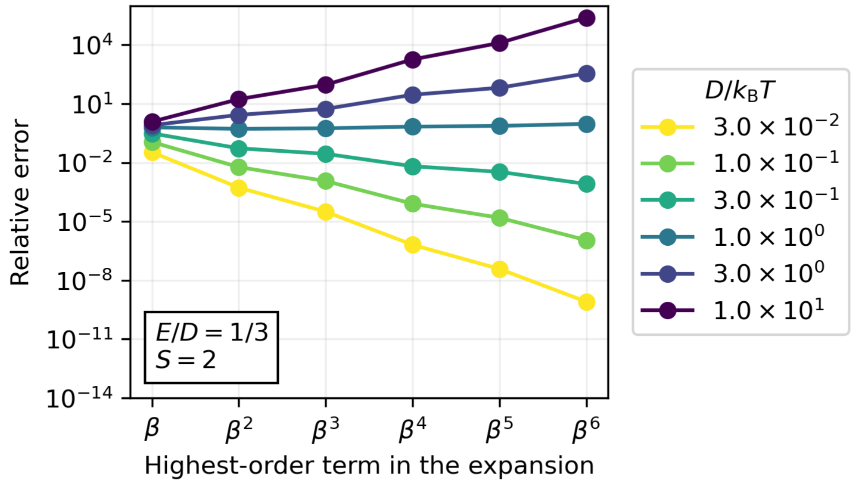

Figure S34: Relative error in the dyadic of a TM complex with $S = 2$, $E/D = 1/3$ and positive D when it is truncated at different orders in β and evaluated at different $D/k_\mathrm{B}T$.

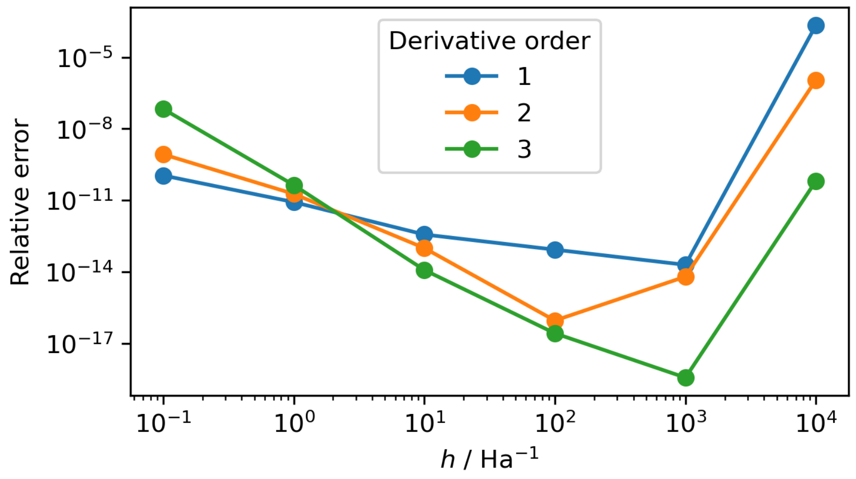

Figure S35: Relative error in the first three numerical derivatives of the spin dyadic as a function of the finite difference h. TM case with $S = 2$, $E/D = 1/3$ and $D = -6$ cm^{-1}.

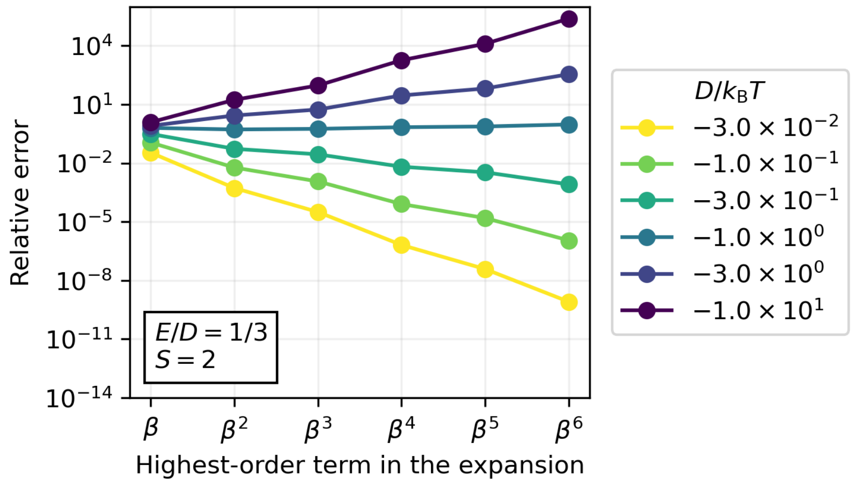

Figure S36: Relative error in the dyadic of a TM complex with $S = 2$, $E/D = 1/3$ and negative D when it is truncated at different orders in β and evaluated at different $D/k_\mathrm{B}T$.

S4.1.4 $S = 5/2$

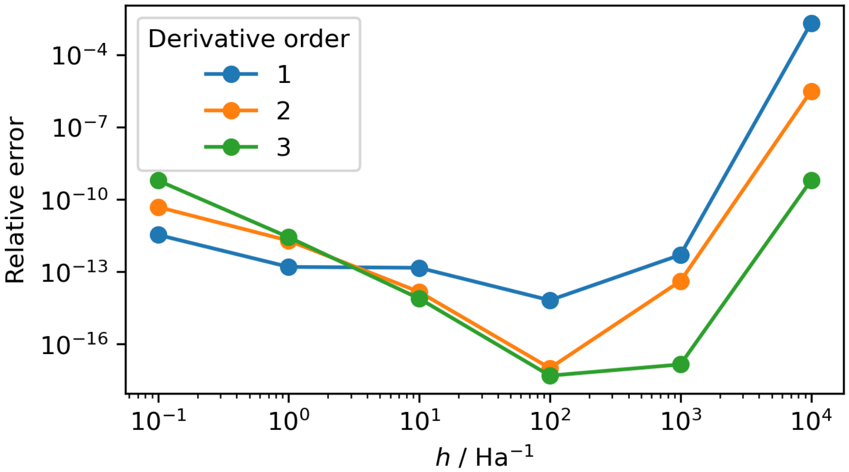

Figure S37: Relative error in the first three numerical derivatives of the spin dyadic as a function of the finite difference h. TM case with $S = 5/2$, $E/D = 0$ and $D = 6$ cm^{-1}.

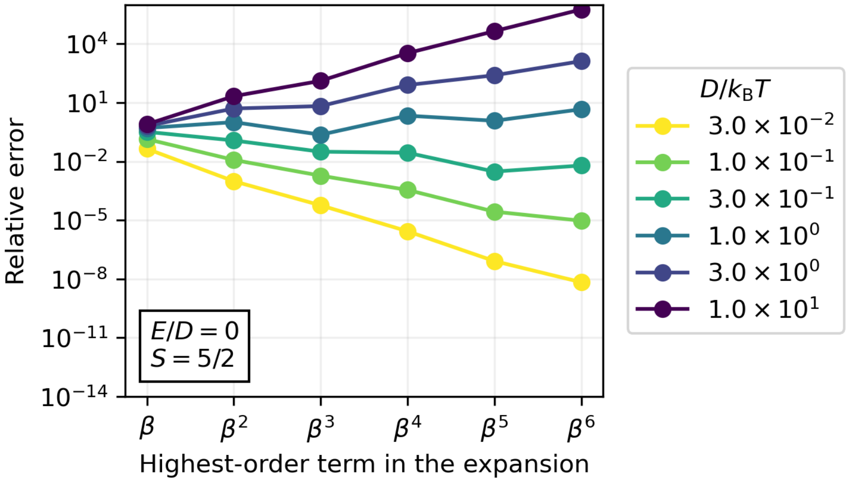

Figure S38: Relative error in the dyadic of a TM complex with $S = 5/2$, $E/D = 0$ and positive D when it is truncated at different orders in β and evaluated at different $D/k_{\mathrm{B}}T$.

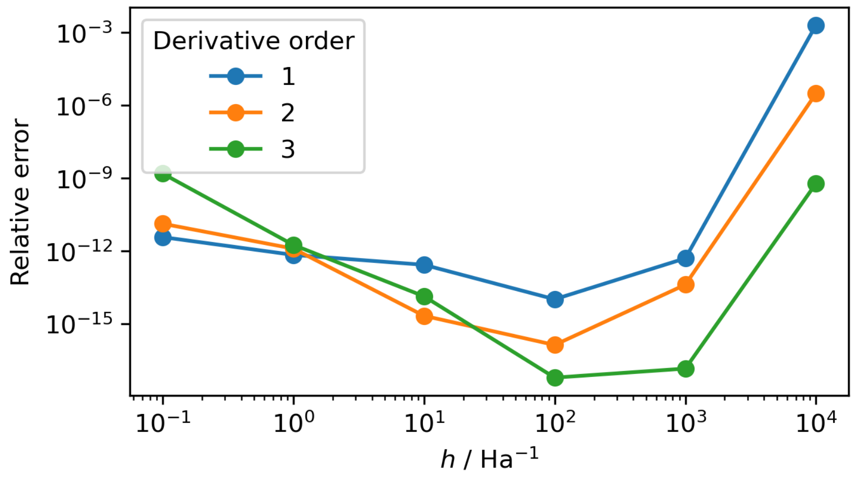

Figure S39: Relative error in the first three numerical derivatives of the spin dyadic as a function of the finite difference h. TM case with $S = 5/2$, $E/D = 0$ and $D = -6\ \mathrm{cm}^{-1}$.

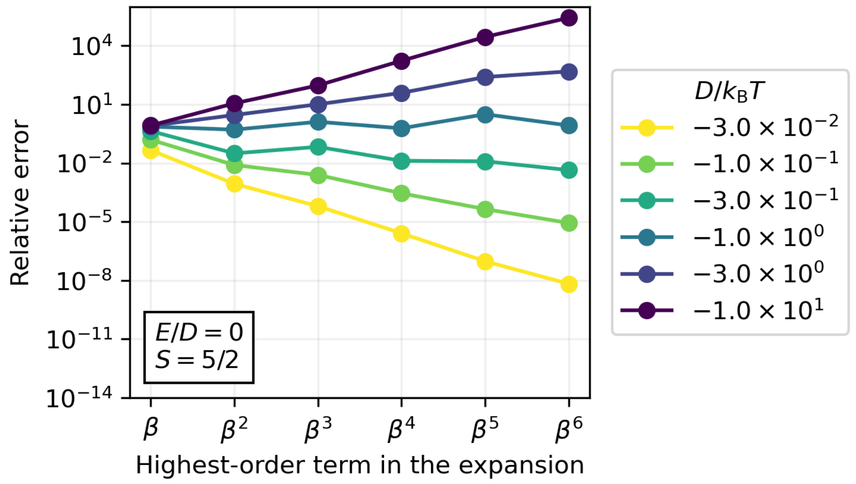

Figure S40: Relative error in the dyadic of a TM complex with $S = 5/2$, $E/D = 0$ and negative D when it is truncated at different orders in β and evaluated at different $D/k_\mathrm{B}T$.

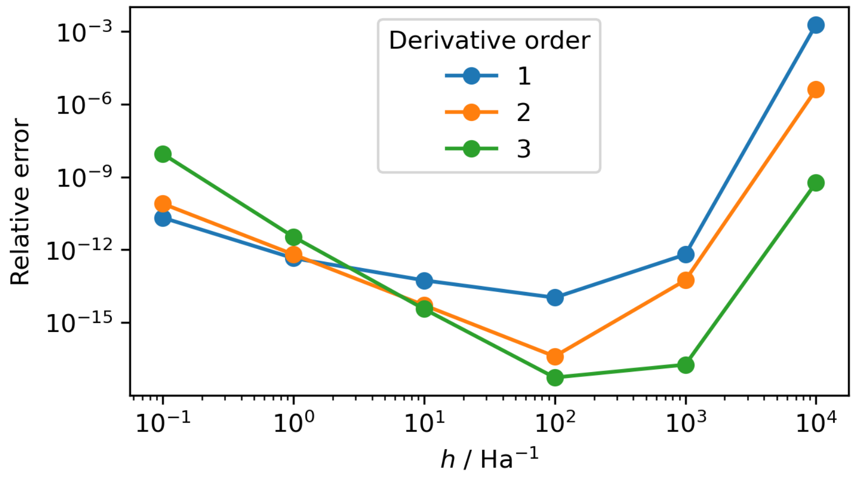

Figure S41: Relative error in the first three numerical derivatives of the spin dyadic as a function of the finite difference h. TM case with $S = 5/2$, $E/D = 1/6$ and $D = 6\ \mathrm{cm}^{-1}$.

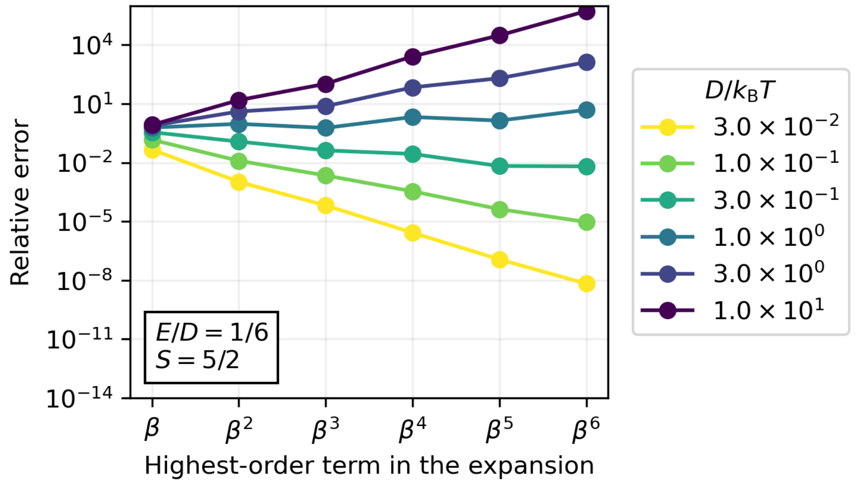

Figure S42: Relative error in the dyadic of a TM complex with $S = 5/2$, $E/D = 1/6$ and positive D when it is truncated at different orders in β and evaluated at different $D/k_\mathrm{B}T$.

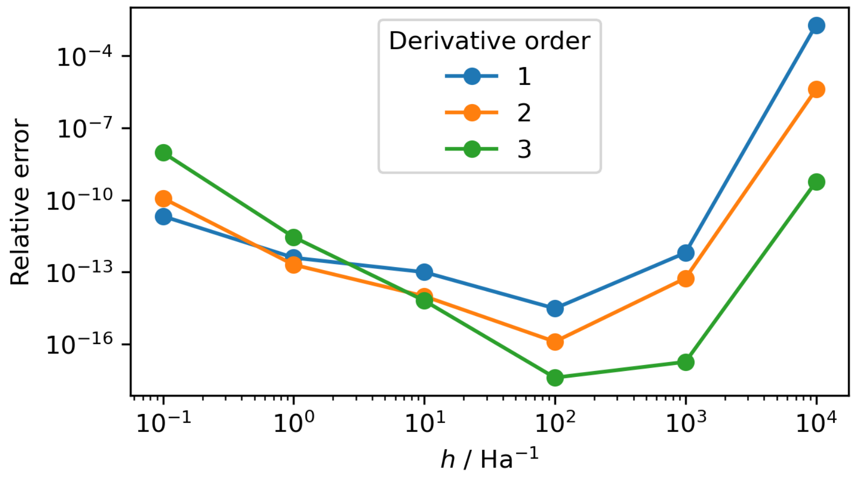

Figure S43: Relative error in the first three numerical derivatives of the spin dyadic as a function of the finite difference h. TM case with $S = 5/2$, $E/D = 1/6$ and $D = -6\ \mathrm{cm}^{-1}$.

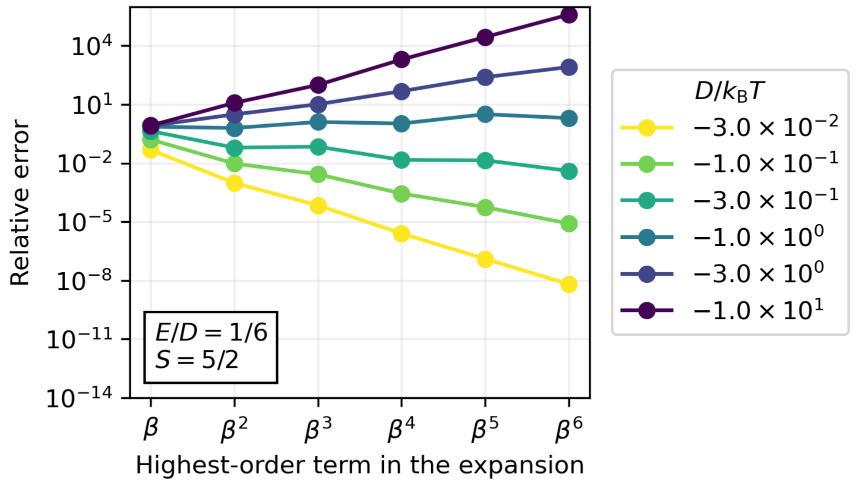

Figure S44: Relative error in the dyadic of a TM complex with $S = 5/2$, $E/D = 1/6$ and negative D when it is truncated at different orders in β and evaluated at different $D/k_\mathrm{B}T$.

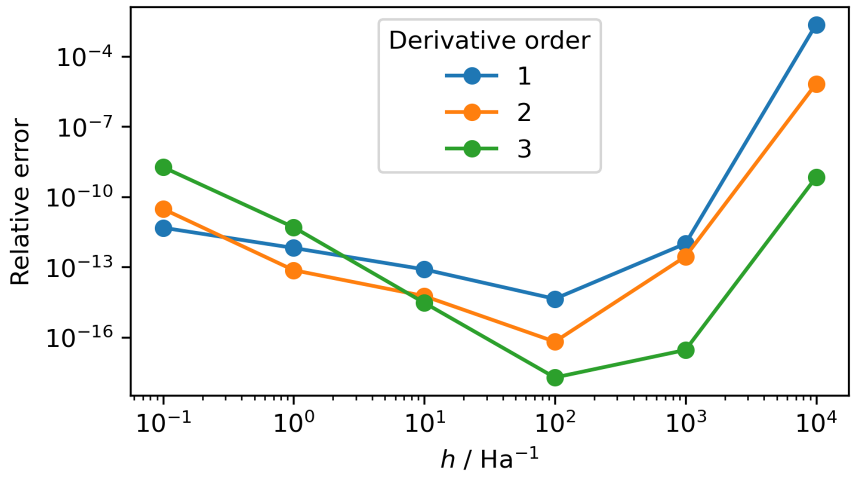

Figure S45: Relative error in the first three numerical derivatives of the spin dyadic as a function of the finite difference h. TM case with $S = 5/2$, $E/D = 1/3$ and $D = 6$ cm^{-1}.

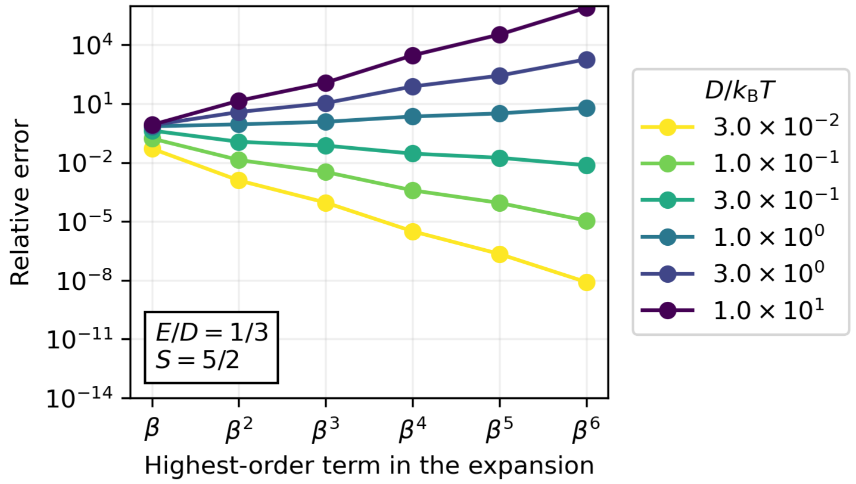

Figure S46: Relative error in the dyadic of a TM complex with $S = 5/2$, $E/D = 1/3$ and positive D when it is truncated at different orders in β and evaluated at different $D/k_\mathrm{B}T$.

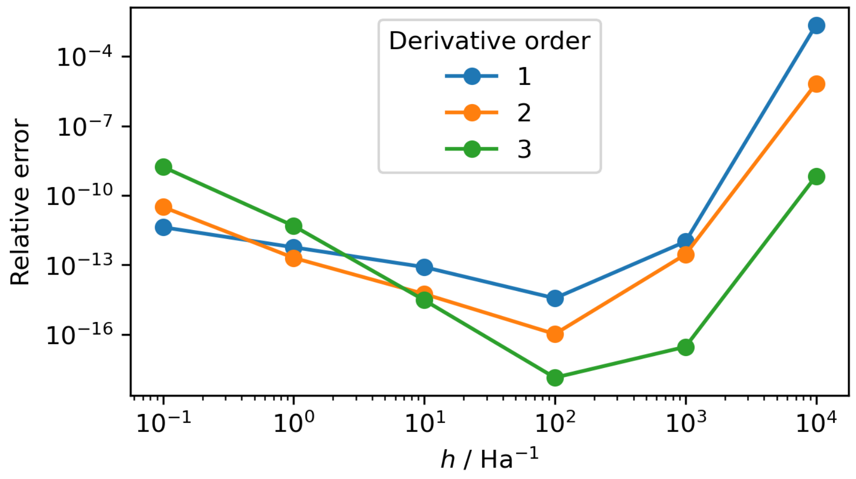

Figure S47: Relative error in the first three numerical derivatives of the spin dyadic as a function of the finite difference h. TM case with $S = 5/2$, $E/D = 1/3$ and $D = -6$ cm^{-1}.

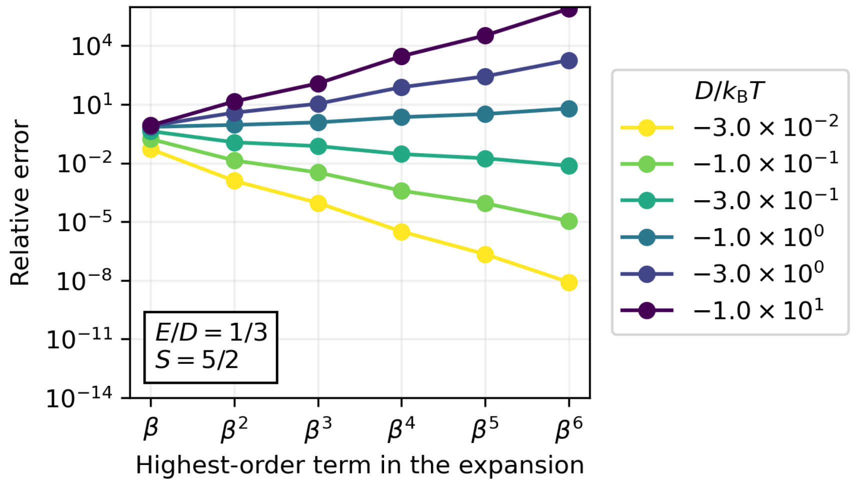

Figure S48: Relative error in the dyadic of a TM complex with $S = 5/2$, $E/D = 1/3$ and negative D when it is truncated at different orders in β and evaluated at different $D/k_\mathrm{B}T$.

S4.2 Lanthanide series

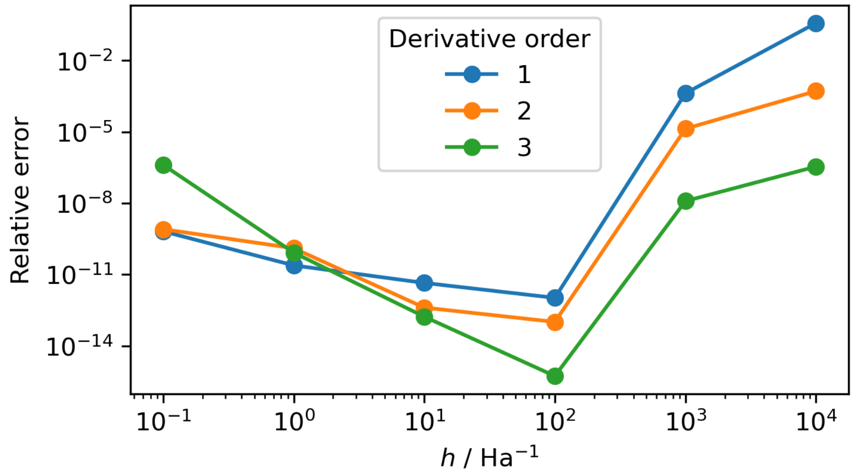

Figure S49: Relative error in the first three numerical derivatives of the spin dyadic as a function of the finite difference h for the Tb(III) complex.

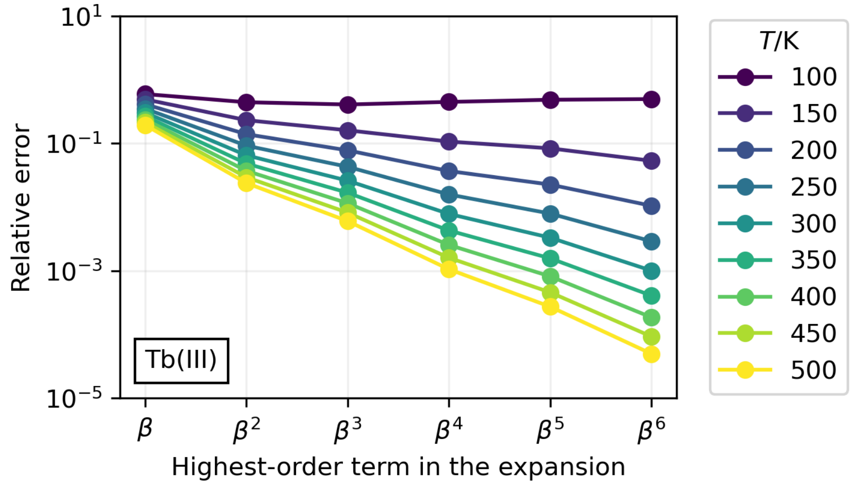

Figure S50: Relative error in the dyadic of the Tb(III) complex when it is truncated at different orders in β and evaluated at different temperatures.

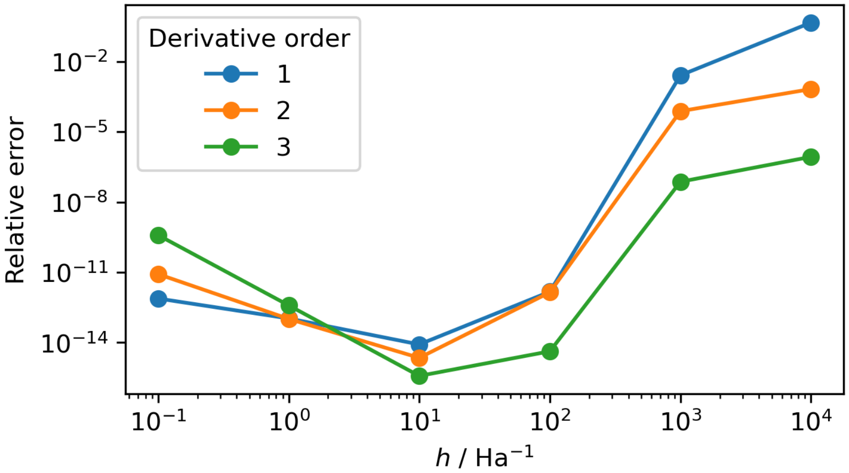

Figure S51: Relative error in the first three numerical derivatives of the spin dyadic as a function of the finite difference h for the Dy(III) complex.

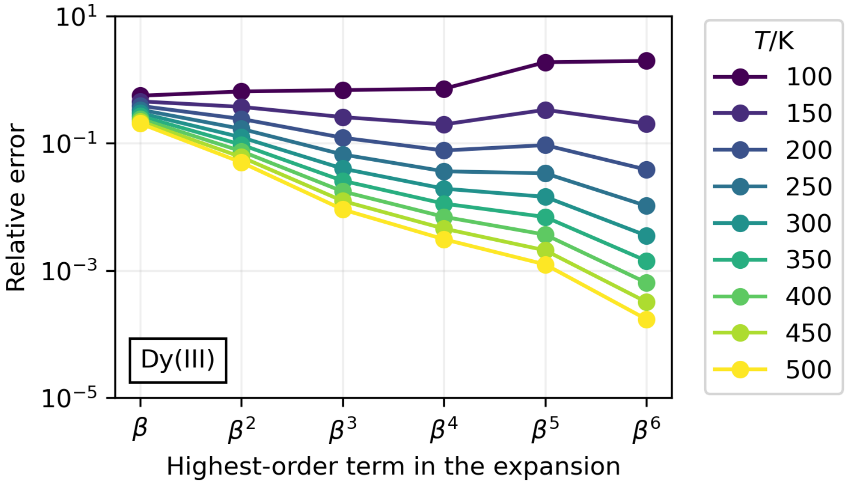

Figure S52: Relative error in the dyadic of the Dy(III) complex when it is truncated at different orders in β and evaluated at different temperatures.

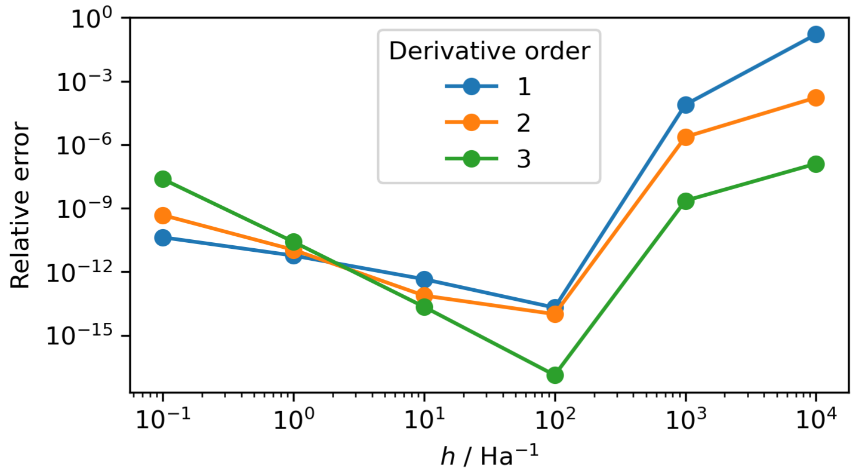

Figure S53: Relative error in the first three numerical derivatives of the spin dyadic as a function of the finite difference h for the Ho(III) complex.

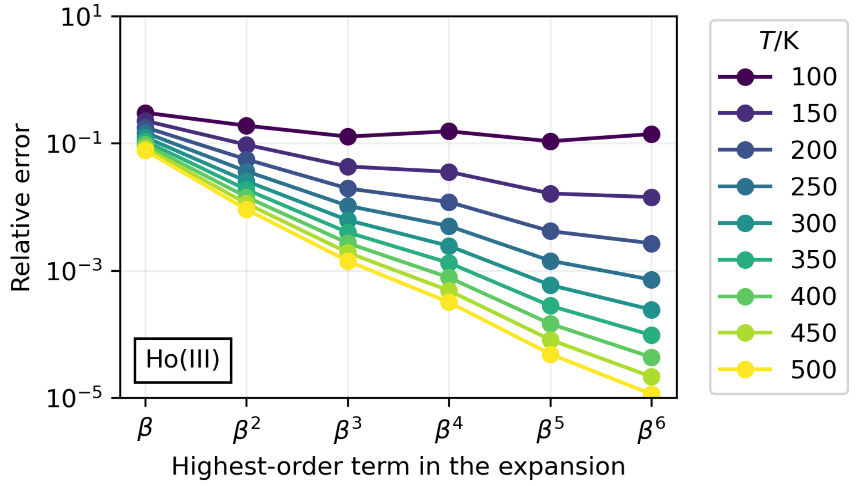

Figure S54: Relative error in the dyadic of the Ho(III) complex when it is truncated at different orders in β and evaluated at different temperatures.

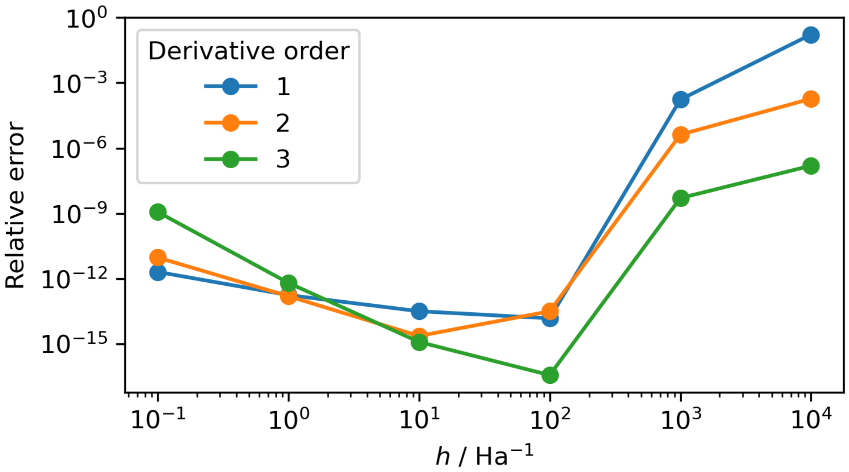

Figure S55: Relative error in the first three numerical derivatives of the spin dyadic as a function of the finite difference h for the Er(III) complex.

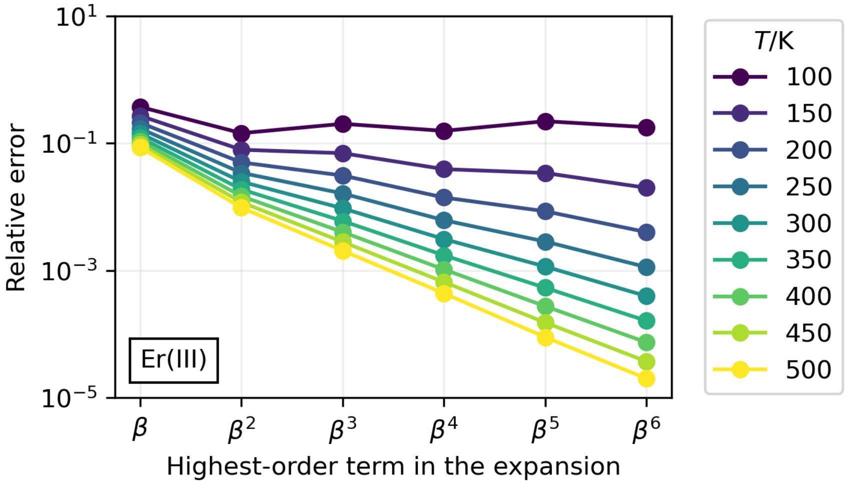

Figure S56: Relative error in the dyadic of the Er(III) complex when it is truncated at different orders in β and evaluated at different temperatures.

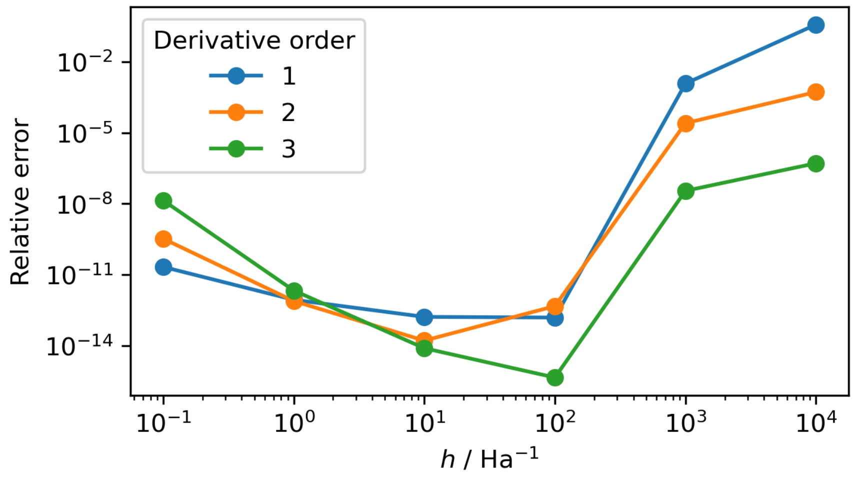

Figure S57: Relative error in the first three numerical derivatives of the spin dyadic as a function of the finite difference h for the Tm(III) complex.

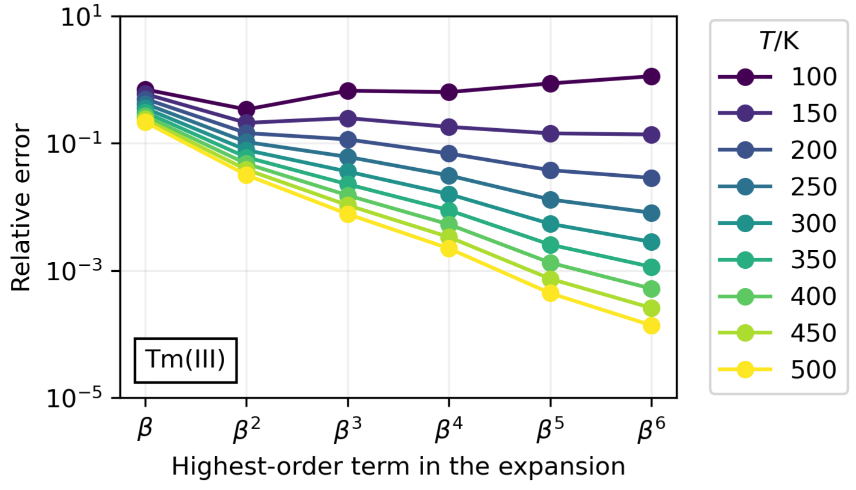

Figure S58: Relative error in the dyadic of the Tm(III) complex when it is truncated at different orders in β and evaluated at different temperatures.

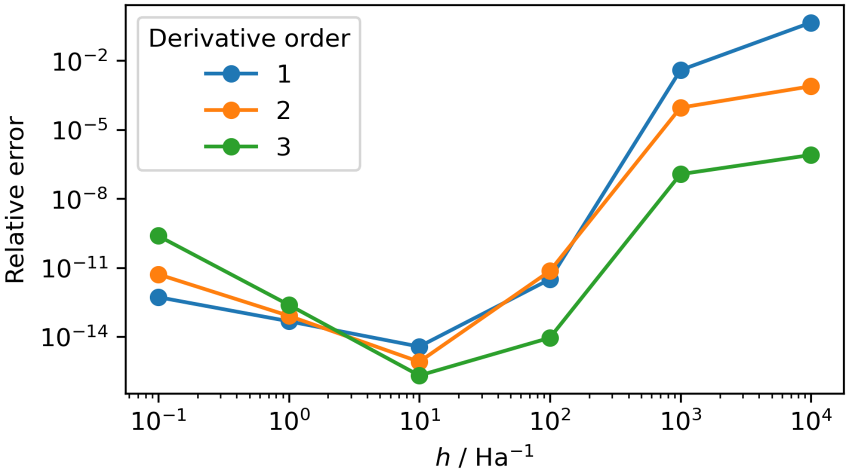

Figure S59: Relative error in the first three numerical derivatives of the spin dyadic as a function of the finite difference h for the Yb(III) complex.

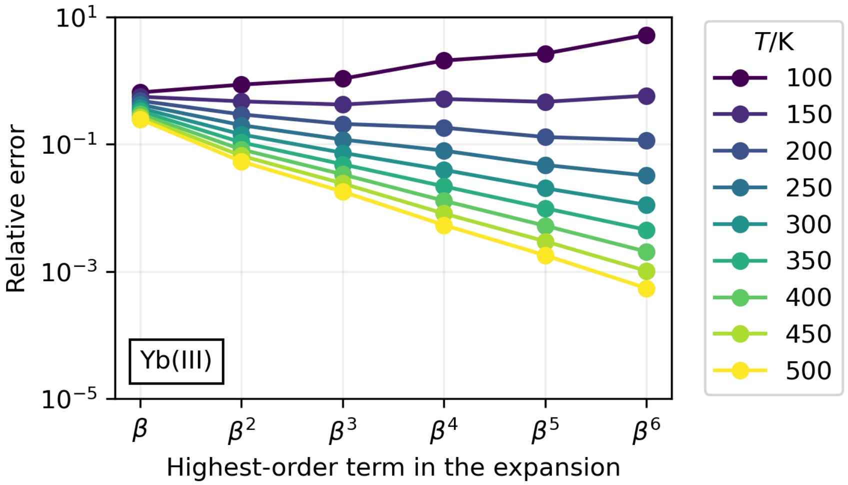

Figure S60: Relative error in the dyadic of the Yb(III) complex when it is truncated at different orders in β and evaluated at different temperatures.

References

(1) Martin, B.; Autschbach, J. Temperature dependence of contact and dipolar NMR chemical shifts in paramagnetic molecules. *J. Chem. Phys.* **2015**, *142*, 054108.
